# A novel quenched fluorescent activity-based probe reveals caspase-3 activity in the endoplasmic reticulum during apoptosis†
†Electronic supplementary information (ESI) available. See DOI: 10.1039/c5sc03207e


**DOI:** 10.1039/c5sc03207e

**Published:** 2015-11-11

**Authors:** Yulia Shaulov-Rotem, Emmanuelle Merquiol, Tommy Weiss-Sadan, Ofra Moshel, Seth Salpeter, Doron Shabat, Farnusch Kaschani, Markus Kaiser, Galia Blum

**Affiliations:** a Institute of Drug Research , The School of Pharmacy , The Faculty of Medicine , Campus Ein Karem , The Hebrew University , Jerusalem , 9112001 , Israel; b School of Chemistry , Faculty of Exact Sciences , Tel-Aviv University , Tel Aviv , 69978 , Israel; c Department of Chemical Biology , University of Duisburg-Essen , Center for Medical Biotechnology , Faculty of Biology , 45117 Essen , Germany

## Abstract

The caspases are a family of cysteine proteases that are key regulators of apoptosis and their activity may thus serve as a good marker to monitor cell death. We have developed a quenched fluorescent activity-based probe (qABP) that is selective for caspase-3 activity and emits a fluorescent signal after covalently modifying its target. The probe has a wide range of potential applications, *e.g.* in real-time imaging, FACS analysis or biochemical quantification of caspase activity in intact cells. Application of the probe allowed us to monitor caspase-3 activation after chemotherapy-treatment and to distinguish between apoptosis sensitive and resistant cells. Moreover, it enabled real-time high-resolution visualization of active caspase-3 during apoptosis. This led to the surprising finding that in cancerous cells active caspase-3 is not only found at the familiar cellular locations but also in mitochondria and the endoplasmic reticulum. Thus, our novel covalent probe allows high spatial and temporal resolution imaging of caspase-3 activation and may thus be used as an effective tool to study molecular mechanisms of programmed cell death in healthy and disease states.

## Abstract 2

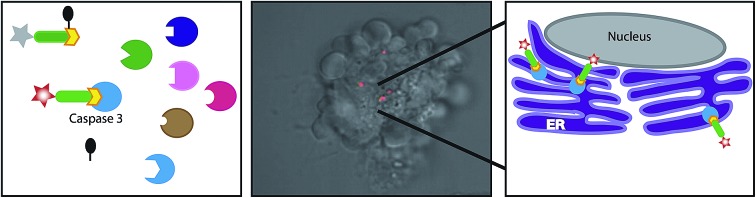
A selective quenched activity-based probe detects caspase-3 activity in the endoplasmic reticulum of cancerous cells during apoptosis.

## Introduction

Apoptosis (programmed cell death) is the major process by which multi-cellular organisms eliminate excessive, damaged and potentially dangerous cells.^1^ Both normal and pathological processes such as embryonic development, cancer, auto-immune disorders, ischemia and reperfusion or Parkinson's and Alzheimer's disease involve apoptotic cell death processes. Importantly, the induction of apoptosis in cancerous cells is the desired outcome of many cancer chemotherapeutic treatments and initiation or inhibition of apoptosis is a key element in numerous therapies.^2^,^3^ Therefore, the availability of imaging tools for tracking cell death immediately after treatment would not only be desirable for basic research but also of great benefit for evaluating therapy success.

The caspases are a family of cysteine proteases that are critical for the execution of apoptosis. They are divided into two sub-families: the initiator caspases (caspases-2, -8, -9 and -10) which are initially activated by specific death stimuli from receptors or the mitochondria and the effector caspases (caspases-3, -6 and -7) that are triggered in response to initiator activation and overtake extensive substrate proteolysis leading finally to cellular destruction and death.^4^ Caspase-3 is a key mediator of the apoptotic process and the most proficient caspase, featuring an astonishing low *K*_M_ and high *k*_cat_ for several substrates.^5^ The inactive zymogen form of caspase-3 is a dimer and activation is post-translationally regulated by distinct cleavages of a short interdomain linker between the large and small subunits.^6^,^7^ These proteolytic events enable a rearrangement of the activation loops in the structure and thus the formation of the active sites for substrate cleavage.^3^

Under normal physiologic conditions, caspase-3 is localized in the cytoplasm in its inactive zymogen form. Caspase-3 activation occurs in the cytosol, active caspase-3 is then translocated to the nucleus. In contrast, cellular mislocalization of active caspase-3 can lead to apoptosis resistance.^8^ However, there is still not much known about the kinetics of caspase-3 activation, the exact sub-cellular localizations of caspase-3 and the precise molecular mechanism of active caspase-3 translocation to the nucleus.^9^,^10^ Consequently, tools for monitoring caspase-3 at high resolution in real-time would be highly desirable to better understand the dynamics of caspase-3 activation, most importantly in the context of disease.

Different strategies for monitoring caspases or more specifically caspase-3 activity have already been developed using chemical biology methods. For example, chemical biology approaches for directed activation or for identifying caspase substrates have been developed consisting for example of active caspase specific antibodies or small molecule probes and substrates also containing non-natural amino acids.^11^–^15^ In particular fluorescent activity based probes such as FLICA, a fluorophore-labeled fluoromethyl ketone probe^16^,^17^ or AB50, an acyloxy methyl ketone based probe,^18^ have been generated and applied to monitor caspase activity in cells and small animals. Additional fluorescent substrate probes such as TCapQ,^19^,^20^ NucView^21^ and split bioluminescence caspase probes have also been developed.^22^,^23^ Unfortunately, activity based probes as well as antibody based probes create the need for washes and manipulation of samples prior to imaging, preventing real-time imaging and manipulation-free examination. Furthermore, substrate probes often also target other cysteine proteases such as cathepsin B or legumain,^24^ and diffuse away from their target enzyme, thereby diminishing high resolution imaging capabilities.^5^

**Fig. 1:**
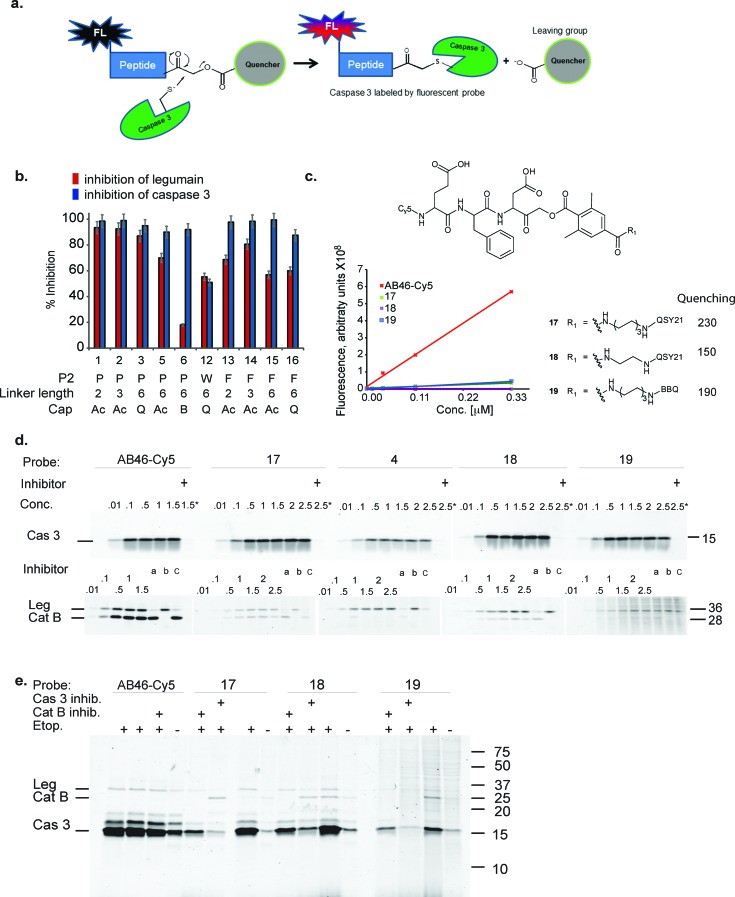
First generation quenched activity based probes development. (a) General scheme for the mechanism of action of a fluorescent quenched activity based probe (qABP). (b) Inhibition studies of recombinant caspase-3 *vs.* inhibition of legumain in RAW cell lysates with the different inhibitors described in Table 1. (c) Chemical structure, fluorescence intensity and quenching efficiency of probes **17**, **18** and **19**. (d) Direct labeling of recombinant caspase-3 (upper panel) and legumain and cathepsin B in RAW cell lysate (lower panel) by indicated qABPs. Recombinant caspase-3 was incubated with increasing probe concentrations for one hour, the reaction was stopped and separated on a SDS PAGE and scanned for Cy5 fluorescence. Samples marked with ^+^ were pretreated with a caspase inhibitor (AB46 peptide) 30 min prior to the probe treatment. Legumain and cathepsin B from RAW cell lysates were labeled by the indicated qABPs similarly to caspase labeling. Samples marked with ^a, b^ or ^c^ were pretreated for 30 min with the inhibitors AB46 peptide, GB111-NH_2_ 25 or **5** to selectively block caspase-3; cathepsin B or legumain, respectively. (e) Direct labeling of active caspase-3 in intact MM1s cells undergoing apoptosis. The indicated qABP showed covalent binding to active caspase-3, seen at 17 kDa. Samples marked with ^+^ represent the pretreatment with a caspase-3/legumain inhibitor (AB46 peptide) or cathepsin B inhibitor (GB111-NH_2_) which was added 1 h prior to the probe.

To overcome these limitations and to generate a true high-resolution real-time imaging tool, we therefore developed a selective quenched fluorescent activity based probe (qABP) for caspase-3. We have based our design on our previously published qABPs strategy,^25^,^26^ the non-quenched caspase probes AB50-Cy5 18 and the legumain probe LE28.^27^ Quenched fluorescent ABPs are small molecules that covalently bind their target in an activity dependent manner. A short sequence in the probe drives recognition by the target and an electrophile termed the “warhead” covalently binds the target enzyme. Unlike regular fluorescent ABPs, quenched probes include an additional quencher unit that is removed when the target protease binds to the probe. Therefore, upon binding to the activated enzyme, the probe transitions from a non-fluorescent free probe to an enzyme bound fluorescent complex^25^ (Fig. 1a). In the present study, we generated a selective caspase-3 quenched probe without cross-reactivity with cathepsin B and legumain by modifying both the prime and non-prime probe regions. The selective caspase-3 qABP was then applied to time lapse systems to study cellular localization at high resolution over time in both apoptosis sensitive and resistant cancer cells. Interestingly, we thereby discovered novel caspase-3 activity patterns in these cell populations that were subsequently validated biochemically.

## Results

### Development of selective caspase-3 qABPs and their *in vitro* evaluation

We set out to generate selective qABPs for caspase-3. We based our initial design on probes from the Bogyo group: AB46-Cy5, a non-quenched probe for caspase-3,^18^ AB50-Cy5 18 and LE28,^27^ and cathepsin quenched probes.^25^,^26^ AB46-Cy5 (Cy5-E8D-AOMK-DMBA, 8 stands for 2-amino butyric acid, see Table 1, bottom) was designed to be an ABP for caspase-3 but displayed cross-reactivity with legumain and cathepsin B. LE28 is a qABP based on AB50-Cy5 that targets both legumain and caspase-3 and contains a Cy5 fluorophore linked to a Glu–Pro–Asp (P3–P2–P1) peptide scaffold and an acyloxymethyl ketone dimethylterephthalate propane linker attached to a quencher moiety (structures in Table 1, bottom). It is obvious that the cross reactivity to the two lysosomal cysteine proteases cathepsin B and legumain significantly lower the usage of caspase probes turning the development of more selective compounds highly attractable.

**Table 1:** The compounds differ in their peptide sequence at the P2 position; R_1_ represents the corresponding side chain at this P2 position in the probe sequence E-P2-D. *R_2_ represents an acyl group or one of the two quenchers, *i.e.* QSY21 or BBQ (Blackberry quencher). **R_3_ indicates if the compound was fluorescently labeled or not. ***n denotes the number of (CH_2_) units and thus the length of diaminolinker, % ACN denotes the percentage of acetonitrile at which the compound eluted from the analytical HPLC. All synthesized compounds were purified *via* C-18 or C-4 preparative RP column after each synthetic step and characterized by LCMS. The final products were obtained in 3–32% yield after the final step of isolation and in over 95% purity (*i.e.* giving a single peak in the chromatogram at 215 as well as 254 nm)

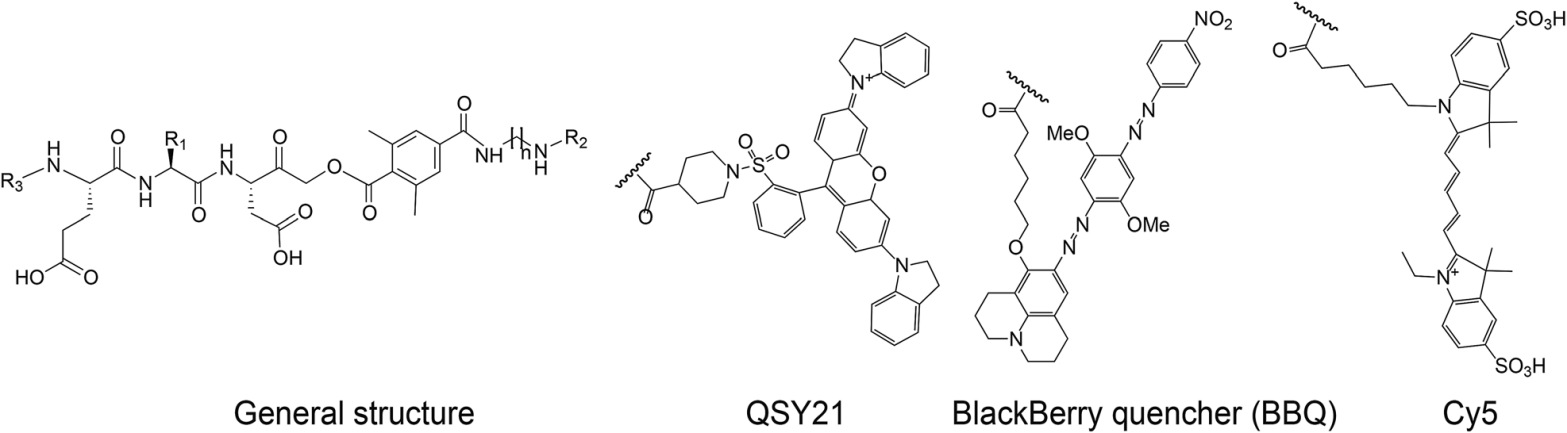									
	Compound	R_1_ side chain	*R_2_	**R_3_	***n	% ACN	MW (g mol^–1^)	Synthetic route	% Yield
1	YS4/131A	P	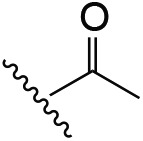	H	2	20	634	2	18
2	YS4/119B	P	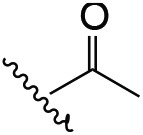	H	3	13	647	2	20
3	YS5/15	P	QSY21	H	6	48	1311	1	7
4	GB300	P	QSY21	Cy5	6	51	1952	1	8
5	YS4/105	P	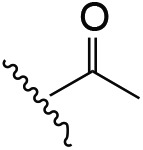	H	6	18	689	1	5
6	YS5/60	P	BBQ	H	6	59	1245	2	7
7	YS4/85	W	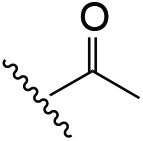	H	2	15	723	1	8
8	YS4/143	W	QSY21	H	2	43	1344	2	11
9	YS4/119A	W	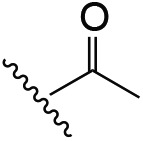	H	3	20	736	2	30
10	YS4/141	W	QSY21	H	3	40	1358	2	28
11	YS4/103	W	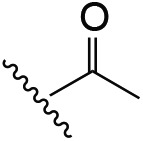	H	6	23	778	1	4
12	YS4/142	W	QSY21	H	6	50	1400	2	3
13	YS4/131B	F	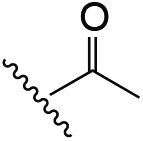	H	2	13	683	2	4
14	YS4/138	F	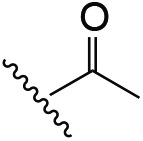	H	3	19	697	2	32
15	YS4/137	F	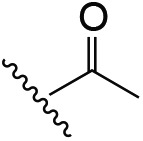	H	6	23	739	2	17
16	YS4/140	F	QSY21	H	6	43	1362	2	29
17	YS5/51	F	QSY21	Cy5	6	71	2000	2	14
18	YS5/67	F	QSY21	Cy5	2	61	1944	2	4
19	YS5/70	F	BBQ	Cy5	6	45	1934	2	9
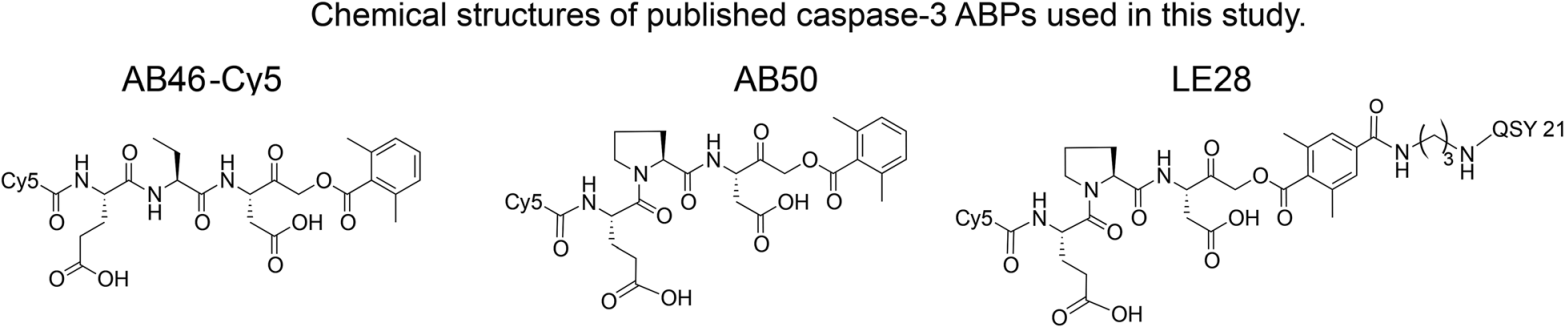									

The selective caspase-3 qABP was generated in a stepwise manner. First, inhibitors lacking the fluorophore were synthesized and biochemically evaluated for their binding to caspase-3 and legumain (Fig. 1b). The most potent and selective inhibitors were then converted into probes by attachment of a fluorophore to the inhibitor. We started by generating a library of inhibitors with varied P2 position and the quencher and/or linker moieties in the prime site of LE28. Tryptophan and phenylalanine were selected as P2 amino acids based on their reported weak legumain interactions.^28^ We therefore synthesized EWD, EPD, EFD (P3–P2–P1) sequences and incorporated diamino-linkers of different length and various quenchers in the prime site (see general structures of compounds **1–16** in Table 1).

The tripeptide was generated on solid support using solid phase peptide synthesis (SPPS) similar to previously described methods.^26^ The protected tripeptide was cleaved from the resin and converted to a chloromethylketone (CMK) using diazomethane. It was then coupled to different resin bound 2,6-dimethyl-terephthalic-amides – that were generated from the various diamino linkers, thus forming the acyloxymethyl ketone (AOMK) warhead (ESI Scheme 1†). The product was then cleaved using mild acidic conditions for maintaining the Boc and *t*-Bu side chain protections. The free amine was acetylated or coupled to QSY21 or BlackBerry 650 (BBQ), thereby incorporating the quencher units into the probe library. All compounds were purified by preparative HPLC and characterized using LCMS. The overall yields of the final products varied between 4–30%.

### Biochemical evaluation of compound library

Inhibitors **1–16** were tested for their inhibitory potency against recombinant caspase-3 and legumain using RAW cell lysates. First, each inhibitor was incubated with RAW cell lysate or recombinant caspase-3 enzyme and the residual enzyme activity was assayed with **4** (a caspase-3 activity based probe that was found nonspecific for legumain activity, data not shown). The bound and free probe were separated by SDS-PAGE and scanned for Cy5 fluorescence (Fig. 1b). Compounds **7–11** with tryptophan at P2 inhibited legumain over 50% at low concentrations (ESI Fig. 1†); they were not further evaluated. All compounds except compound **12** (EWD) showed over 90% inhibition of caspase-3. We found that most EPD based inhibitors displayed the highest legumain inhibition (∼90%), followed by EFD (∼60%) based inhibitors, whereas lower inhibition was observed for EWD-based compound **12**. This finding demonstrates that changing P2 from proline to phenylalanine reduces the binding affinity for legumain. The reduced potency of the P2 tryptophan compound for caspase-3 might be caused by a steric clash and unfavorable hydrophobic interactions.

Additionally, the change of the quencher moiety from QSY21 in **3** (EPD) to Blackberry in **6** (EPD) significantly reduced the binding affinity towards legumain. This led us to conclude that legumain reactivity can also be affected by the interaction of the inhibitor with the prime site of the enzyme and not only with the non-prime site. We then attached Cy5 to selected EFD containing inhibitors and continued investigating direct labeling of these enzymes (see structures in Fig. 1c).

The quenched probes **17**, **18** and **19** were found to have dramatically reduced fluorescence when compared to the non-quenched probe AB46-Cy5, with AB46-Cy5 showing 230-fold, 150-fold and 190-fold higher fluorescence, respectively (Fig. 1c). Direct labeling of recombinant caspase-3 (Fig. 1d, upper panel) and legumain in RAW cell extracts (Fig. 1d, lower panel) was evaluated for these probes. The enzyme or lysates were incubated with increasing probe concentrations, with or without pretreatment of specific inhibitors for caspase, legumain or cathepsins. The free probes were separated from the enzyme-bound probe using gel electrophoresis after which the gel was scanned for fluorescence (Fig. 1d). We found legumain (36 kDa) in the upper band and cathepsin B (28 kDa) in the lower band of labeled RAW cell lysate. While AB46-Cy5 maximal labeling of caspase-3 was achieved at a concentration of 0.1 μM, probes **17–19** were less potent and reached their maximum labeling at 0.5 μM. Notably, they were considerably less potent towards legumain and cathepsin B in the tested concentration range.

We next tested the probe's ability to selectively label active caspase-3 in intact MM1s cells that were induced to undergo apoptosis by treatment with etoposide, a cytotoxic anticancer topoisomerase inhibitor.^29^ After apoptosis induction, the cells were pre-treated with vehicle, caspase-3 or cathepsin B inhibitors, followed by AB46-Cy5 or the qABPs **17**, **18** or **19** (Fig. 1e). The fluorescent probes were found to covalently bind active caspase-3, detected by a 17 kDa band on the gel. While AB46-Cy5 also targeted legumain, probe **19** labeled legumain only very weakly. Labeling of legumain by AB46-Cy5 is not surprising since P1 aspartic acid probes have been reported to bind legumain.^28^ Unfortunately, cathepsin B was also labeled by probes **17–19**, although in significantly less intensity than caspase-3, as seen by the cathepsin inhibitor GB111-NH_2_ 25 sensitive 28 kDa protein band.

**Scheme 1:**
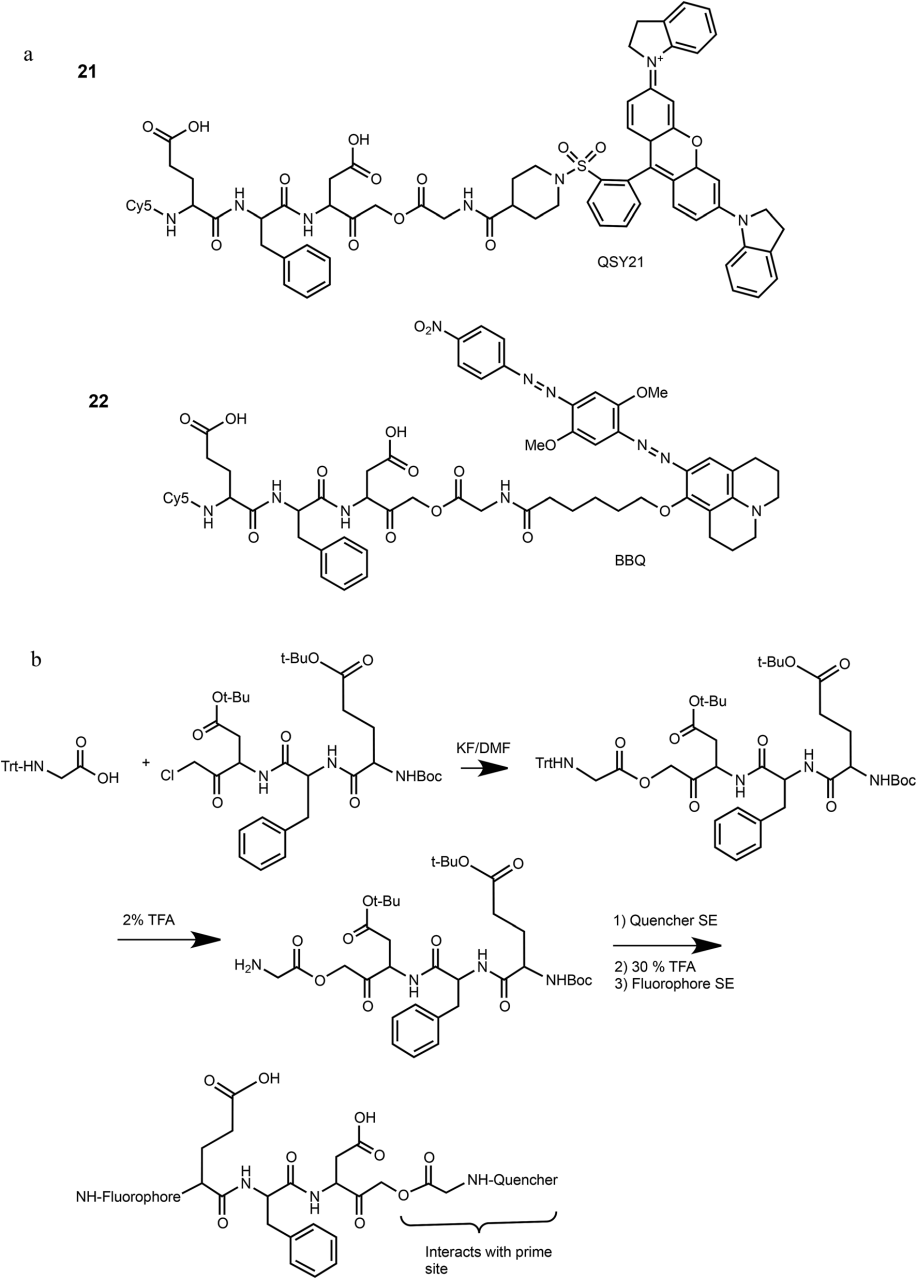
Structures and synthetic route of specific caspase-3 qABPs **21** and **22**.

We have previously reported that cathepsin B has reduced affinity to compounds with steric hindrance in the prime site.^26^ Therefore, we modified the primed binding site of probes **18** and **19** in an attempt to lower cathepsin B binding affinity. We generated two glycine-AOMK based qABPs: **21** with QSY21 and **22** with BBQ, in which the bulky quencher was incorporated close to the AOMK to induce a steric clash to the prime site of cathepsin B (Scheme 1a). To this end, the tripeptide chloromethyl ketone was condensed with commercial trityl protected glycine. The quencher and fluorophore were then attached sequentially (Scheme 1b and ESI† Methods).

### Biochemical analysis of specific caspase-3 qABPs **21** and **22**

**Fig. 2:**
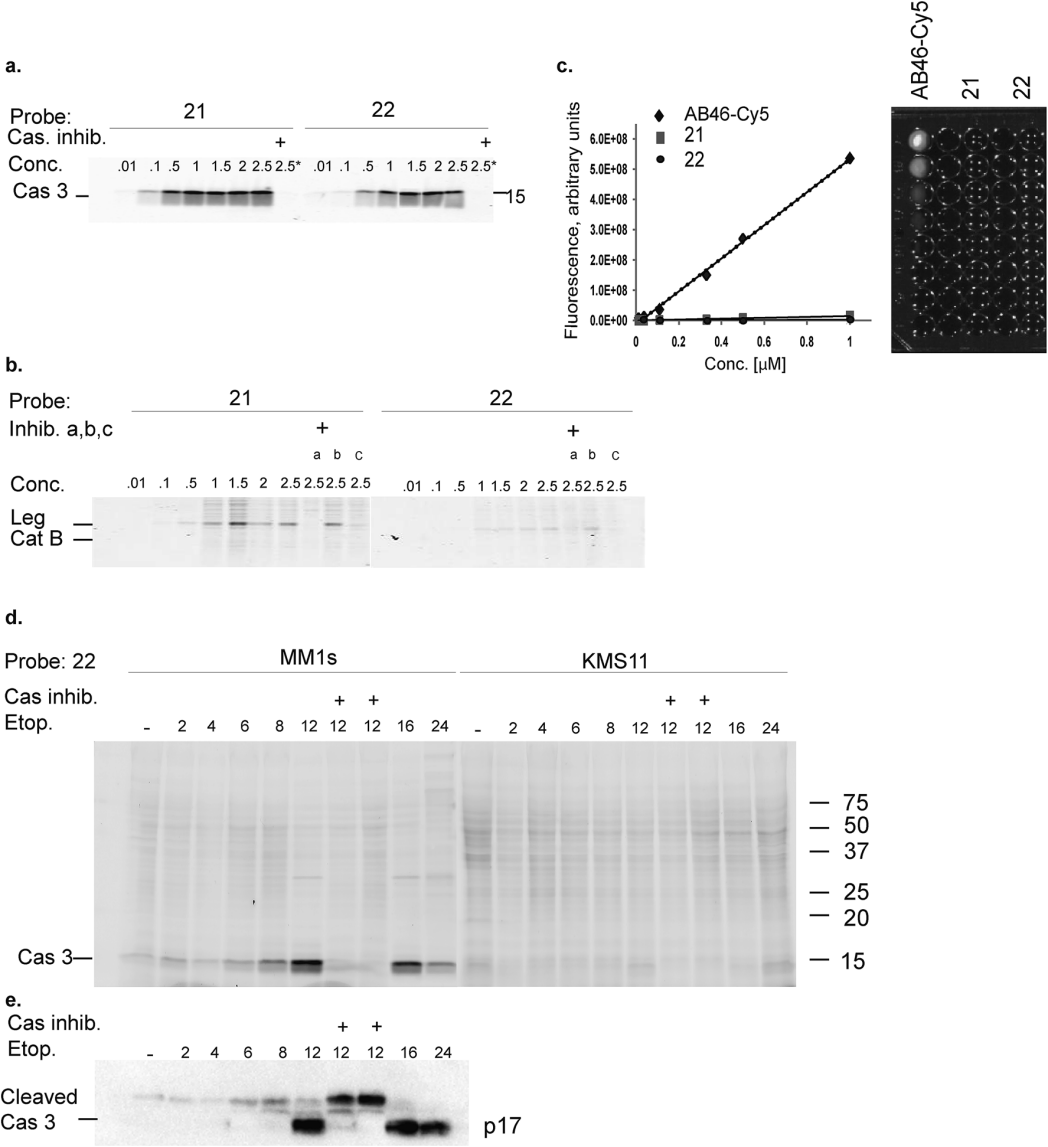
Biochemical evaluation of second generation quenched fluorescent activity based probes. (a) Direct labeling of recombinant caspase-3 with qABPs. The indicated probes were incubated at various concentrations with recombinant caspase-3 for one hour, the reaction was stopped, separated by SDS PAGE and the gel was scanned for fluorescence. Samples marked with ^+^ represent pretreatment with the caspase/legumain inhibitor AB46 peptide which was added 30 min prior to probe. (b) Direct labeling of legumain in RAW cell lysate. Similar labeling conditions as in (a) were used. Samples were pretreated with the following inhibitors for 30 min prior to probe labeling: caspase-3 (AB46 peptide), a, cathepsin GB111-NH_2_, b, or legumain-compound **5**, c. (c) Quenching efficiency. Fluorescence of increasing concentration of qABPs **21** and **22** was measured relative to the non-quenched AB46-Cy5 with an IVIS scanner, excitation/emission 640/695–770 nm. Left panel, a graph showing reduced fluorescence. Right panel, a fluorescent picture of the dilution plate. Both probes were found highly quenched, **21** 50 fold and **22** 150 fold. (d) Time course of caspase-3 labeling in intact cells. Intact MM1s and KMS11 cells were treated with etoposide for indicated times, **22** was added for the last three hours. The caspase inhibitor ZVAD-FMK was added 9 or 3 hours prior to **22**. Protein lysates were loaded and resolved by SDS-PAGE, followed by scanning on a flatbed laser scanner for Cy5 fluorescence. (e) Samples of MM1s cells from d were subjected to western blotting and reacted with a cleaved caspase-3 antibody.

The second generation probes **21** (Cy5-EFD-AOMK-Gly-QSY21) and **22** (Cy5-EFD-AOMK-Gly-BBQ) were tested for their reactivity towards caspase-3, cathepsin B and legumain as previously shown in Fig. 1d. While the potency of compound **21***versus* caspase-3 was comparable to compound **17–19**, compound **22** was slightly less potent (Fig. 2a). However, almost no legumain and cathepsin B labeling was observed with **22** (Fig. 2b) and the quenching efficiency of both probes relative to AB46-Cy5 remained high, over 50-fold and 150-fold for **21** and **22**, respectively (Fig. 2c).

To further evaluate potential cross reactivity towards legumain in intact cells, human Colo-205 colorectal cancer cells that naturally express high levels of legumain and cathepsin B were treated with the various probes. After probe incubation, cell lysates were collected and separated by SDS PAGE and were then scanned for fluorescence. We detected no non-specific labeling with **22** and very weak cathepsin B labeling with **21** (ESI Fig. 2†). As previous studies indicated that it is difficult to achieve selective caspase-3 inhibition due to cross-reactivity with caspase-7,^30^ we evaluated the potency of **22** towards recombinant caspase-7. Although we could detect weak binding of caspase-7 at higher concentrations, this binding does not interfere with caspase-3 labeling (ESI Fig. 3†). Since compound **22** is potent and selective towards caspase-3 and also highly quenched, we selected it for subsequent studies in different biological systems.

### Detecting caspase-3 activity in apoptotic cells using a next generation qABP

Our qABP is designed as a tool for real time visualization of caspase-3 activity during apoptosis. We first determined the optimal time window for detecting caspase-3 activity in apoptotic cells using biochemical methods. We monitored caspase-3 activity after chemotherapy-induced apoptosis in a time course experiment in a time interval of 2 to 24 hours in chemotherapy-resistant KMS11 and sensitive MM1s cells. Apoptosis was initiated with etoposide and probe **22** was added three hours before lysing the cells. Crude lysates were run on an SDS PAGE and labeled enzymes were detected by a fluorescent gel scan (Fig. 2d). The caspase-3 signal was detected as a ∼17 kDa fluorescent band that reached its maximum intensity between 12–16 hours after etoposide treatment in the sensitive MM1s cells. Not surprisingly, we detected very little caspase-3 activity in the resistant KMS11 cells. Probe specificity was verified by addition of the caspase-3 inhibitor ZVAD-FMK and by blotting with a caspase-3 antibody (Fig. 2e). These data show that our probe specifically labels caspase-3 activity in intact cells and can be used to assess the dynamics of apoptosis in biological systems.

**Fig. 3:**
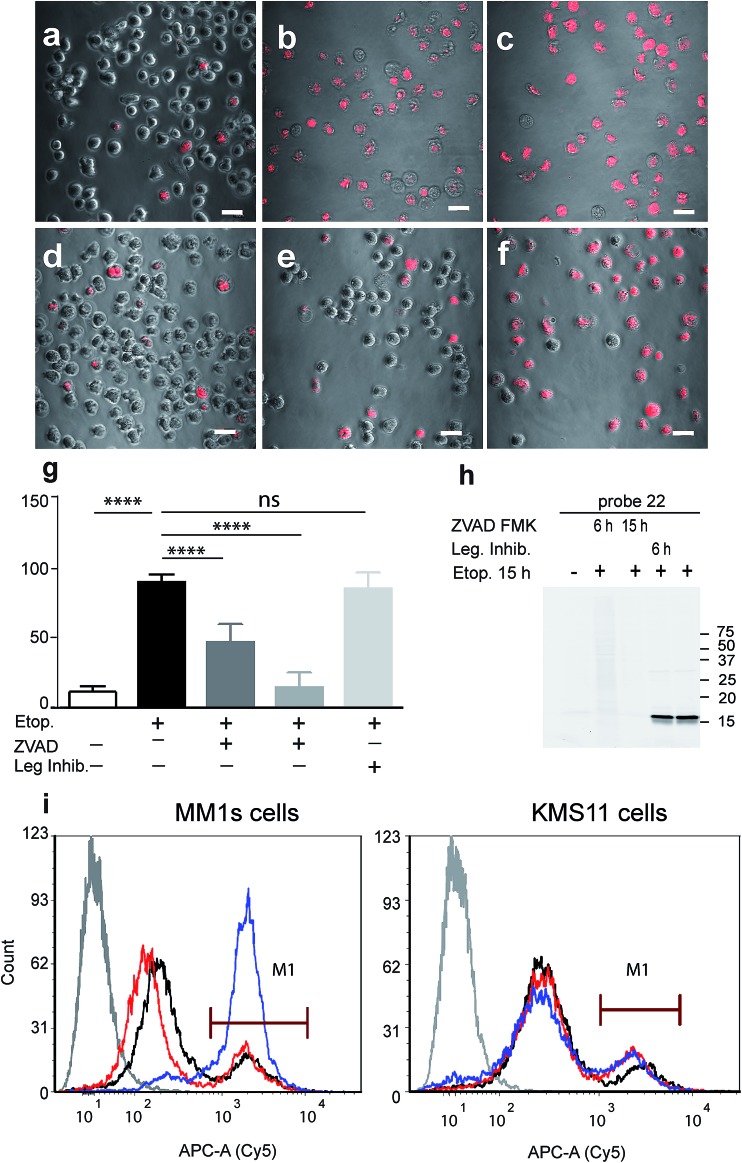
Fluorescent analysis of apoptotic MM1s cells using probe 22. MM1s cells were treated with etoposide for 15 hours, during the last 3 hours probe 22 was added. Cells were pretreated with the caspase inhibitor or legumain inhibitor for indicated times. (a) Control vehicle treated cells, (b) and (c) etoposide treated cells, (d) ZVAD-FMK added with etoposide, (e) ZVAD-FMK added 9 hours after etoposide, (f) legumain inhibitor added 9 hours after etoposide. Cy5 fluorescence (red) overlaid on bright field images were taken of living cells with an Olympus confocal microscope, scale bar = 10 μm. (g) Quantification of labeled cells, the percent of Cy5 labeled cells was quantified by counting the red cells, at least 10 fields were taken of each treatment. (h) Fluorescent gel analysis of samples presented in (a)–(f), fluorescent bands are active caspase-3. (i) FACS analysis of apoptotic MM1s cells (left) and KMS11 cells (right) labeled by 22. Cells were treated with etoposide for 18 hours, during the last three hours 22 was added for labeling of active caspase-3. Non-treated cells, black line, cells with etoposide and ZVAD-FMK, red line, cells with etoposide, blue line and cells without probe or etoposide, gray line. ANOVA test using Sidak's correction for multiple comparisons was applied, **** denotes adjusted *p* value < 0.001.

As probe **22** only becomes fluorescent after caspase-3 specific cleavage, we next examined the dynamics of caspase-3 activity in apoptotic cells using microscopy. MM1s cells were treated with etoposide and probe **22** was added for the last three hours of treatment before fluorescent images were collected with a confocal microscope. Legumain and caspase inhibitors were included as controls (Fig. 3a–f). Images were then quantified for percent of Cy5 fluorescent cells per field (Fig. 3g). We detected no decrease in the fluorescent signal with the legumain inhibitor while ZVAD-FMK (caspase inhibitor) significantly reduced the signal. In order to confirm that the observed fluorescent signal corresponded to labeling of active caspase-3, we analyzed the same samples by SDS-PAGE (Fig. 3h). The microscopy images closely matched the specific signal form the gel analysis, thereby showing specific labeling of cleaved caspase-3 by **22**.

Fluorescence-Activated Cell Sorting (FACS) in combination with probe **22** was also used to analyze MM1s and KMS11 cells after etoposide treatment. In the MM1s cells Cy5 fluorescence increased from 23% (basal fluorescence) to 88% within 18 hours after apoptosis induction with etoposide. Addition of the inhibitor ZVAD-FMK reduced the fluorescence back to the basal level of 23.2%, thereby verifying the specificity of the signal for cleaved caspase-3 (Fig. 3i). In contrast, we did not detect any significant increase in fluorescence in the resistant KMS11 cells: the basal apoptosis levels were 15.2% and were induced to 20.4% by etoposide treatment. Moreover, ZVAD-FMK had no significant impact on the fluorescent signal of KMS11 cells (Fig. 3i).

### Real-time localization and kinetics studies of cleaved caspase-3 with probe **22**

**Fig. 4:**
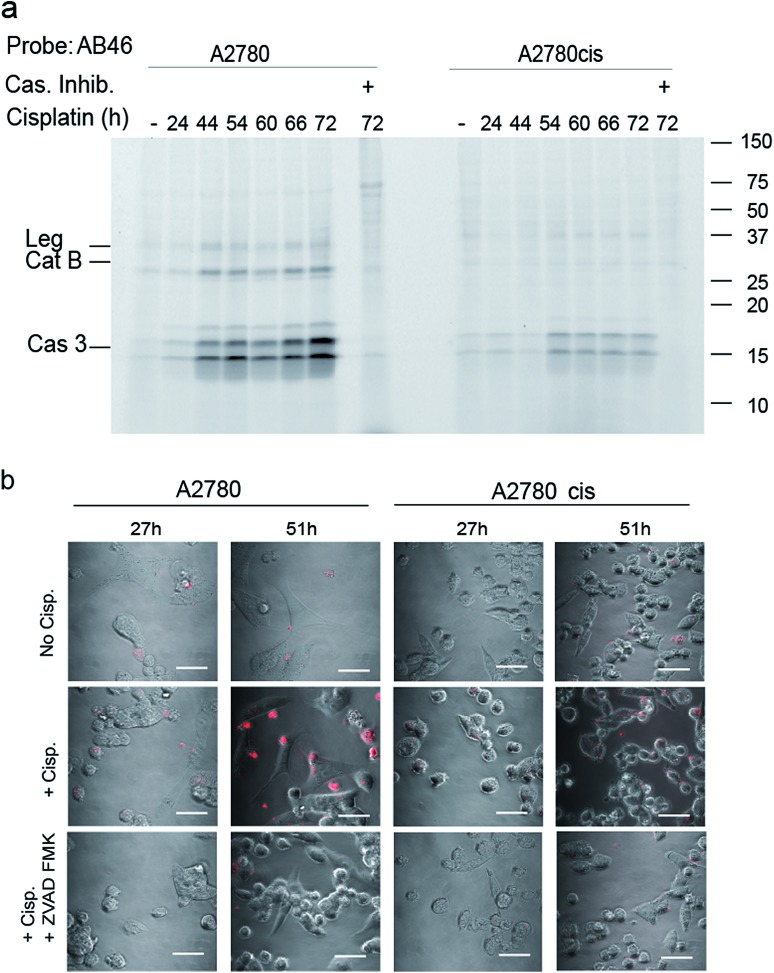
Caspase activity in A2780 and A2780cis cells. (a) Time-course activation of caspase-3 during apoptosis induced in A2780 and A2780cis cells. Cisplatin was added to the growth media for indicated times to intact cells one day after seeding, samples marked with (+) were treated with ZVAD-FMK one hour before probe treatment. All samples were labeled by AB46-Cy5 for 3.5 hours, lysed and equal protein was loaded on a 14% SDS-PAGE gel. Fluorescent bands were detected as described above. (b) Fluorescent microscopy of caspase-3 activity in A2780 and A2780cis cells. In order to visualize the localization of active caspase-3 during apoptosis, A2780 and A2780cis cells were treated with 5 μM cisplatin for the indicated times, qABP **22** was added 24 hours after cisplatin addition and fluorescence was visualized using confocal microscope between 27–72 hours after apoptosis induction, scale bar = 30 μm.

To determine the suitability of the qABP **22** for real-time imaging of active caspase-3 in apoptosis, we performed experiments with the adherent ovarian carcinoma cells A2780 and A2780cis which are sensitive and resistant to cisplatin treatment, respectively, a widely used anticancer chemotherapy.^31^ First, we performed a time course study of caspase-3 activation over 3 days of cisplatin-induced apoptosis. At various time points throughout the experiment, AB46-Cy5 was added to label active caspase-3. Cells were then collected, washed, lysed and an equal protein amount per lane was loaded and resolved by SDS-PAGE, followed by scanning for Cy5 fluorescence. We detected prolonged caspase-3 activation between 24 and 72 hours, with a maximum at 72 hours after treatment in A2780 cells (Fig. 4a). The specificity of the labeled bands was verified by competition with the inhibitor ZVAD-FMK. In A2780cis cells, however, the signal of cleaved caspase-3 was observed at a later time interval after cisplatin treatment (54 to 72 hours) and was significantly less intense.

To visualize the localization of active caspase-3 during cellular apoptosis, we used probe **22** and captured images of cisplatin-treated A2780 and A2780cis cells for 72 h with a confocal microscope (Fig. 4b). In A2780 cells, a fluorescent signal could be detected after 27 hours and 51 hours. The signal corresponded to active caspase-3 since ZVAD-FMK treatment extinguished the fluorescent signal. As expected, fluorescence intensity was weaker in the cisplatin resistant A2780cis cells, although it was also specific to cleaved caspase-3 as demonstrated by competition with ZVAD-FMK.

**Fig. 5:**
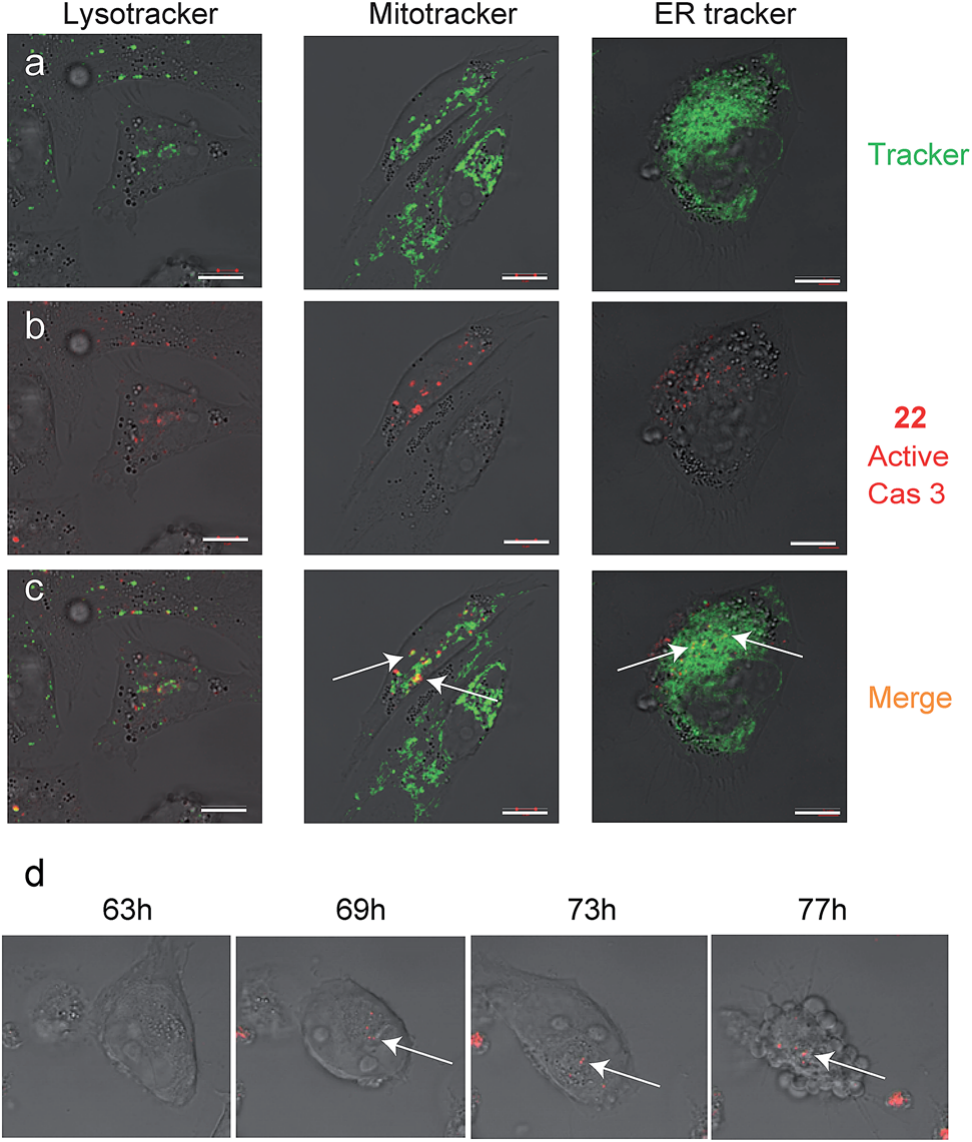
Caspase-3 activity localization studies in cellular compartments. Cultures of A2780 cells were grown in 8-well chambers. The cells were treated with cisplatin for 48 h then **22** was added to growth media for additional 24 h. Lysotracker, Mitotracker or ER-tracker was added and cells were imaged with an inverted fluorescent microscope. (a) Green fluorescence cell tracker; (b) red fluorescence, caspase-3 activity from **22**; (c) yellow color, overlap of red and green signal. Active caspase-3 was detected in mitochondria and ER compartments. Scale bar 10 μm. (d) A2780 cells were induced to undergo apoptosis with cisplatin for 30 hours then **22** was added and pictures were acquired every 15 minutes over the next 48 hours to generate a time lapse movie (ESI Movie 1†). Pictures extracted from the time laps movie are presented. The indicated time corresponds to the start of cisplatin treatment. Red indicates Cy5 fluorescence from **22** seen overlaid bright field images.

Since we detected defined punctate patterns rather than diffuse signals of active caspase-3, we set out to investigate the localization of active caspase-3 during the apoptosis process in higher resolution. We used **22** as a real-time imaging probe and visualized live intact cells undergoing apoptosis in the presence of three markers for cellular compartments using confocal microscopy. A2780 cells were placed in a “live imaging” incubation chamber and were treated with cisplatin for 72 hours. The qABP **22** was added after 24 h together with three different organelle-specific trackers, *i.e.* Lysotracker (an acidotropic marker) for localizing lysosomes, Mitotracker for mitochondria, and ER-Tracker to visualize the endoplasmic reticulum (ER) (Fig. 5a–c). We detected active caspase-3 in the cytoplasm and co-localized with mitochondria and the endoplasmic reticulum.

To visualize the kinetics of caspase-3 activation during the apoptotic process, a real time movie of A2780 cells treated with cisplatin was captured in a time lapse movie over 78 hours (ESI Movie 1†). Snapshots from representative periods are presented in Fig. 5d. The movie reveals defined fluorescent punctate representing caspase activity throughout the cellular compartments. Of note, we observed some variability of caspase activation during the time course in some cells, most likely due to their non-synchronicity.

**Fig. 6:**
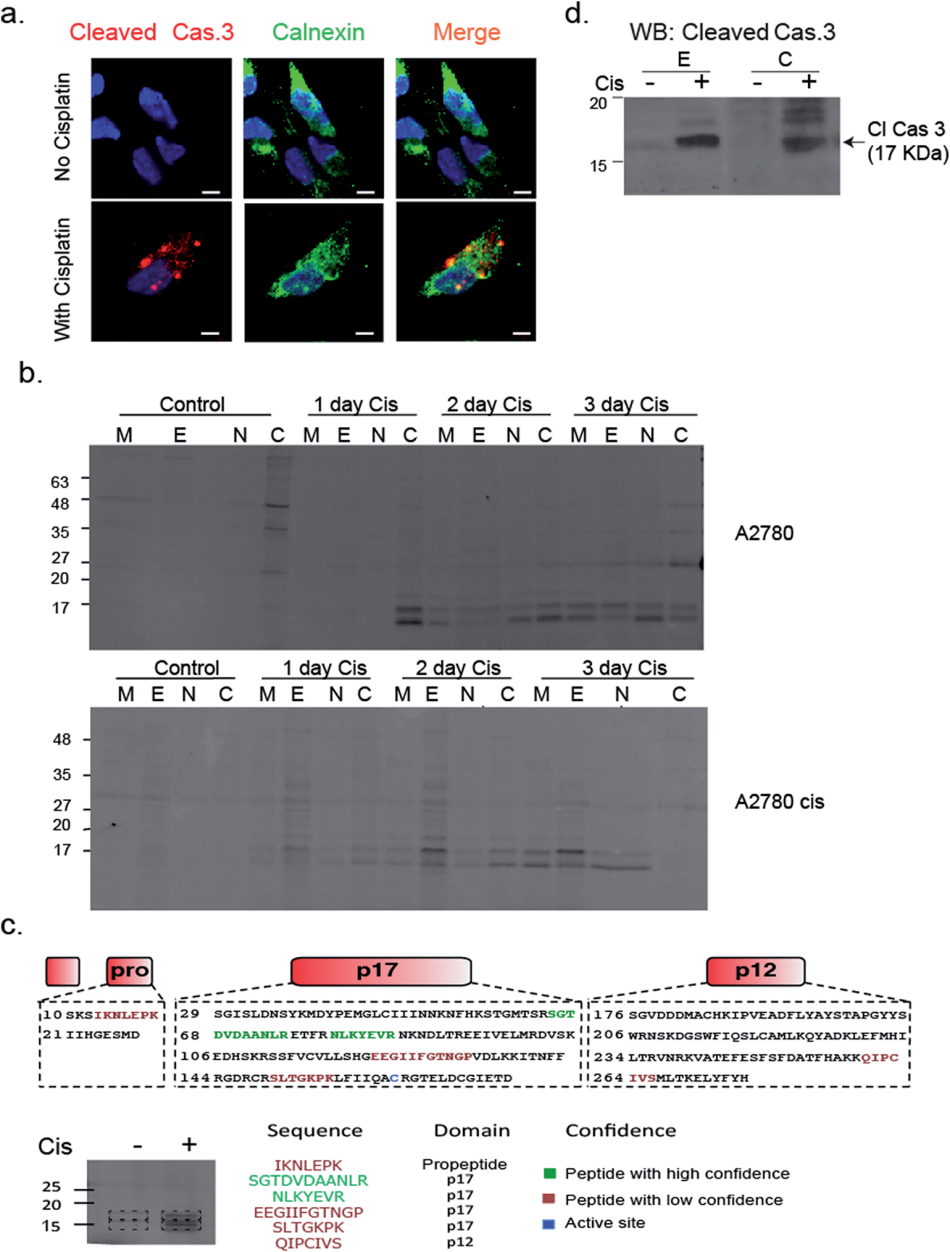
Caspase 3 activity is localized in the ER. (a) A2780 cells were treated with cisplatin for 50 hours; cells were fixed, permeabilized, blocked and treated with calnexin and caspase-3 antibodies. Cells were imaged with a Zeiss fluorescent microscope. Red, cleaved caspase-3; green, calnexin; blue DAPI, scale-bar is 5 μm. (b) A2780 and A2780cis cells were treated with cisplatin for indicated times. Probe **22** was added to cell lysate and cell fractionation was performed. Equal protein amounts from all fractions were spread by a 14% SDS-PAGE, in-gel fluorescence shows caspase bands in the ER fraction. (c) Identification of caspase-3 in ER fractions. The ER fractions from cisplatin treated and untreated A2780 cells were labeled with **22** and subjected to immuno-precipitation with an antibody against Cy5. Labeled bands and corresponding areas from untreated samples were cut out and subjected to mass spectrometry. Several caspase-3 peptides were identified from the P17 subunit, two in high confidence shown in green. (d) Western blot of ER and cytosolic fractions from A2780 cells that were cisplatin treated or untreated for 3 days with a cleaved caspase-3 antibody.

To validate that active caspase-3 is indeed also localized to the ER, we performed a similar experiment in which A2780 cells were induced to undergo apoptosis by cisplatin treatment and analyzed them by fluorescence microscopy after addition of a cleaved caspase-3-specific antibody and an ER-marking calnexin antibody. Images show a clear co-localization between the cleaved caspase-3 antibody and the calnexin marked ER (Fig. 6a). In addition, we detected cleaved caspase-3 in the nucleus of many cells undergoing cisplatin-mediated apoptosis (ESI Fig. 4†).

To confirm and further study the kinetics of caspase-3 activity appearance in cellular compartments, we performed cell fractionation experiments with cells undergoing apoptosis over the course of 72 hours. A2780 or A2780cis cells were treated with cisplatin and probe **22** was added after cell lysis. The mitochondria, ER, nuclear and cytosolic fractions were then collected. We could for the first time detect caspase-3 activity in the ER fraction after two days of treatment in A2780 cells. From this time-point on, the intensity continuously increased over time. Interestingly, we found in A2780cis cells lower and delayed caspase-3 activity levels but detected active caspase-3 in the ER already one day after treatment started. In both cell lines, caspase-3 activity was also detected in the mitochondria, cytosol and nucleus fractions, as expected from previous reports^32^–^34^ (Fig. 6b).

In order to confirm the quality of our fractionation method, proteins extracted from each fraction (*e.g.* mitochondria, endoplasmic reticulum, nucleus and cytosol) were digested and analyzed by mass spectrometry-based label-free quantification and sub-sequent analysis for pathway enrichment (ESI Fig. 5a†). As anticipated, pathways corresponding to metabolic activities such as glycolysis and oxidative phosphorylation were highly enriched in the cytoplasm and mitochondria fractions, while RNA processing and protein folding were enriched in the nucleus and endoplasmic reticulum, respectively. Interestingly, we detected a few inhibitors of apoptosis in the ER fraction, such as cell division cycle and apoptosis regulator 1 (CCAR1), and apoptosis inhibitor 5 (API5). A full list of proteins detected in each cell fraction is found in ESI Table 1†.

To corroborate that the labeled bands were indeed caspase-3, the labeled ER fractions were subjected to immuno-precipitation with an anti-Cy5 antibody followed by mass spectrometry analysis of the labeled bands. Six caspase-3 peptides were detected in the cisplatin treated samples while they were absent in the control samples. Two of the found sequences were high confidence tryptic peptides that originated from the caspase-3 p17 subunit, which enabled detection of caspase-3. In addition, two short tryptic peptides and two trypsin miscleavage peptides were detected with lower confidence, strengthening caspase-3 is the specific and only target of **22** in apoptotic cells (Fig. 6c). Finally, the ER fractions were subjected to western blot with an antibody against cleaved caspase-3, here again cleaved caspase-3 was detected in the ER fraction only after cisplatin treatment (Fig. 6d). In addition, to eliminate the possibility that the signal in the ER was generated by binding to caspase-7, a western blot with a caspase-7 antibody was performed with cellular fractions after cisplatin treatment. The cleaved caspase-7 was not detected in the ER fraction (ESI Fig. 6†).

## Discussion

While numerous tools to study caspase activity have been produced, here we show for the first time a specific quenched activity-based probe (qABP) to enable high-resolution imaging. We have developed a selective caspase-3 qABP **22** that covalently modifies active caspase-3 and lacks background fluorescence when free in solution while generating a fluorescent signal when it is bound to its enzyme target. We found **22** to have versatile applications including high-resolution real-time imaging, real-time monitoring of caspase-3 activation during apoptosis and various biochemical applications.

In contrast to activity-based probes for proteases such as the cathepsins or the proteasome for which highly selective probes are easily created,^25^,^35^ generating selective probes towards caspases had so far proven to be a significant challenge. We found that the reactivity of chemical probes to legumain and cathepsin B is directly affected by the interaction of the inhibitor with the enzyme's prime binding sites. Therefore, we undertook a rational approach to lower their affinity to these proteases. To this end, we modified our qABP at both the P2 position (non-prime site) and by appropriate positioning of a blackberry quencher to impair prime sites interactions. Importantly, these modifications did not reduce the potency towards caspase-3, since we included a glycine residue at the prime site, that is well accepted by caspase-3,^36^ leading to a selective caspase qABP.

The use of ABPs to detect caspase activity instead of antibodies for cleaved caspase-3 is of great importance since the cleaved “active” caspase-3 may be inhibited by an active site steric blockage that is induced by different endogenous inhibitors. In such cases the cleaved caspase-3 antibody might recognize a cleaved-inhibited form of caspase-3, while the ABP would be able to detect the “true” activity. Furthermore, although the lack of enzymatic amplification causes covalent modifiers to have weaker signals than substrate based reporters, they are still highly valuable since they can report on the exact location of the target and be applied for SDS-PAGE analysis.

In this report we applied our newly developed probe to investigate caspase-3 localization during apoptosis in the ovarian carcinoma cell lines A2780 and A2780cis. In addition to the expected cytosolic activity several punctate stains appeared (Fig. 4b and 5). Using cellular tracker stains together with cellular fractionation we discovered that the activity in the organelles is located in the mitochondria and the ER. There have been numerous reports describing the localization of caspase-3 mainly as cytosolic and nuclear,^10^,^37^ a few reports on caspase-3 localized to the mitochondria^32^,^33^ and one report indicating active caspase-3 found in the ER of rabbit hippocampus upon aluminium-induced neurotoxicity.^38^ Furthermore, caspase-7 was reported to be localized in the ER following anti FAS treatment in mouse liver.^39^ Therefore, ER localization of active caspase-3 is supported by literature precedence but is now detected for the first time in cancer cells.

The ovarian carcinoma cell lines A2780/A2780cis cells exhibited differential activation of caspase-3 during apoptosis (Fig. 4). Caspase-3 activity was greatly reduced in A2780cis cells which might explain their resistance to cisplatin treatment. We anticipate that this reduction in caspase-3 activity originates from endogenous inhibitor binding. In fact, high basal levels of the caspase-3 inhibitor, XIAP, and other apoptosis inhibitors such as survivin, and C-IAP-1 in addition to the antiapoptotic protein BCL-2 were found independent of cisplatin treatment in these cells^40^ while the sensitive counterpart A2780 cells showed reduced levels of these proteins upon cisplatin treatment.

What is the physiological relevance of directing caspase-3 to the ER? We suggest two hypotheses: the cell tries to limit cellular destruction by positioning caspase-3 in the vicinity of ER-based apoptosis inhibitors, thereby preventing caspase-mediated cell death. Alternatively, caspase-3 is localized to the ER in order to dismantle this compartment as a part of the apoptosis process. As we show in Fig. 6b, we detected different caspase-3 kinetics in ER localization between the resistant A2780cis and sensitive A2780 cells in which the resistant cells show more rapid ER localization. Therefore, we favor the first hypothesis as shuttling or activating caspase-3 in the ER reduces its destructive capabilities in an attempt to diminish apoptosis. Nevertheless, both hypotheses require further investigations to elucidate the underlying molecular mechanisms.

There are a few published reagents for *in vivo* imaging of apoptosis which contain radiolabeled reporters like ^18^F tracers. These include the Aposense compounds^41^–^43^ or Isatin based compounds.^44^ Several chemical modifications of probe **22** would be necessary to enable detection of apoptosis *in vivo* since **22** includes an ester moiety that would be hydrolyzed by esterases found in the blood stream leading to probe destruction. Nevertheless, **22** is a unique reagent that could be widely used in cell cultures to investigate caspase activity and dynamics in the live cell. Taken together, we present probe **22** as a tool to investigate the changes in activity levels and localization of caspase-3. In our work we apply the probe to further investigate impaired caspase-3 activation in cells that are resistant to apoptosis by chemotherapy induction. We anticipate that a broader use of this novel chemical tool will allow gaining insight into caspase-3 regulation in numerous biological and disease based systems.

## Experimental

### General methods

The reagents were purchased from commercial suppliers and used without further purifications. All solvents were HPLC grade. All water-sensitive reactions were performed in anhydrous solvents under a positive pressure of argon. Cy5 succinimidyl ester (SE) was purchased from GE Healthcare. QSY 21 SE was bought from Molecular probes, Invitrogen and BBQ SE was acquired from Berry & Associates. Reactions were characterized by liquid chromatography-mass spectrometry (LC-MS) (Thermo Scientific MSQ-Plus attached to an Accela UPLC system) using reverse-phase chromatography with a water/acetonitrile gradient. For preparative purification of the compounds reverse-phase HPLC was conducted with a Chromeleon (Dionex) system using C18 or C4 columns. Fluorescent gels were scanned with a Typhoon FLA 9500 flatbed laser scanner (GE Healthcare Bio-Sciences AB, Uppsala, Sweden).

### Probe synthesis

The synthesis of compounds **1–19** is described in ESI Scheme 1 and Table 1 and in detail in the ESI.† The synthesis of qABP **21** and **22** is shown in Scheme 1.

### Competition assay in RAW cell lysate

Legumain inhibitory potency of probes was determined in RAW cell lysate using phosphate citrate buffer, pH 5.3, 5 mM DTT and 25 μg total protein per sample. The lysate was incubated with increasing concentrations of the corresponding probe for 1 h at 37 °C (with or without pretreatment of an inhibitor, AB46 peptide) followed by labeling with **4** for an additional 30 min. The reaction was stopped with 4× sample buffer (40% glycerol, 0.2% Tris/HCl pH 6.8, 20% β-mercaptoethanol, 12% SDS and 0.4 mg mL^–1^ bromophenol blue) and boiled. Lysates were separated by SDS-PAGE 12.5% acrylamide and visualized by fluorescence scanning of the gel with an Odyssey flatbed laser scanner (excitation/emission 680/700 nm).

### Competition assay of caspase-3

Recombinant human caspase-3 (19 μM, a gift from Guy Salvesen, Burnham Institute, La Jolla, CA) was diluted in reaction buffer (100 mM Tris pH 7.4, 0.1% CHAPS, 10% sucrose and 4 mM DTT) to 100 nM concentration. Increasing probe concentrations were added to caspase-3 at 37 °C for 1 h (with or without pretreatment with AB46 peptide),^18^**4** was added and the resulting solution was mixed for additional 30 min. The reaction was stopped as described above. Samples were separated on a 14% SDS PAGE and gels were scanned for fluorescence by an Odyssey flatbed laser scanner, excitation/emission 680/700 nm.

### Direct labeling of recombinant enzyme

Enzymes were incubated with increasing probe concentrations, with or without pretreatment with an inhibitor (AB46 peptide, GB111-NH_2_ or compound **5**, which bind to caspase 3, cathepsin B and legumain, respectively). The free probe was separated from the enzyme-bound probe using gel electrophoresis and the gel was scanned for fluorescence to detect the amount of enzyme bound probe in a similar fashion to the indirect labeling experiments.

### Cell cultures

MM1s and KMS11 multiple myeloma cells were cultured in RPMI-1640 medium (GIBCO) supplemented with 10% FBS, 1% of glutamine, 1% sodium pyruvate, 100 units mL^–1^ penicillin and 100 mg mL^–1^ streptomycin at 37 °C under 5% of CO_2_.

A2780 and A2780cis ovarian carcinoma cells were cultured in RPMI medium (GIBCO) adjusted to pH 6 supplemented with 10% FBS, 50 μg mL^–1^ of gentamicin at 37 °C under 5% of CO_2_.

### Probes penetration and labeling in apoptotic cells

MM1s and KMS11 cells were seeded in a 24 wells plate in varying density 2–4 × 10^6^ cells per well. Cells were induced to undergo apoptosis by addition of 75 μM of etoposide for 4–24 h. Cathepsin B inhibitor (5 μM of GB111-NH_2_) or caspase inhibitor (10 μM of AB46 peptide) or vehicle (DMSO) were added. Probes **17–22** were then added to the growth media for an additional 3 h unless otherwise noted. The final DMSO concentration was kept at 0.1% in all wells. Cells were washed once with PBS, lysed by incubation in hypotonic lysis buffer (10 mM PIPES, 10 mM KCl, 5 mM MgCl_2_, 2 mM EDTA, 1% NP40 and 4 mM DTT) for 10 min on ice followed by transferring through a 30G syringe. The lysates were frozen and thawed three times and finally supernatants were collected after a 20 min spin-down at 4 °C. The protein concentration was quantified by a Bradford assay. 30–100 μg total protein was separated by a 14% SDS PAGE and the gel was scanned for fluorescence by an Odyssey laser scanner (680 nm excitation/700 nm emission).

In experiments with A2780 or A2780cis cell cultures, cells were seeded 1.5 × 10^6^ cells per well 24 h prior to apoptosis induction with 5 μM of cisplatin for 24–72 h and lysates were prepared as described above.

### FACS studies

MM1s and KMS11 cells were seeded in a 24 wells plate in density 1 × 10^6^ per well and were treated with DMSO vehicle or induced to undergo apoptosis by addition of 75 μM etoposide for 18 h. The caspase inhibitor (ZVAD-FMK, 50 μM) was added to selected wells. After 15 h, probe **22** (1 μM) was added to the growth media for an additional 3 h, the final DMSO concentration was kept at 0.1% in all wells. Cells were then washed once with PBS and re-suspended in RPMI without phenol red. Flow cytometry was performed to determine the labeling of cleaved caspase-3 using a LSRII flow cytometer (BD Biosciences). The resulting data were analyzed with FACS express software (BD, BD Biosciences).

### Live cells imaging

A2780 and A2780cis cells were seeded at a density of 100 000 cells per well in an 8-well coverslip chamber (Nunc Lab-Tek). One day later, cells were treated with cisplatin (5 μM) for 24 h. The media was replaced with RPMI without phenol red but still containing cisplatin (5 μM). DMSO or 1 μM of probe **22** was added to the wells for additional 3 h of incubation (controls in which ZVAD-FMK 100 μM was added before treatment were applied). The fluorescent signal of cleaved caspase-3 (excitation/emission Cy5 filter) was captured using a confocal microscope (Olympus FV10i inverted microscope, Tokyo Japan, equipped with a ×60 Oil DIC lens) over 72 h and analyzed using FLUOVIEW software.

### Co-localization studies

A2780 and A2780cis cells were seeded in an 8-well coverslip chamber (Lab-Tek) at a density of 100 000 cells per well and grown at 37 °C under 5% of CO_2_, 24 hours later, cells were treated with cisplatin (5 μM) for 24 h. The media was replaced with RPMI without phenol red, supplemented with 3% FCS and either DMSO or 1 μM of probe **22** was added to the wells. After 48 h, the different cellular trackers were added in order to perform co-localization studies of both the tracker and the cleaved caspase-3 labeled by **22**. To reveal the location of mitochondria, probe-containing medium was withdrawn and medium with Mitotracker-orange (Life technologies, M7510, conc. 500 nM) was added 30 min prior to imaging, then washed for 5 min. To localize the endoplasmic reticulum, medium containing probe was withdrawn and ER-tracker solution (Life technologies, 500 nM in HBSS buffer) was added for the last 30 min prior to imaging. To reveal whether the signal is lysosomal, LysoTracker (Life technologies, 50 nM) was added minutes before imaging. Cells were live imaged using a Zeiss LSM 710 Axio Observer.Z1 with a 63×/1.4 Oil DIC M27 lens, in Cy5, FITC and M-cherry channels.

### Immuno-fluorescence co-localization studies

A2780 cells were seeded at a density of 15–30 000 cells per well in an 8-well coverslip chamber (Nunc Lab-Tek) and incubated at 37 °C under 5% of CO_2_. On the next day, cells were treated with cisplatin (5 μM) for 50 h. The media was then withdrawn and the cells were fixed using 4% paraformaldehyde. Cells were permeabilized using 0.1% Triton-X for 15 min, washed and incubated with 2% BSA for 20 min at RT. Cleaved caspase-3 Asp175 (Cell signaling #9664) staining was done using a 1 : 100 dilution of Cas-Block solution (Life Technologies) overnight at 4 °C. Simultaneously, calnexin (goat monoclonal antibody, cell signaling) staining was done using a 1 : 200 dilution of Cas-Block solution overnight at 4 °C. Cells were washed and incubated with an anti-rabbit secondary antibody conjugated to Cy5 and an anti-goat secondary antibody conjugated to Cy3. Cells were imaged with a confocal microscope (Zeiss LSM 710 Axio Observer.Z1 with a 63×/1.4 Oil DIC M27 lens, in Cy5, Dapi and Dsred channels).

### Cell fractionation

Proteins from the nuclear, mitochondrial, cytosol and ER fractions were extracted as described previously.^38^ In brief, A2780 cells were trypsinized, washed with PBS and centrifuged for 5 min at 1200 rpm. Cells were gently homogenized through syringe needles in 2 volumes of cold suspension buffer (20 mM HEPES-KOH (pH 7.5), 250 mM sucrose, 10 mM KCl, 1.5 mM MgCl_2_, 1 mM EDTA). The homogenates were labeled with 1 μM probe **22** for 20 min at 37 °C.

After labeling, homogenates were first centrifuged at 750 × *g* at 4 °C for 10 min to isolate the nuclear fraction, and then at 8000 × *g* for 20 min at 4 °C to separate the mitochondrial from the cytosolic fraction. The 8000 × *g* pellets were re-suspended in cold buffer without sucrose and used as the mitochondrial fraction. The supernatant was further centrifuged at 100 000 × *g* for 60 min at 4 °C to separate the cytosolic from the ER fraction. Protein concentrations were determined with Bradford protein assay reagent (Sigma). Equal amount of proteins from the nuclear, mitochondrial, cytosolic and microsomal fractions were separated by SDS-PAGE (14% gel), and scanned for fluorescence by a Typhoon scanner FLA 9500 (excitation/emission 630/670 nm) (GE Healthcare Bio-Sciences AB, Uppsala, Sweden).

### Western blotting

Protein lysates or cellular fraction were separated on SDS-PAGE gels were transferred to PVDF membranes for immunoblotting. Membranes were probed with cleaved caspase-3 Asp175 (Cell signaling #9664) and calnexin (BD Biosciences #610523) antibodies and detected using the appropriate HRP-conjugated secondary antibody, followed by an ECL assay (Biological Industries, Kibbutz Beit Haemek, Israel). Visualization of the chemiluminescent protein bands was performed using the Bio-Rad ChemiDoc XRS chemiluminescence detection system.

### Immuno-precipitation

For each sample, 1 mg total protein was incubated overnight in rotation at 4 °C. 100 μL protein A/G (Santa Cruz, CA, USA #2003) was added to each sample for two hours in rotation at 4 °C. After washing, 25 μL 2× sample buffer was added to the beads and boiled for 10 min. Proteins were separated on a 14% SDS-PAGE gel.

### In gel digestion

Eluted proteins were resolved on a 12.5% SDS-PAGE and visualized *in situ* by scanning fluorescence with a Typhoon FLA 9500 fluorescence scanner. Gel regions containing labelled proteins were then excised using the Blind-Cut method.^45^ A printout of fluorescently labeled proteins was placed below the gel and regions corresponding to labeled proteins were cut out using a razor blade as described in reference 46. Proteins were in gel reduced with 2.8 mM DTT (60 °C for 30 min) and alkylated with 8.8 mM iodoacetamide in the dark at room temperature for 30 min. Protein digestion was carried out using modified trypsin (Promega) at a 1 : 10 (w/w) enzyme-to-substrate ratio in the presence of 10% acetonitrile in 10 mM ammonium bicarbonate. The reaction was carried out at 37 °C overnight and tryptic digests were resolved by reverse-phase silica capillaries (J&W). Peptides were separated by an acetonitrile water gradient including 0.1% formic acid. Mass spectrometry analysis was performed on a Q-Exactive plus (QE, Thermo) in positive mode. A full MS scan was followed by High energy Collision Dissociation (HCD) of the 10 most intense ions selected from the first (precursor) MS scan. The MS^2^ spectra were uploaded by Proteome Discoverer 1.4 software to Sequest (Thermo) and Mascot (Matrix Science) search engines and searched against the UniprotKB human database.

### In solution digestion

Proteins from cellular compartments fractionation (10 μg) were reduced with 10 mM DTT in 8 M urea for 1 hour followed by alkylation with 50 mM iodoacetamide for another 30 min in the dark. Protein digestions were then carried out with LysC (Wako-chemicals) at 1 : 100 (w/w) enzyme-substrate ratio for 3 hours at 37 °C after which the urea concentration was reduced to 1.6 M with 25 mM ammonium bicarbonate and trypsin (Promega) was added at 1 : 50 (w/w) enzyme–substrate ratio for another 16 hours at 37 °C.^47^ Tryptic peptides were de-salted on homemade C_18_-Empore (Sigma-Aldrich) StageTips.^48^ Peptides were then taken up in 0.1% formic acid and analyzed by LC-MS/MS.

### LC-MS/MS

Peptides from the in-solution digests were analyzed on an Orbitrap Elite instrument (Thermo)^49^ that was coupled to an EASYnLC-1000 (Thermo) liquid chromatography (LC) system. The LC was operated in two-column mode. A 3.5 cm pre-column (100 μm × 3.5 cm, fused silica capillary with home-made integrated frit) packed with Reprosil-Pur 120 C18-AQ 3 μm resin (Dr Maisch) was placed in front of the analytical column (75 μm × 23 cm), fused silica capillary with integrated pico frit emitter; New Objectives PF360-75-15-N-5) which was packed in-house with Reprosil-Pur 120 C18-AQ 3 μm resin (Dr Maisch). The analytical column was hooked up to a nanospray flex ion source (Thermo). The LC was equipped with two mobile phases: solvent A (0.1% formic acid, FA, in UPLC grade water) and solvent B (0.1% FA in acetonitrile, ACN). Peptides were delivered to the pre-column *via* the integrated autosampler at a flow rate of 2–5 μL min^–1^ in 100% solvent A. Peptides were subsequently resolved on the analytical column by running a 70 min gradient of solvent A and solvent B (start with 2% B; gradient 2% to 35% B for 60 min; gradient 35% to 100% B for 5 min; 100% B for 5 min) at a flow rate of 300 nL min^–1^.

The Orbitrap Elite mass spectrometer was operated using Xcalibur software (version 2.2 SP1.48). The mass spectrometer was set in the positive ion mode. Precursor ion scanning was performed in the Orbitrap analyser (FTMS) in the scan range of *m*/*z* 300–1300 and at a resolution of 120 000 with the internal lock mass option turned on (lock mass was 445.120025 *m*/*z*), polysiloxane.^50^ Product ion spectra were recorded in a data dependent fashion in the ion trap (ITMS) in a variable scan range and at a rapid scan rate. The ionization potential (spray voltage) was set to 1.6–2.0 kV. Peptides were analyzed using a repeating cycle consisting of a full precursor ion scan (1.0 × 10^6^ ions) followed by 15 product ion scans (1.0 × 10^4^ ions) where peptides are isolated based on their intensity in the full survey scan (threshold of 500 counts) for tandem mass spectrum (MS^2^) generation that permits peptide sequencing and identification. CID collision energy was set to 35% for the generation of MS^2^ spectra. During MS^2^ data acquisition dynamic ion exclusion was set to 120 seconds with a maximum list of excluded ions consisting of 500 members and a repeat count of one. Ion injection time prediction, preview mode for the FTMS, monoisotopic precursor selection and charge state screening were enabled. Only charge states bigger than 1 were considered for fragmentation.

### Peptide and protein identification

The recorded RAW files were searched against the UniprotKB human database using the Andromeda search engine as integrated in MaxQuant (version 1.5.2.8). Andromeda searches allowed oxidation of methionine residues (16 Da) and N-terminal acetylation (42 Da) as variable modification as well as carbamidomethylation on cysteine (57 Da) as a static modification. All other settings for MaxQuant were the default settings. Most importantly the FDR for protein identification was set to 0.01 and the label-free quantification (LFQ) and match between runs options were enabled.

### Data processing and statistical analysis

Data analysis was done in Perseus (version 1.5.0.9) and performed on log 2 scaled normalised label free quantification (LFQ) values. Proteins were excluded from the analysis if they were either identified only by their variable modifications, identified using the reverse database or classified as a potential contaminant. Three replicates of each cellular fraction were grouped and valid values required at least two numeric values in at least one group. Missing values were replaced from the normal distribution and Anova statistical analysis performed to identify differentially expressed proteins with FDR = 0.05 and *S*0 = 1. Fischer's exact test was used for analysis of annotation enrichments with Benjamini–Hochberg FDR of 0.02. For fluorescent microscopy studies ANOVA test using Sidak's correction for multiple comparisons was done using Graphpad Prism 6 Software San Diego.

## Conclusions

We have developed a specific quenched fluorescent activity-based probe that serves as a tool to investigate the changes in activity levels and localization of caspase-3. The application of our selective probe reveals that caspase-3 is active in the endoplasmic reticulum during apoptotic cell death of cancer cells.

## Supplementary Material

Supplementary movie

Supplementary information
