# Supplementary material for: A novel quenched fluorescent activity-based probe reveals caspase-3 activity in the endoplasmic reticulum during apoptosis
Source: Chem Sci. 2015 Nov 11;7(2):1322–37. doi: 10.1039/c5sc03207e (PMC5975724; doi:10.1039/c5sc03207e)
Supplement: Supplementary file 2 [file SC-007-C5SC03207E-s002.pdf]

Electronic Supplementary Material (ESI) for Chemical Science.
This journal is © The Royal Society of Chemistry 2015

# A Novel Quenched Fluorescent Activity-Based Probe Reveals Caspase-3 Activity in the Endoplasmic Reticulum During Apoptosis

Yulia Shaulov-Rotem, Emmanuelle Merquiol, Tommy Weiss-Sadan, Ofra Moshel, Seth Salpeter, Doron Shabat, Farnusch Kaschani, Markus Kaiser and Galia Blum*

## Supplementary data

### Supplementary Scheme 1.

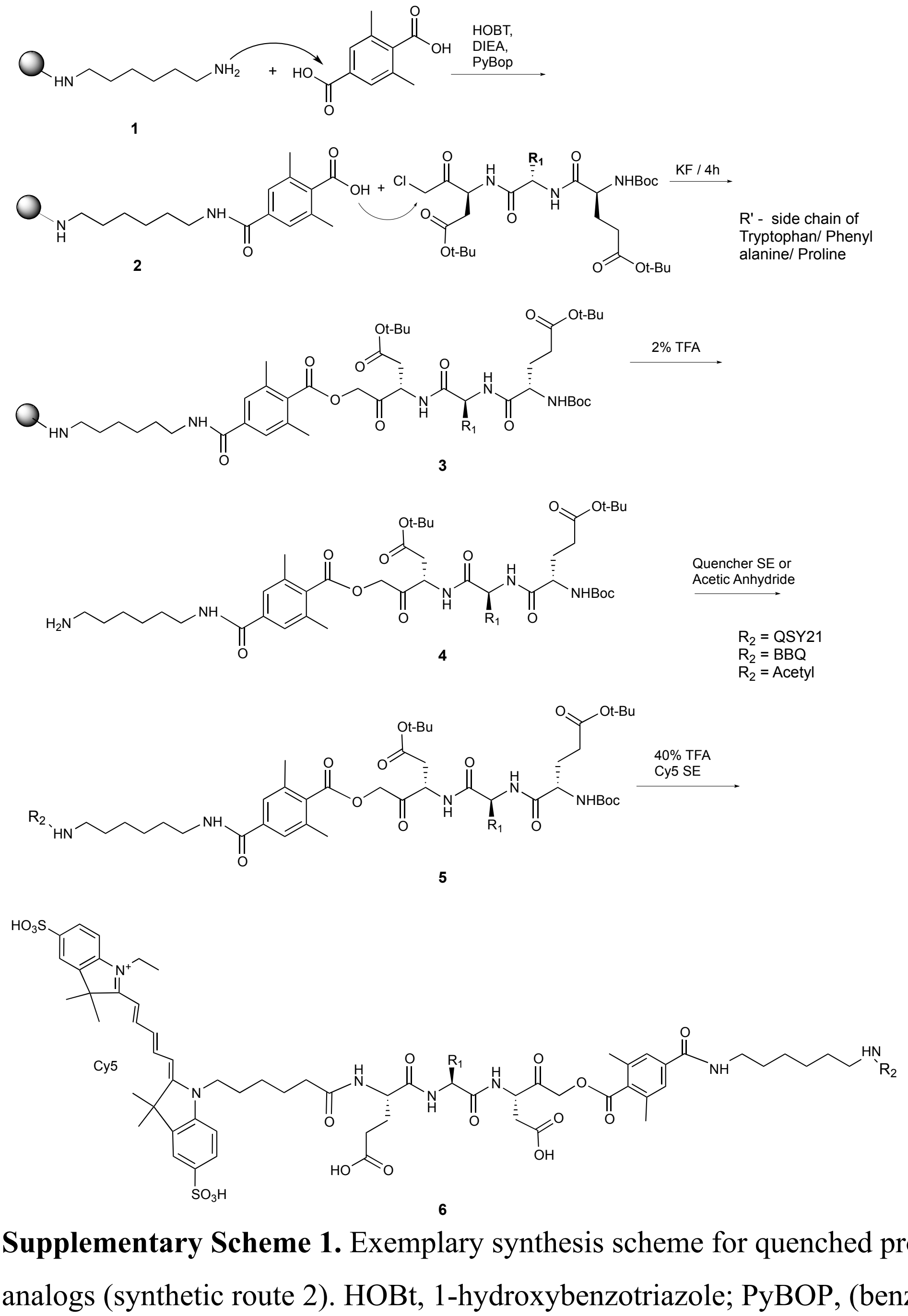


**Supplementary Scheme 1.** Exemplary synthesis scheme for quenched probes and non-quenched analogs (synthetic route 2). HOBt, 1-hydroxybenzotriazole; PyBOP, (benzotriazole-1-yl-oxy)tris(pyrrolidino) phosphonium hexafluorophosphate; DIEA, diisopropylethylamine; Boc, *t*-butyloxycarbonyl; SPPS, solid-phase peptide synthesis; TFA, trifluoroacetic acid, SE, succinimide ester.

**Supplementary Figure 1**.

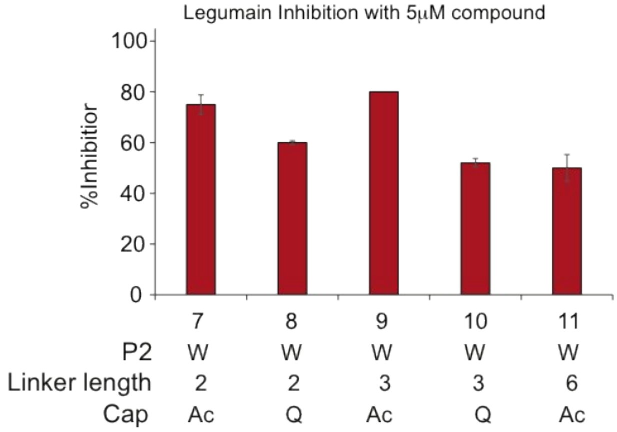


**Supplementary Figure 1**. Inhibition of legumain.

Inhibition of legumain in RAW cell lysates with 5 μM inhibitors described in Table 1. The lysate was incubated with increasing concentrations of the corresponding probe for 1 h at 37 °C followed by labeling with **4** for an additional 30 min. The reaction was stopped with 4X sample buffer (40% glycerol, 0.2% Tris/HCl pH 6.8, 20% β-mercaptoethanol, 12% SDS and 0.4 mg $mL^{-1}$ bromophenol blue) and boiled. Lysates were separated by SDS-PAGE 12.5% acrylamide and visualized by fluorescence scanning of the gel with an Odyssey flatbed laser scanner (excitation/emission 680/700 nm).

**Supplementary Figure 2.**

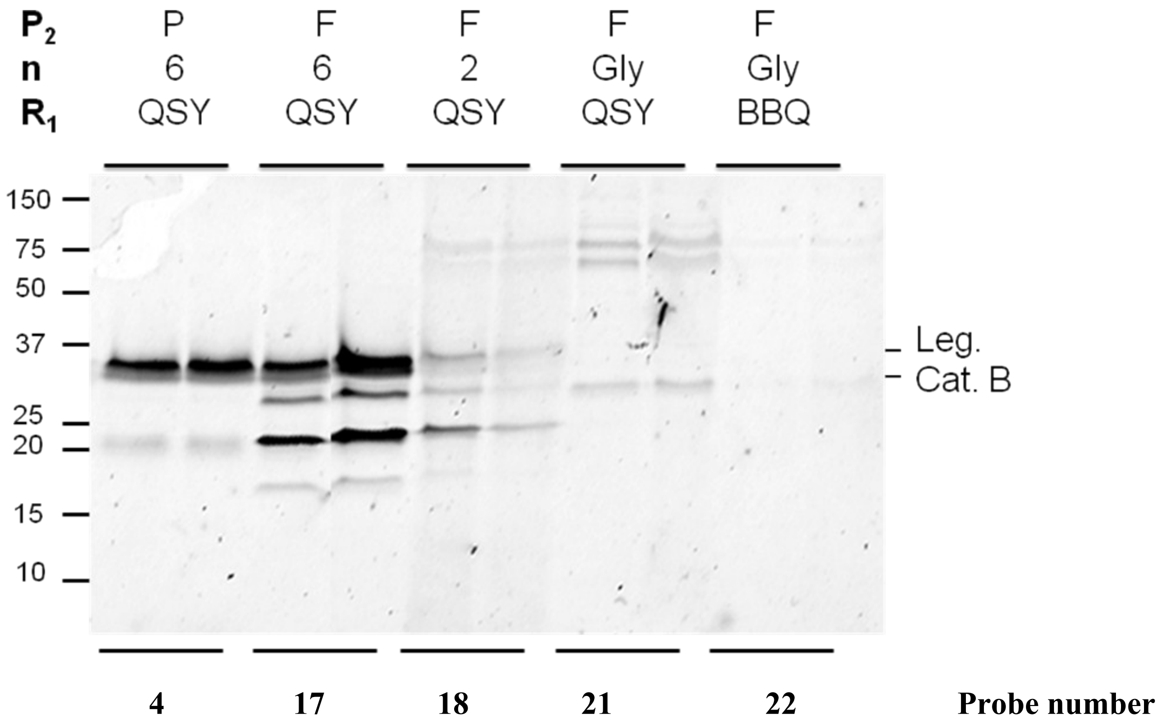

**Supplementary Figure 2.** Selected probes for in vitro labeling of legumain and cathepsin B in intact Colo 205 cells.

2 μM of probes were incubated with the cells for 4 h. The cells were collected, washed and lysed in hypotonic lysis buffer. Equal amount of proteins were loaded on 14% SDS PAGE, separated and scanned for Cy5 fluorescence with a Typhoon flatbed laser scanner (Ex/Em 635/665 nm).

**Supplementary Figure 3.**

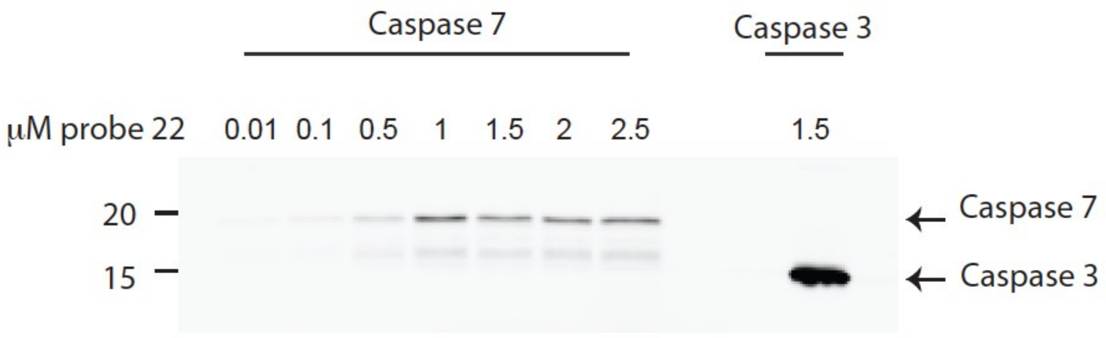


**Supplementary Figure 3.** Labeling of recombinant caspase-7 with qABP 22.

Probe 22 was incubated at various concentrations with recombinant caspase-7 or caspase-3 for one hour, the reaction was stopped, separated by SDS PAGE and the gel was scanned for fluorescence.

**Supplementary Figure 4.**

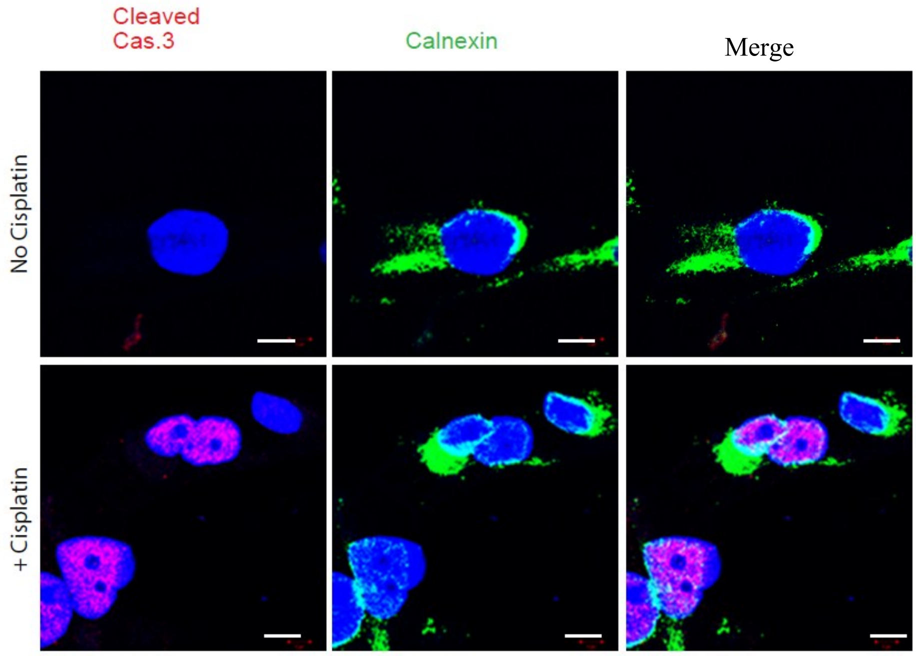


**Supplementary Figure 4.** Immunofluorescence colocalization studies for detecting cleaved Caspase-3 in the nucleus of A2780 cells.

A2780 cells were seeded in an 8-well coverslip chamber (Nunc) in RPMI supplemented with 10% FBS 50 µg mL$^{-1}$ of gentamicin and incubated at 37 °C under 5% of $CO_2$. After one day, 5 µM of cisplatin were added for 50 h, then the media was withdrawn and the cells were fixed using 4% paraformaldehyde. Cells were permeabilized using 0.1% Triton-X for 15 min and primary antibodies of cleaved caspase-3 (rabbit) and calnexin (goat) were added. Cells were washed and incubated with an anti-rabbit secondary antibody conjugated to Cy5 and an anti-goat secondary antibody conjugated to Cy3. Cells were photographed with a Zeiss LSM 710 Axio Observer.Z1 with a 63×/1.4 Oil DIC M27 lens, in Cy5, DAPI and DsRed channels. Scale bar is 5 µm.

**Supplementary Movie 1 legend.**

A2780 cells were seeded in an 8-well coverslip chamber in RPMI supplemented with 10% FBS and incubated at 37 °C under 5% of $CO_2$. After one day, cells were treated with 5 µM of cisplatin for 30 h. The medium was replaced with medium containing 1 µM of probe **22** and 5 µM cisplatin (with no phenol red at pH 6.7). The chamber was placed in a confocal microscope (a Zeiss LSM 710 Axio Observer.Z1 with a 63×/1.4 Oil DIC M27 lens, in Cy5 channel) under 5% of $CO_2$ and the wells were imaged every 15 min for 48 h.

**Supplementary Figure 5.**

**a.**

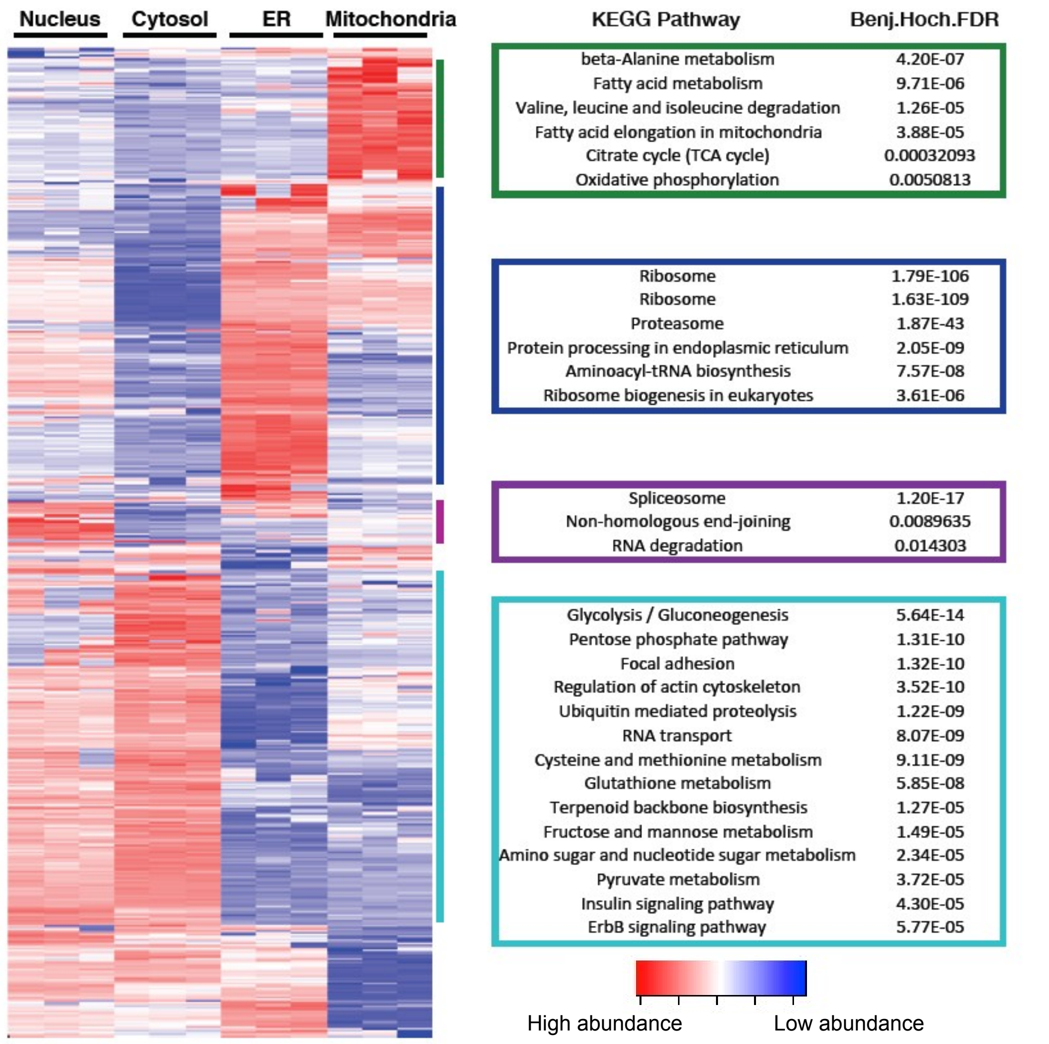


**b.**

**Blotting of cellular fractions with a calnexin antibody**

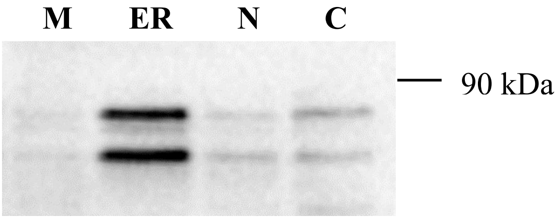


**Supplementary Figure 5.** Protein analysis of cellular fractions.

**a.** Cellular fractions were analyzed with mass spectrometry for their protein content. Identified proteins in each fraction were compared to biological processes using KEGG analysis. A heat map of proteins in the cellular fractions is presented, signaling pathways are color coded, the list is found on the right.

**b.** To verify the purity of the cellular fractions, a Western blot with an anti- calnexin antibody to detect calnexin (an ER marker) was performed, demonstrating calnexin enrichment in the ER fraction.

**Supplementary Figure 6.**

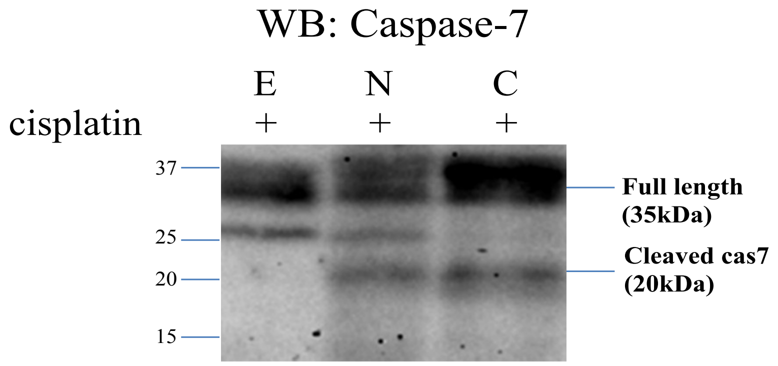


**Supplementary Figure 6.** Cleaved caspase -7 is not detected in the ER.

A2780 cells were treated with cisplatin for 3 days, cell were then lysed and cell fractionation was performed. Equal protein amounts from all fractions were spread by a 14% SDS-PAGE, that was transferred to a PVDF membrane and exposed to a caspase-7 antibody (Cell Signaling 9492). Cleaved caspase-7 (20kDa) was not detected in the ER fraction.

## Supplementary Methods

### Chemical synthesis and characterization of inhibitors and qABPs

The synthesis of the caspase inhibitors was essentially following the protocol previously reported in Blum et al. (Blum, von Degenfeld et al. 2007) (referred to as synthetic route 1); optimization of the protocol however allowed to increase the final yield of the qABPs synthesis, up to 30%.

#### Synthesis of the Acyloxy methylketone (AOMK) core fragment using a dimethyl terephthalic acid (DMTA) unit

An overview of the synthesis scheme is given in **Supplementary Scheme 1** referred to as synthetic Route 1. In brief, the synthesis initiates with the preparation of the required tripeptide sequence by solid phase peptide synthesis (SPPS). The peptide was cleaved using mild acidic conditions, thus maintaining the Boc and *t*-Bu protecting groups on the side chain residues. The protected tripeptide was then converted to a Boc-Glu (O*t*Bu)- (P2) Amino acid-Asp(O*t*Bu) chloromethyl ketone (CMK) which then was coupled to 2, 6-dimethyl-terrephthalic-acid. The AOMK was then removed from resin using mild acidic conditions, thereby maintaining the protecting groups on the side chain residues and allowing for a subsequent stepwise coupling of the quencher and the fluorophore. The product was purified by preparative HPLC, compound containing fractions were pooled and evaporated to dryness. Formation of ABPs from the AOMK building block was done as described below.

0.15 mmol of 2-Chlorotrytyl resin was shaken with 0.225 mmol Fmoc 1, 6-diaminohexane hydrochloride and 0.45 mmol DIEA in dry DCM for 1 h. 150 µl methanol was added to quench the resin and the overall resin loading was quantified to be 0.9 mmol/g. The Fmoc group was cleaved with 20% piperidine in DMF. For coupling the dimethyl-terephthalic acid, 1.5 eq (relative to the resin loading) of 2,6 dimethyl-terephthalic acid was preactivated with 1.5 eq HOBt, 1.7 eq PyBOP and 6 eq DIEA in DMF and then added to the resin bound free amine for 2 hrs. To form the AOMK warhead, 2 eq. of the tripeptide chloromethyl ketone premade as described in (Blum, von Degenfeld et al. 2007) and 10 eq potassium fluoride were added in dry DMF under argon for 2 hrs. The protected peptide was removed from resin in three 5 ml batches of

2% TFA in DCM. The cleavage solution was removed using vacuum after addition of toluene and the residue was purified by HPLC. For details see Supplementary Table 1.

**Synthesis of quenched activity based probe (qABP)**

1.1 eq of QSY21 SE (Invitrogen) or BlackBerry SE (Berry & Associates) or 10 eq of acetic anhydride were coupled to the free amine of the preformed AOMK and the resulting products were HPLC purified via C-18 RP preparative column to obtain the precursors of quenched ABP (qABP) and acetylated ABP, respectively (**Supplementary Scheme 1**). The protecting groups of aspartic acid and glutamic acid residues were removed using 40% TFA in dry DCM and the free amine terminus of each compound was coupled to Cy5 SE (GE Healthcare) to receive fluorescently labeled qABPs or acetylated ABPs, respectively. The ABP and its corresponding qABP were purified with a C-18 RP column in HPLC using a water/acetonitrile gradient (0.1 % TFA in DDW or 0.1 % TFA in acetonitrile). Product-containing factions were pooled, evaporated and lyophilized, thus receiving a blue powder. All final compounds were diluted in DMSO to receive a 50 mM stock solution that was used for biochemical studies.

**Acyloxy methylketone based on Glycine warhead formation, Scheme 1**

The synthesis of the tripeptide was done on solid support using 2-Chlorotrityl chloride resin (described above). After cleavage of the tripeptide from the solid support, it was converted into chloromethyl ketone and afterward reacted with *N*-(trityl)-glycine overnight at RT with potassium fluoride in DMF, to produce the AOMK. In the following step, the trityl protecting group was removed and the compound was reacted with one of the two quencher units, QSY21 SE or BBQ SE. After removal of the Boc protecting groups with 40% TFA, Cy5 was coupled to generate the desired qABPs. The final probes were purified by preparative HPLC and characterized using LCMS and High resolution-MS.

High-resolution mass spectrometry (HRMS) was performed using LTQ XL Orbitrap (Thermo Scientific). HRMS $[MH]^+$ calculated for compound **22** ($C_{86}H_{101}O_{22}N_{12}S_2$) is 1717.6589, found

1717.6583 ; the *m/z* for the $[M2H]^{2+}$ is 859.3331, found 859.3330. The difference between the calculated and the found mass was less than 1 ppm.

**LC-MS characterization conditions.**

A C-18 RP monolithic column or a C-4 RP column were used for characterization of crude and final products, with an injection volume of 5-15 µl of the product dissolved in methanol at a concentration of 1-10 µM. UV detection was performed at 215, 254 and 646 nm (for Cy5-labeled or quencher-labeled ABPs). MS probe conditions: ESI probe ionization voltage +75 V, probe temperature 300 °C.

**Preparative purification conditions.**

The crude products were injected in quantities of 2-50 mg via different loops (volume 0.1-1 ml) into the preparative column. Purifications were performed with C-18 RP or C-4 RP columns, using a gradient of water (DDW (0.1% of TFA)) and ACN (0.1% of TFA).

**LCMS Characterization**

**Compound 22.**

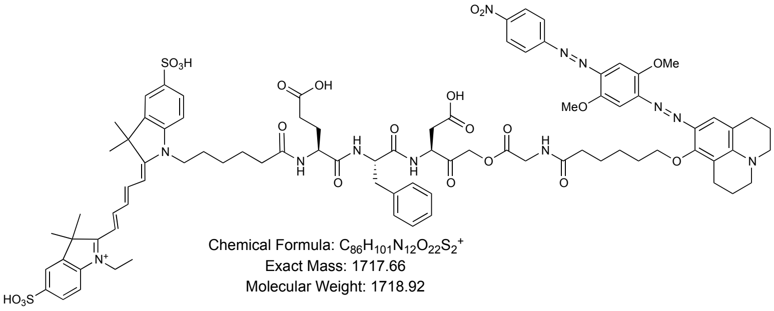


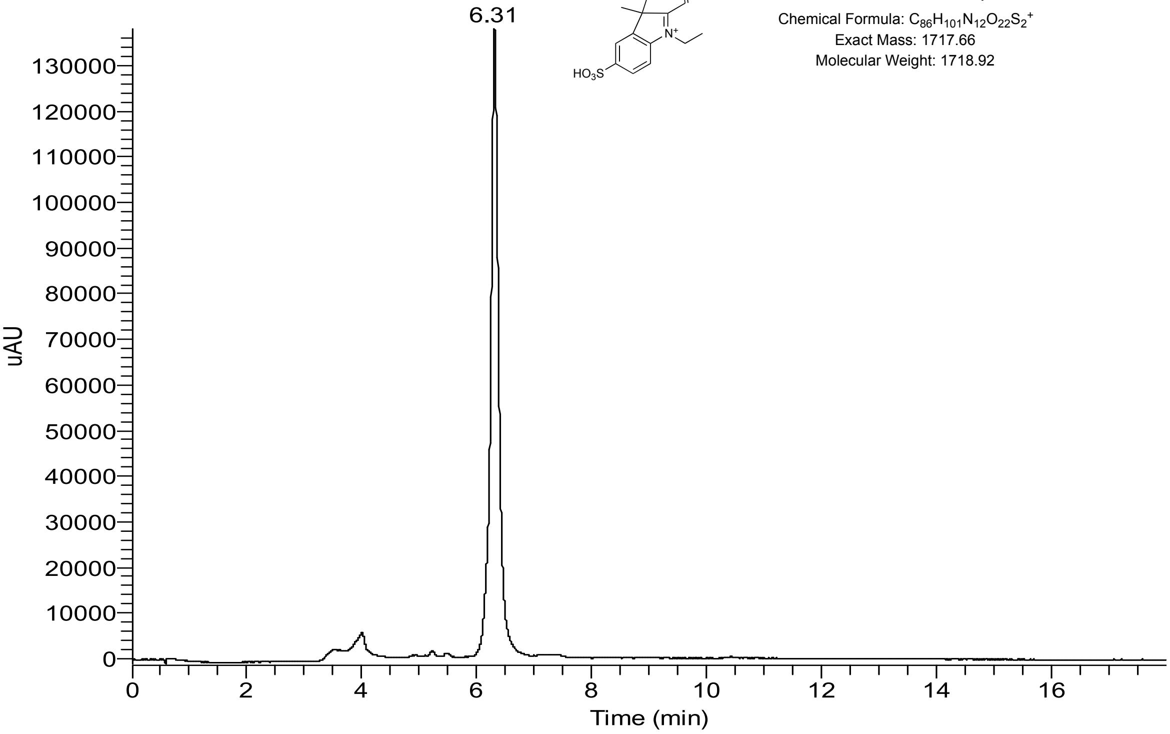


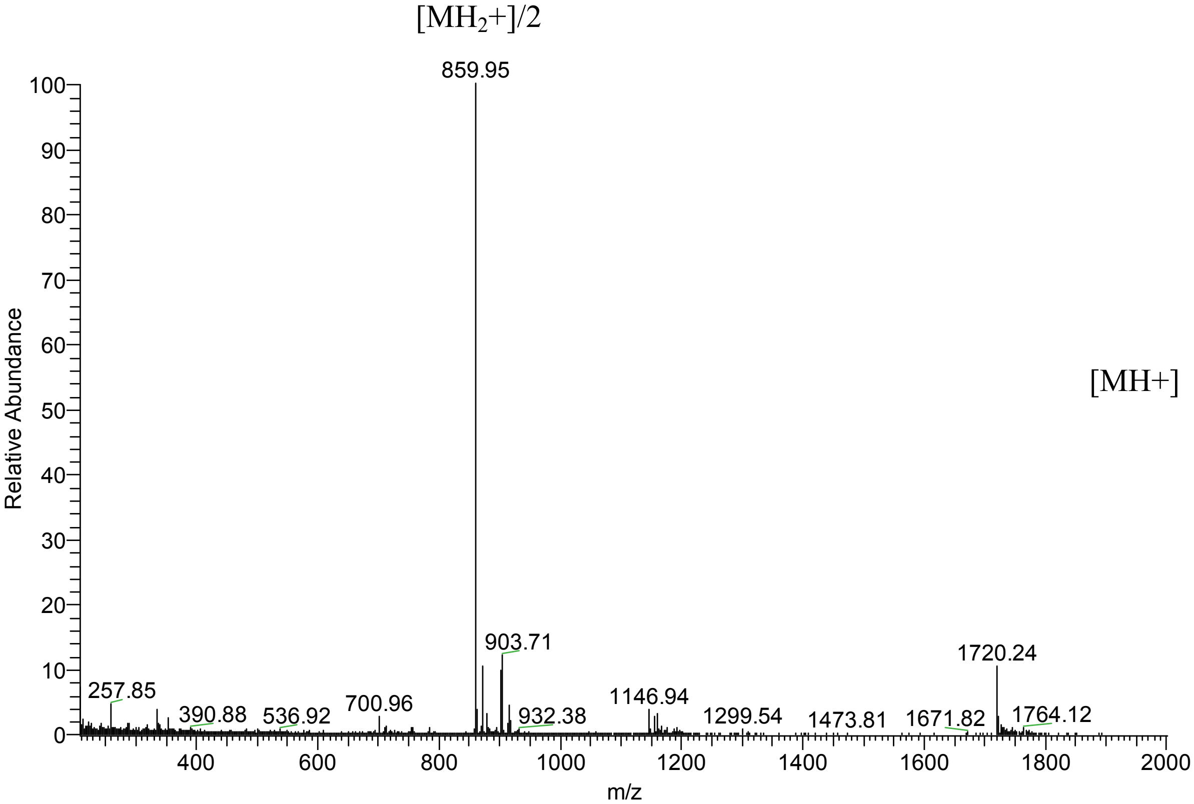

**Compound 1.**

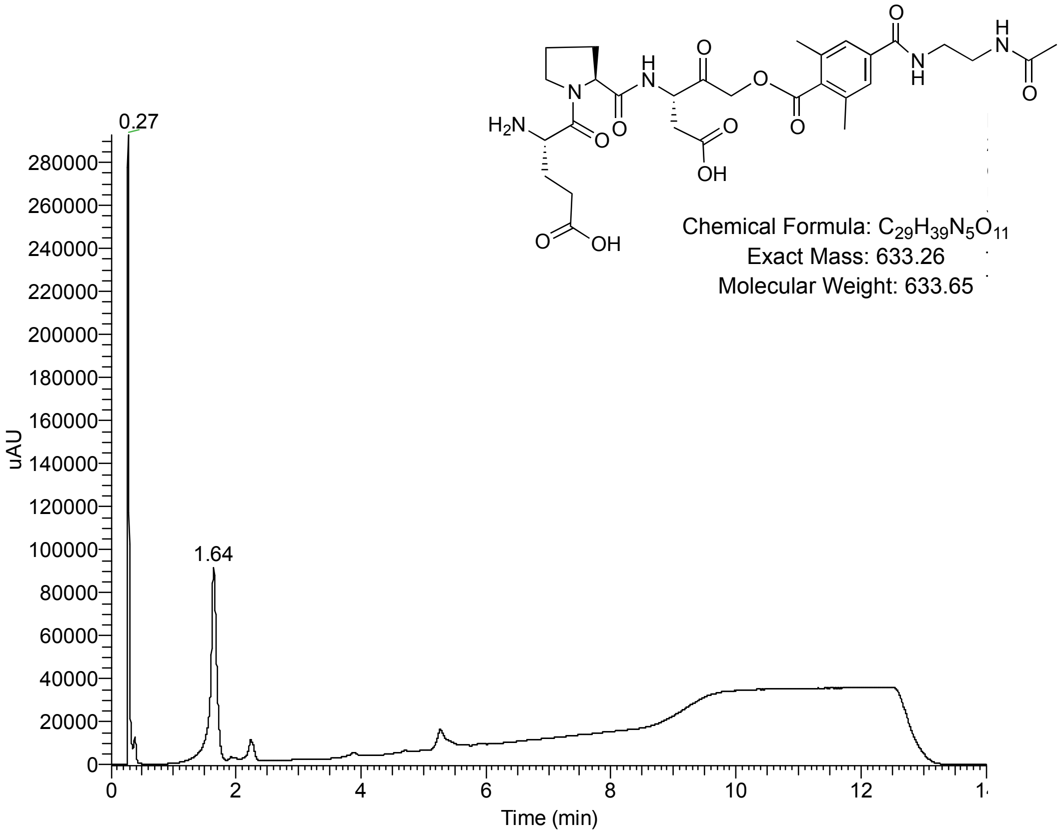
Chemical Formula: $C_{29}H_{39}N_5O_{11}$
Exact Mass: 633.26
Molecular Weight: 633.65
0.27
1.64
uAU
Time (min)

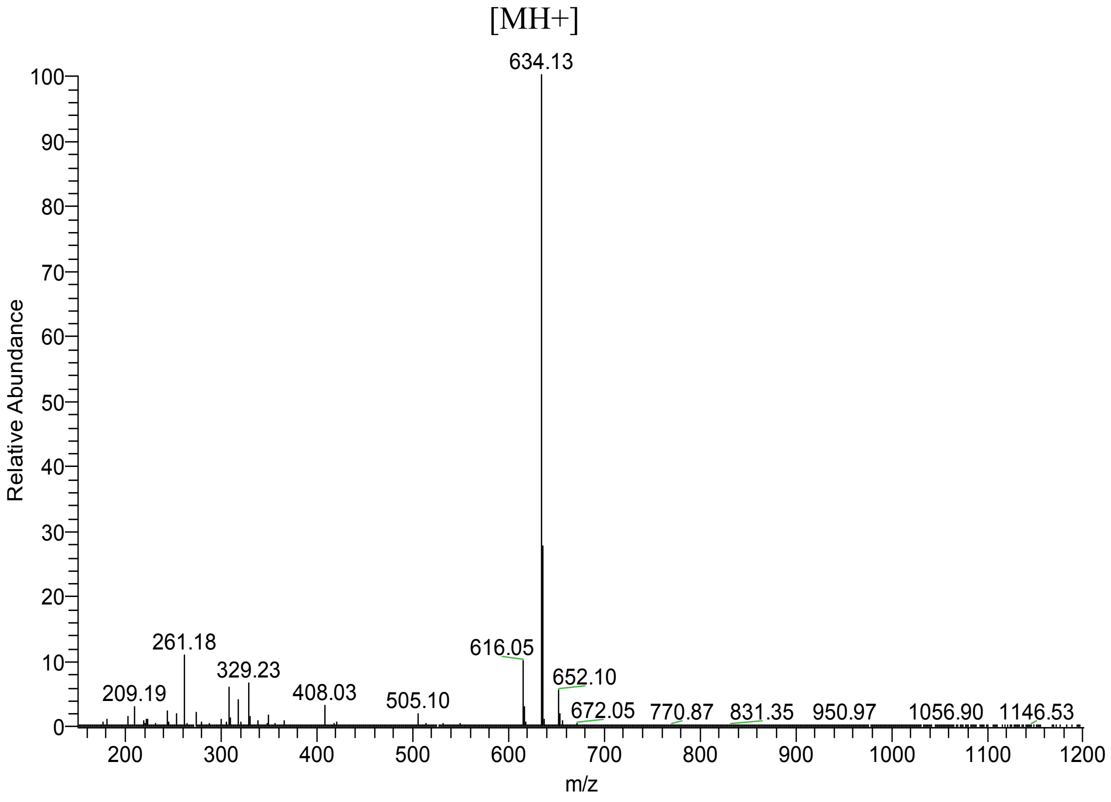
[MH+]
634.13
616.05
652.10
261.18
329.23
209.19
408.03
505.10
672.05
770.87
831.35
950.97
1056.90
1146.53
Relative Abundance
m/z

**Compound 2.**

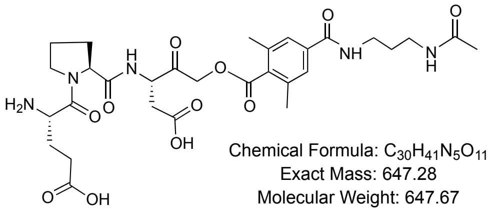
H2N
N
H
N
O
O
O
O
O
O
OH
O
OH
O
NH
O
N
H
O
Chemical Formula: C30H41N5O11
Exact Mass: 647.28
Molecular Weight: 647.67

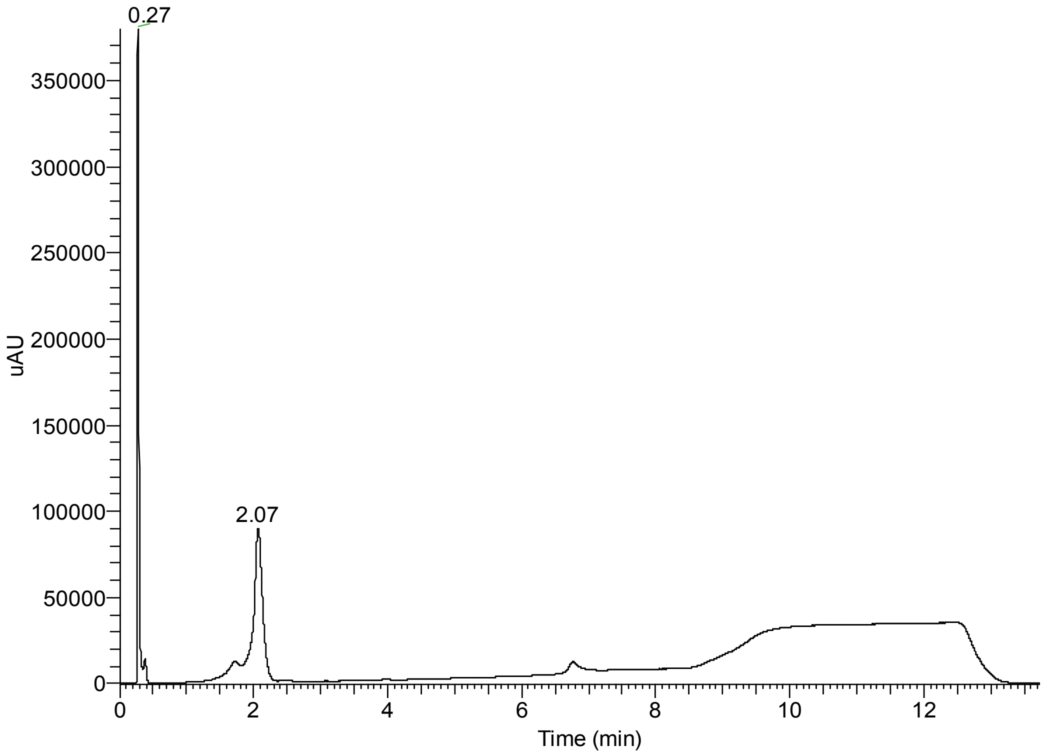
0.27
2.07
uAU
350000
300000
250000
200000
150000
100000
50000
0
0
2
4
6
8
10
12
Time (min)

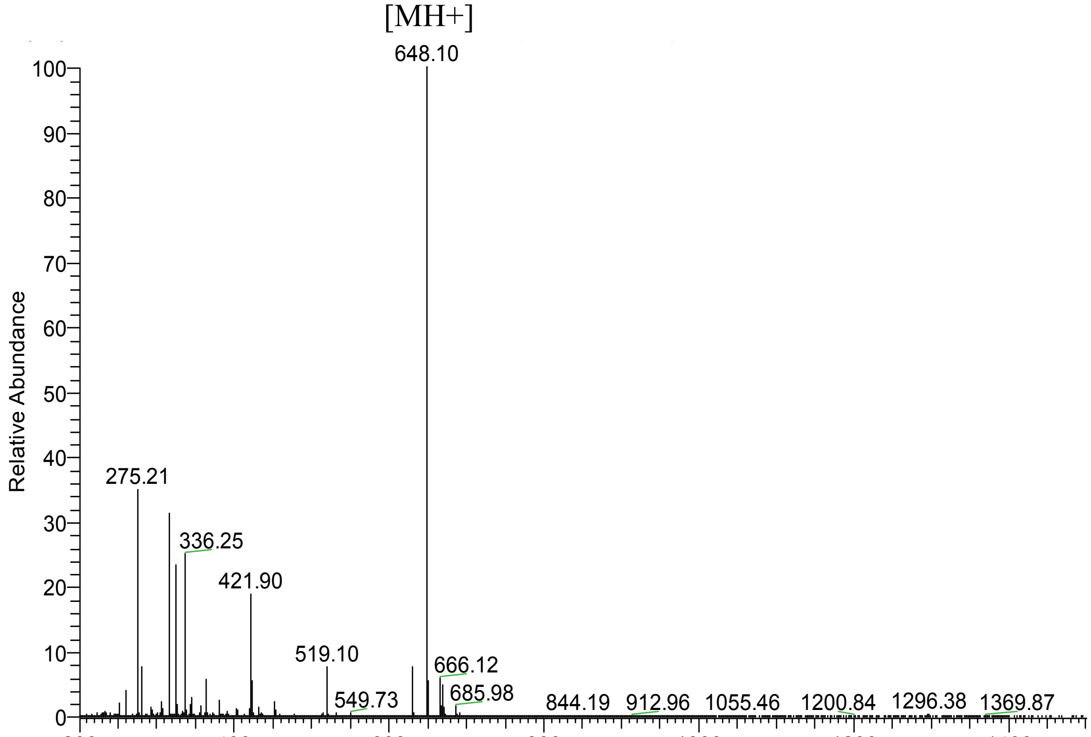
[MH+]
648.10
275.21
336.25
421.90
519.10
549.73
666.12
685.98
844.19
912.96
1055.46
1200.84
1296.38
1369.87
Relative Abundance
m/z
100
90
80
70
60
50
40
30
20
10
0
200
400
600
800
1000
1200
1400

**Compound 3.**

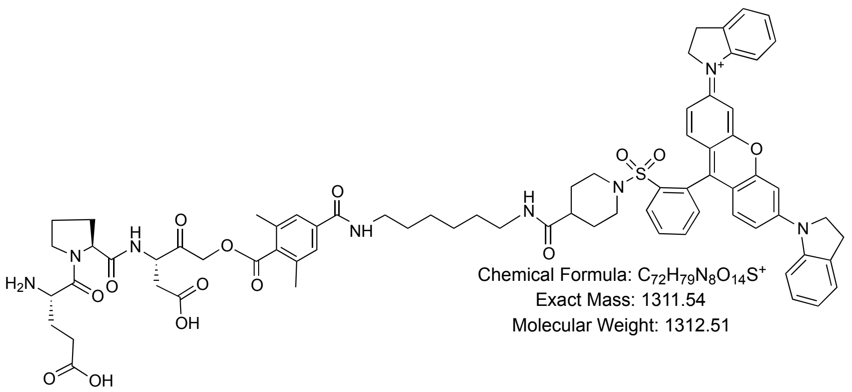
Chemical Formula: $C_{72}H_{79}N_8O_{14}S^+$
Exact Mass: 1311.54
Molecular Weight: 1312.51

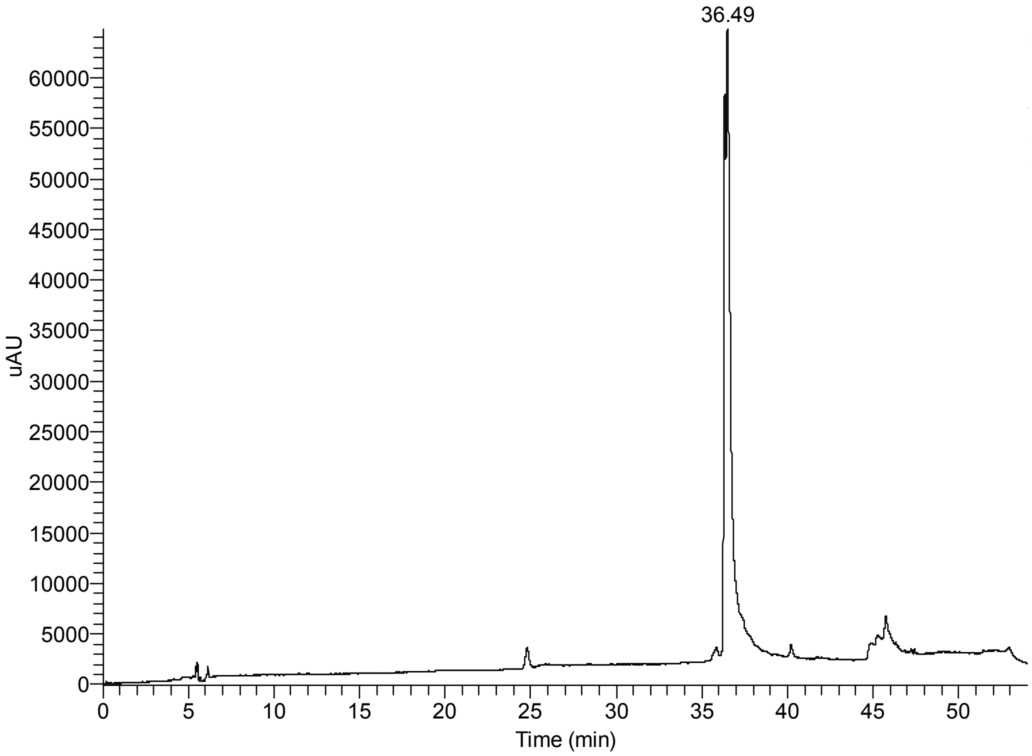
36.49
uAU
Time (min)

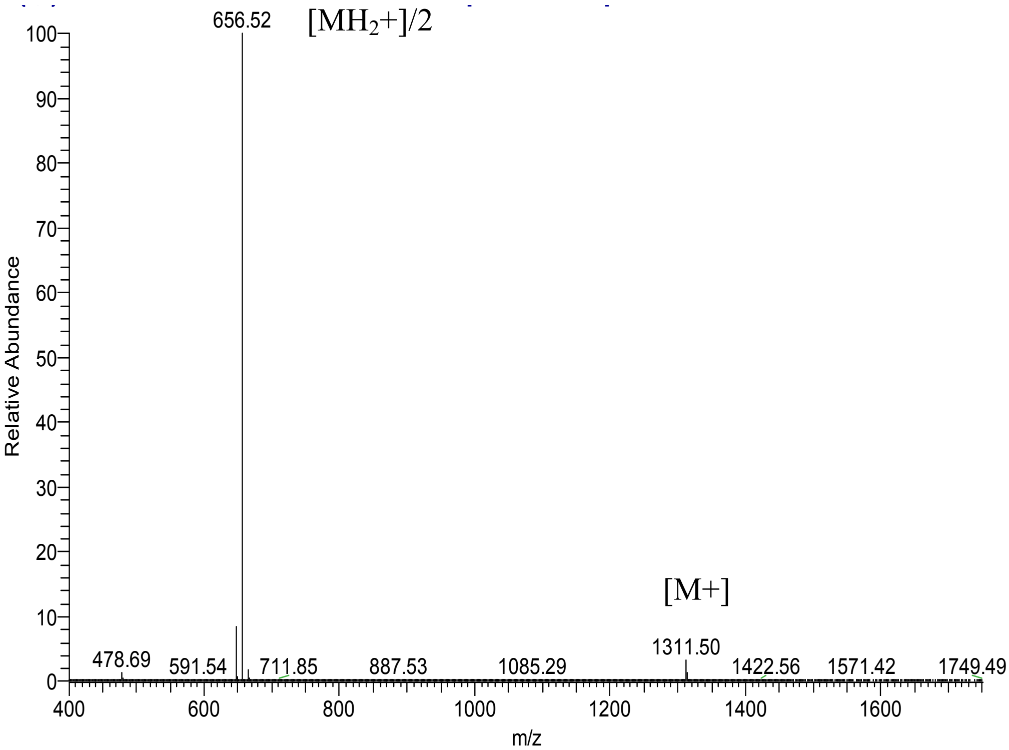
656.52
[MH2+]/2
[M+]
1311.50
478.69
591.54
711.85
887.53
1085.29
1422.56
1571.42
1749.49
Relative Abundance
m/z

**Compound 4.**

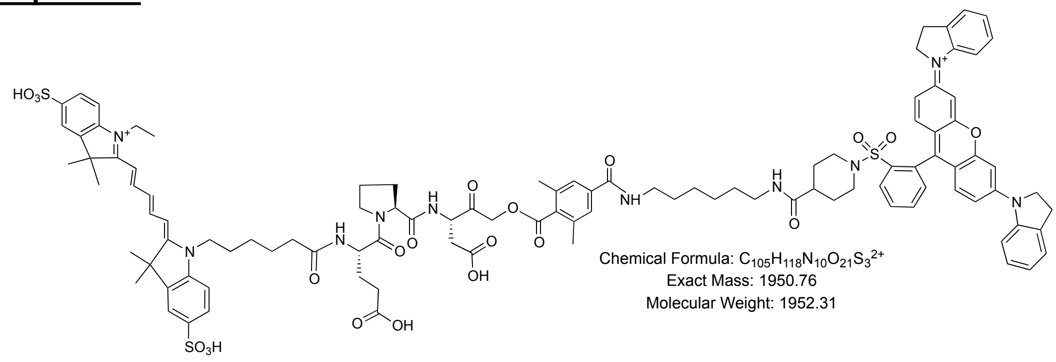

Chemical Formula: $C_{105}H_{118}N_{10}O_{21}S_3^{2+}$
Exact Mass: 1950.76
Molecular Weight: 1952.31


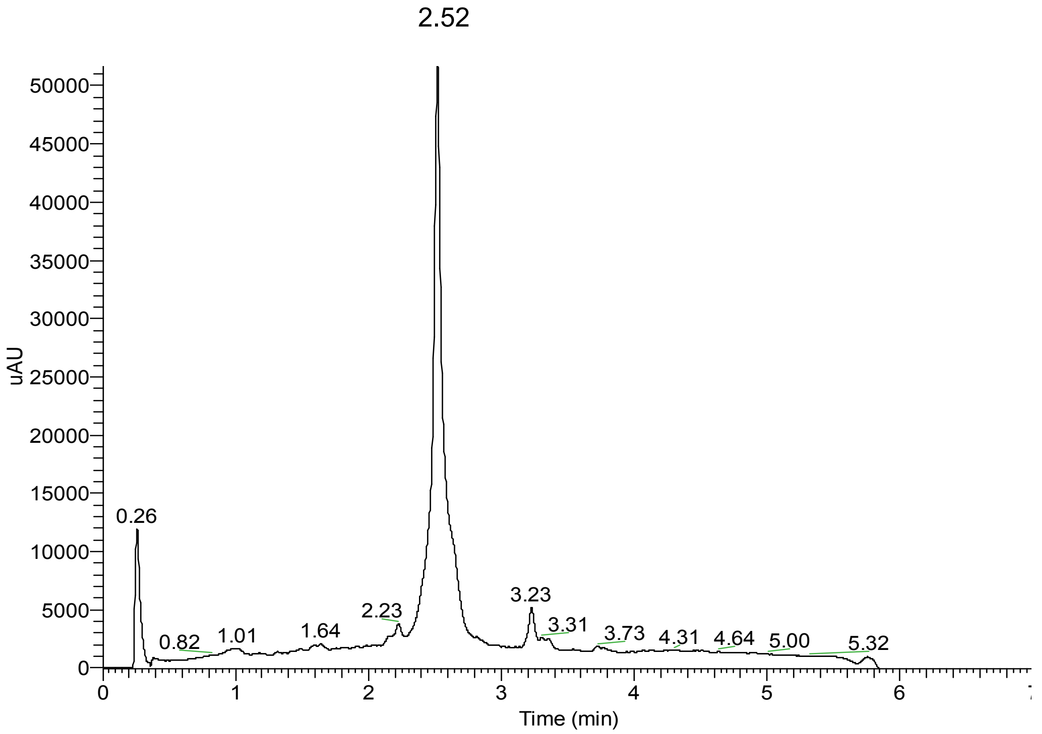

2.52
uAU
0.26
0.82
1.01
1.64
2.23
3.23
3.31
3.73
4.31
4.64
5.00
5.32
Time (min)


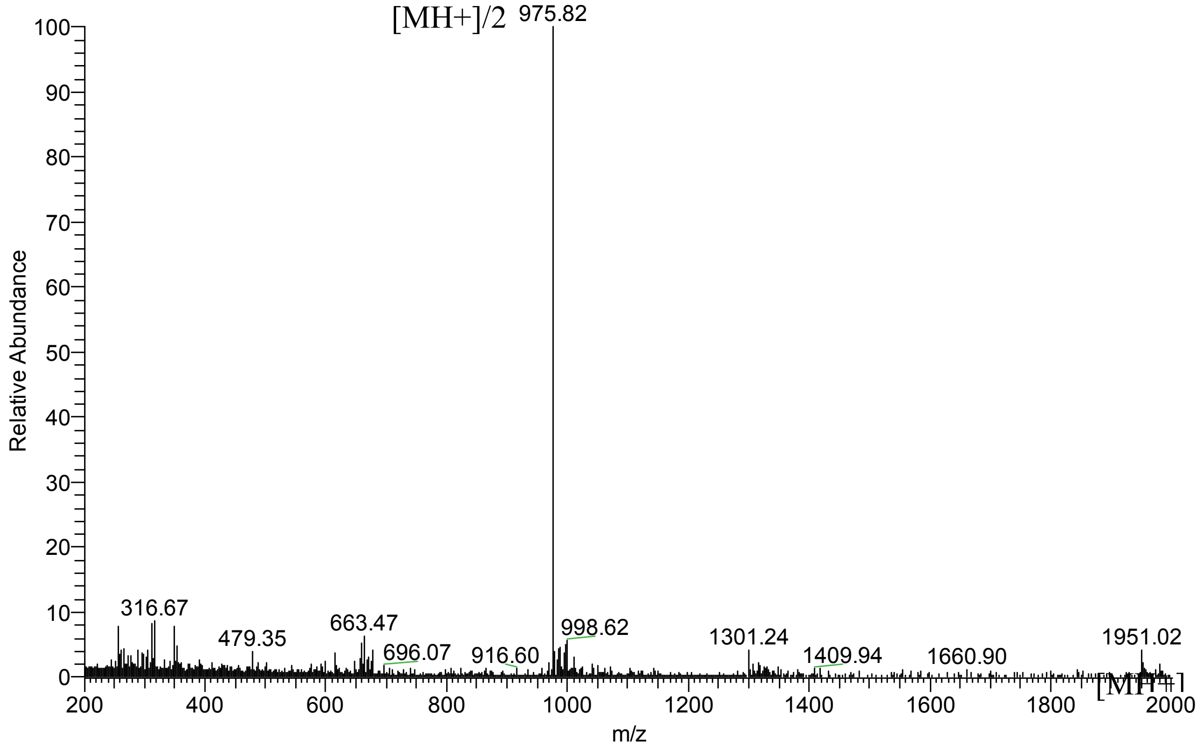

[MH+]/2
975.82
Relative Abundance
316.67
479.35
663.47
696.07
916.60
998.62
1301.24
1409.94
1660.90
1951.02
[MH+]
m/z

**Compound 5.**

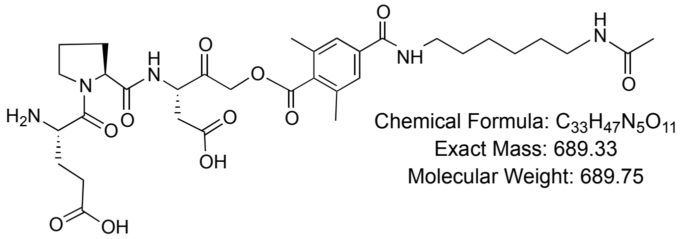
Chemical Formula: $C_{33}H_{47}N_5O_{11}$
Exact Mass: 689.33
Molecular Weight: 689.75

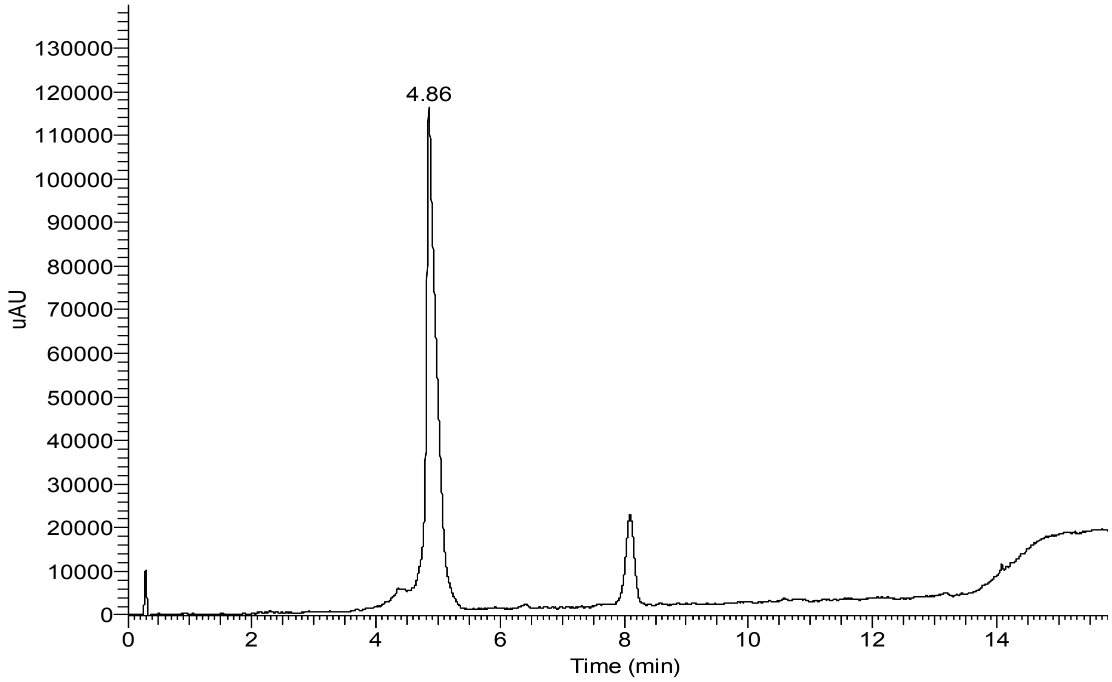
4.86
uAU
Time (min)

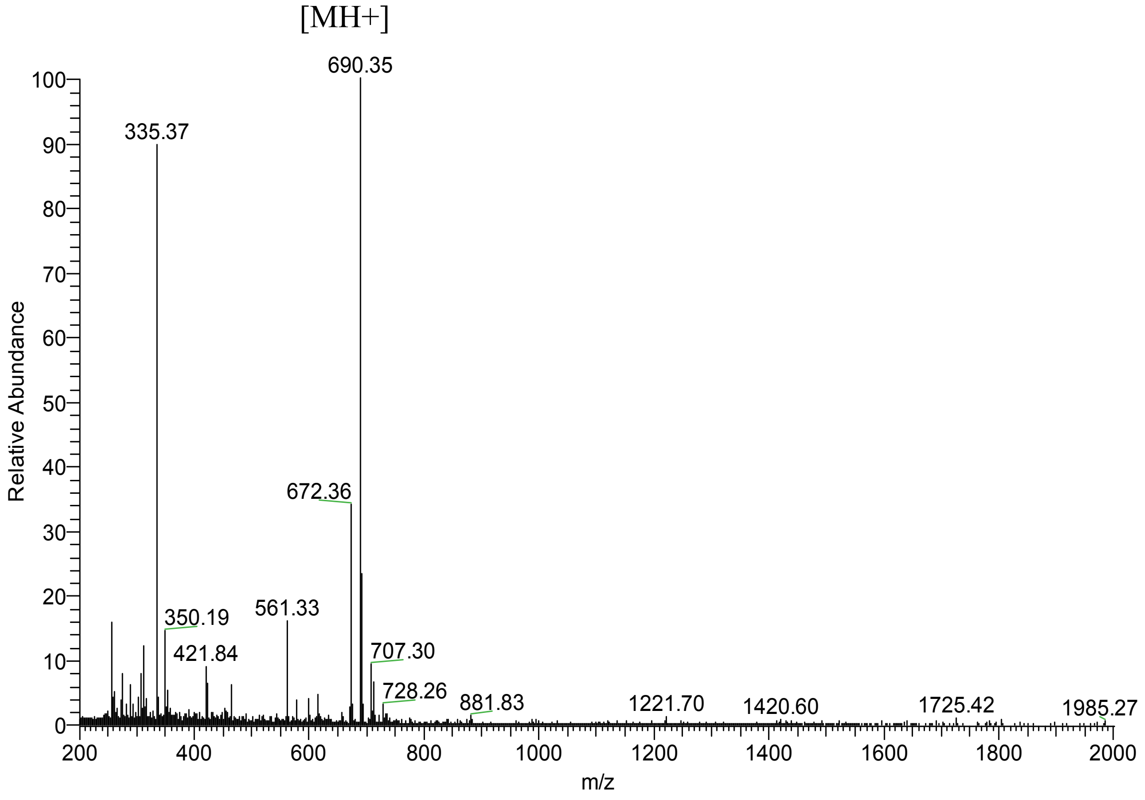
[MH+]
690.35
335.37
672.36
350.19
421.84
561.33
707.30
728.26
881.83
1221.70
1420.60
1725.42
1985.27
Relative Abundance
m/z

**Compound 6.**

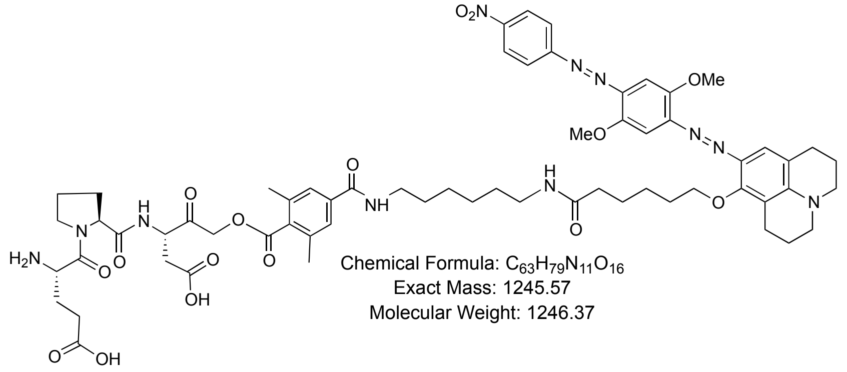

Chemical Formula: $C_{63}H_{79}N_{11}O_{16}$
Exact Mass: 1245.57
Molecular Weight: 1246.37


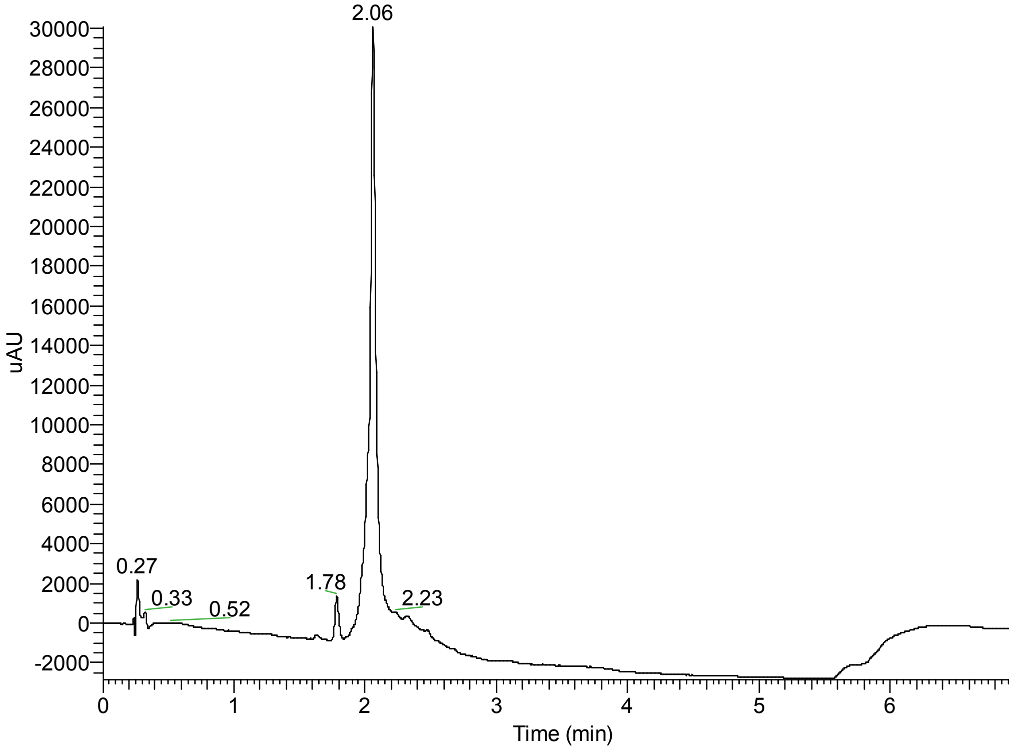

2.06
0.27
0.33
0.52
1.78
2.23
uAU
Time (min)


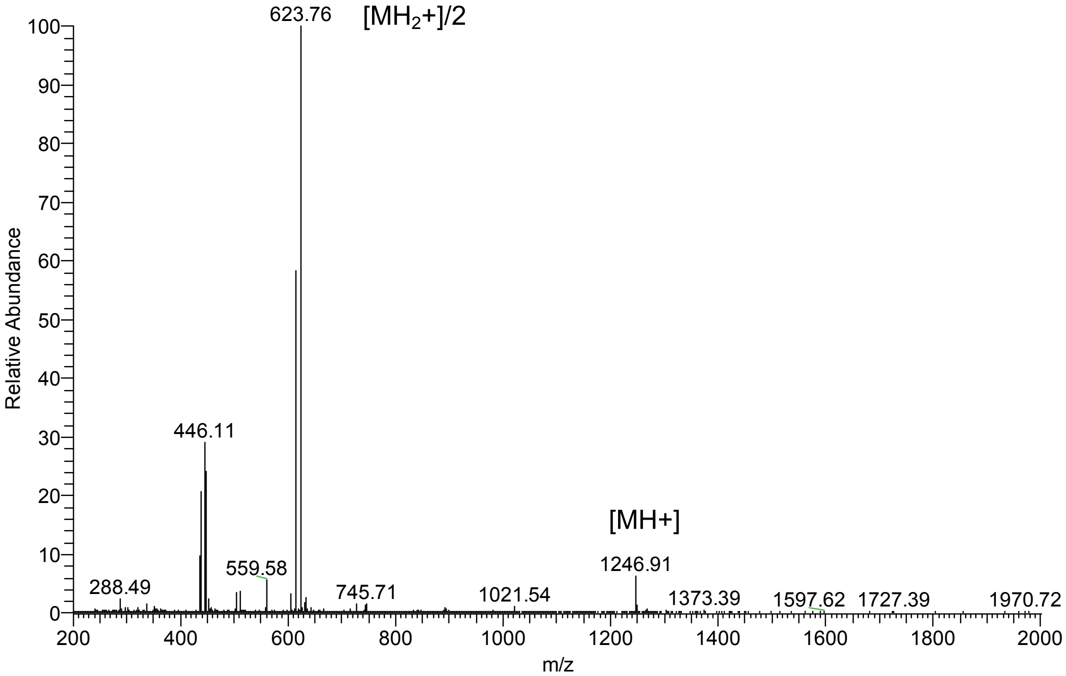

623.76
[MH2+]/2
446.11
[MH+]
1246.91
559.58
288.49
745.71
1021.54
1373.39
1597.62
1727.39
1970.72
Relative Abundance
m/z

**Compound 7.**

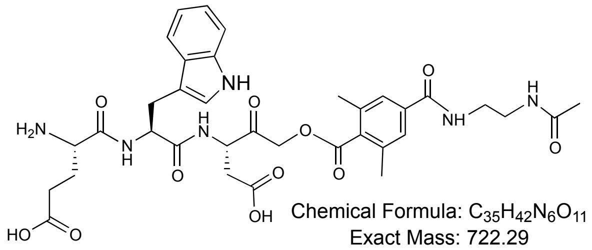

Chemical Formula: $C_{35}H_{42}N_6O_{11}$
Exact Mass: 722.29
Molecular Weight: 722.74


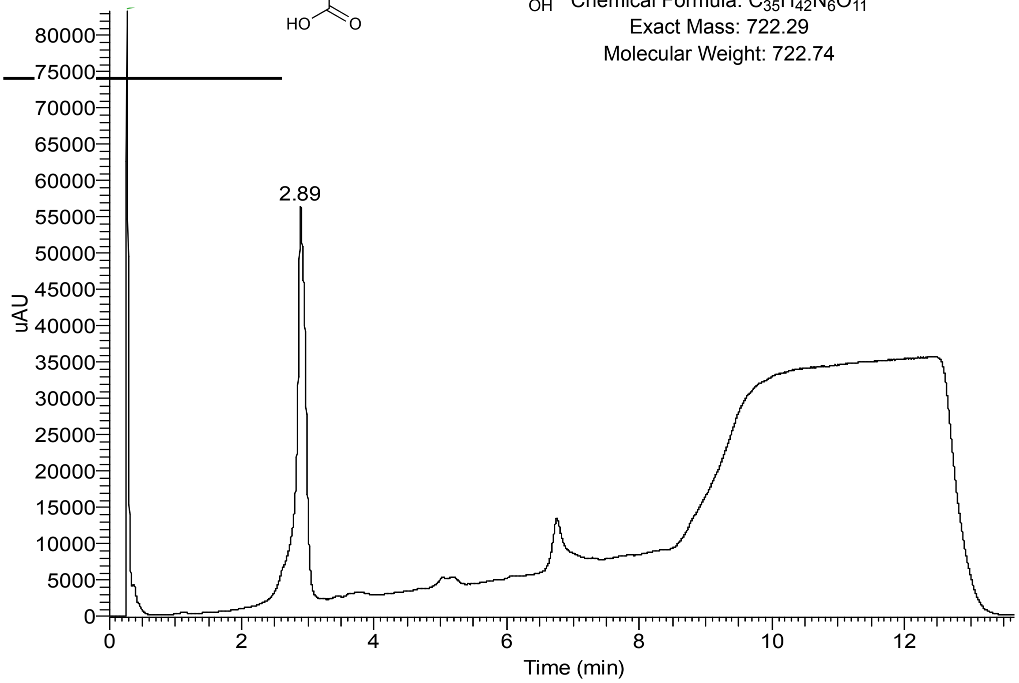

2.89
uAU
Time (min)


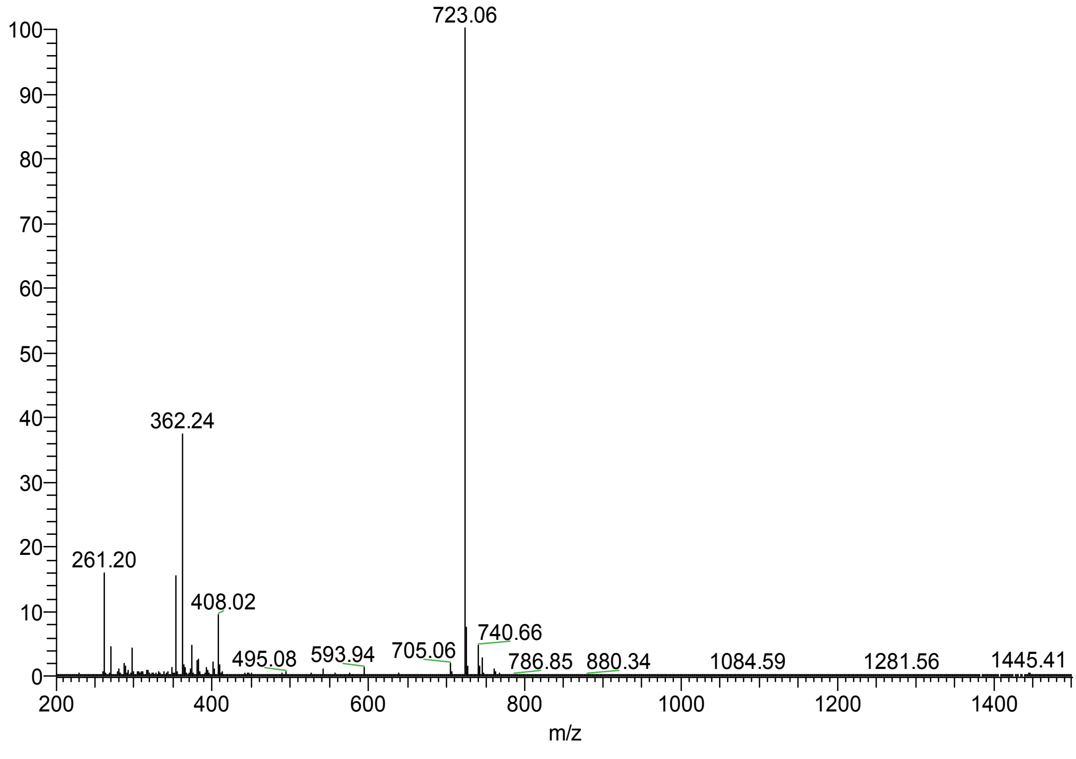

[MH+]
723.06
362.24
261.20
408.02
495.08
593.94
705.06
740.66
786.85
880.34
1084.59
1281.56
1445.41
m/z

**Compound 8.**

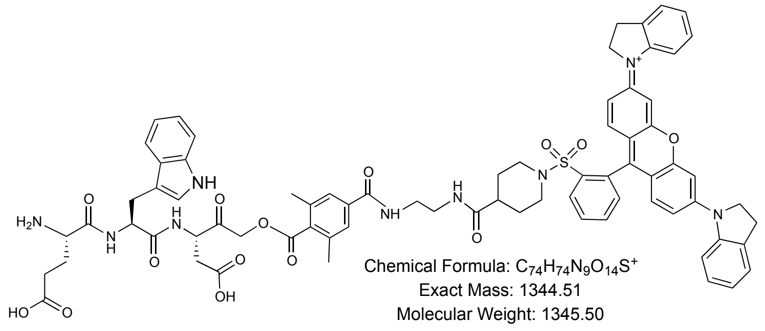
H2N
NH
HO
OH
Chemical Formula: C74H74N9O14S+
Exact Mass: 1344.51
Molecular Weight: 1345.50

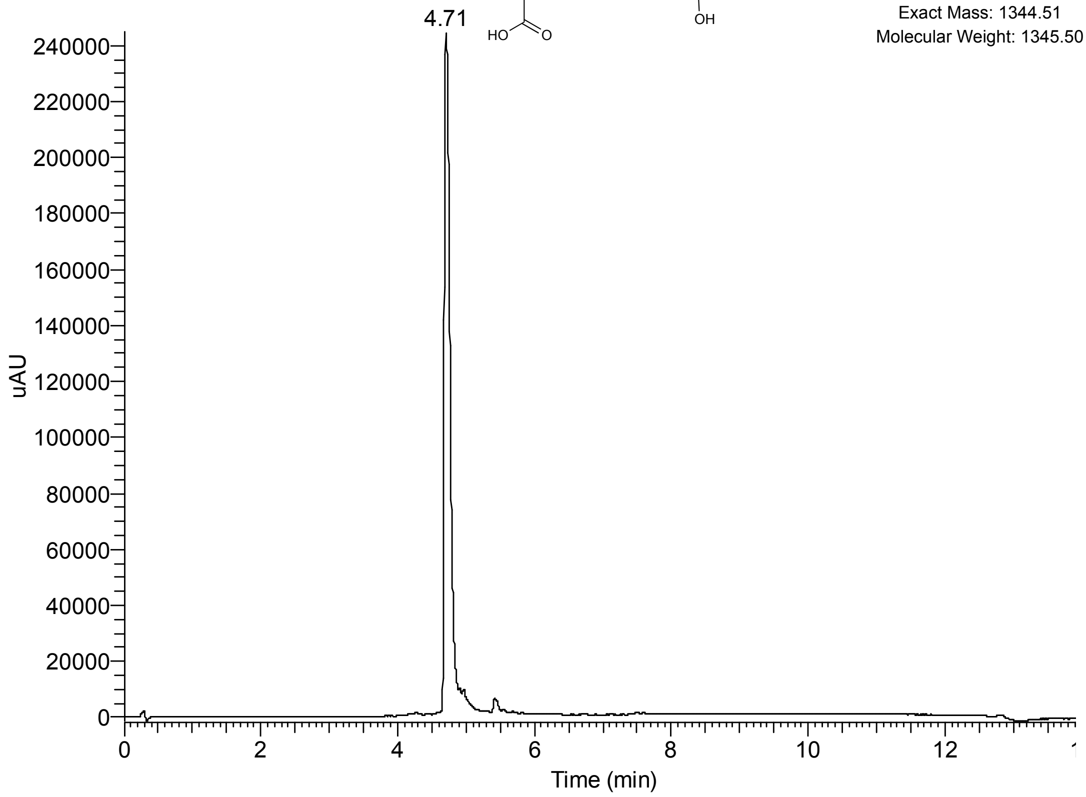
4.71
uAU
Time (min)

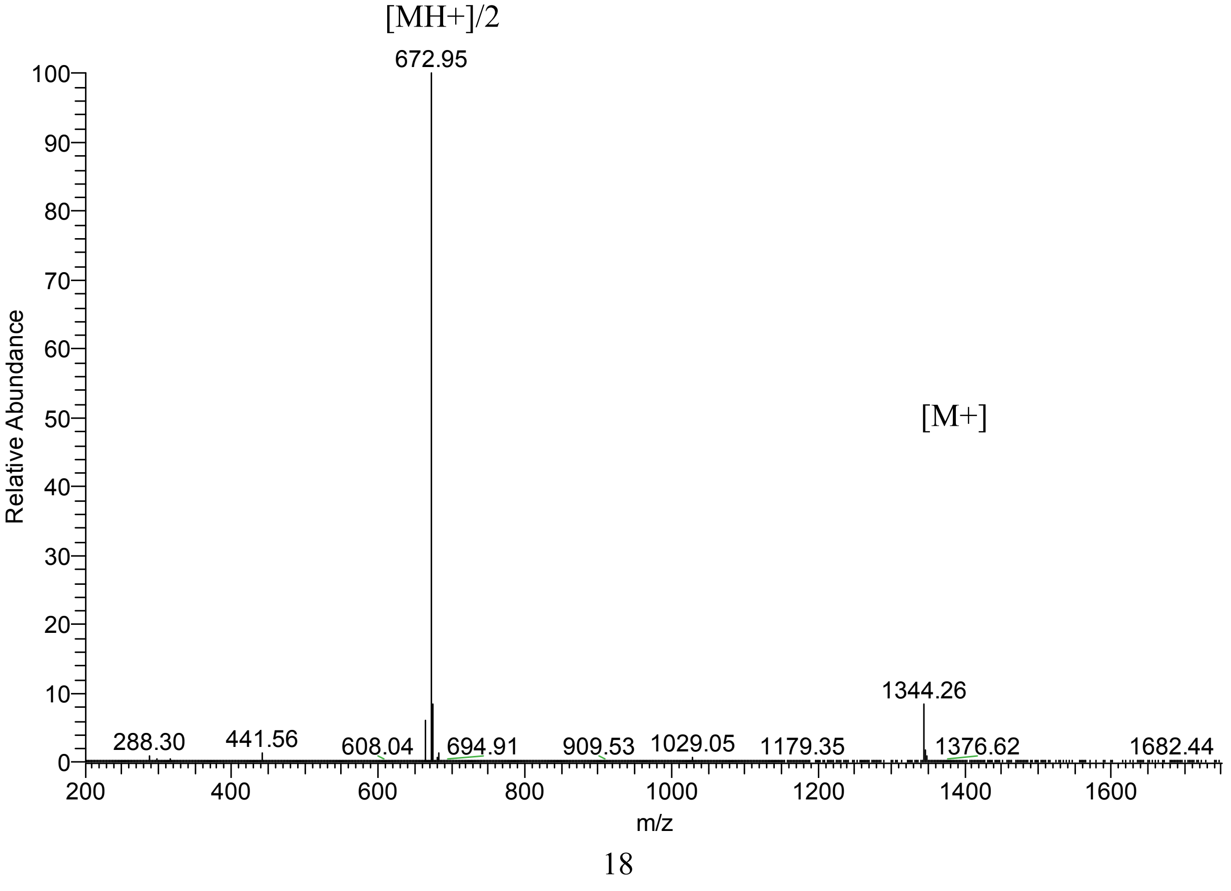
[MH+]/2
672.95
[M+]
1344.26
Relative Abundance
m/z

**Compound 9.**

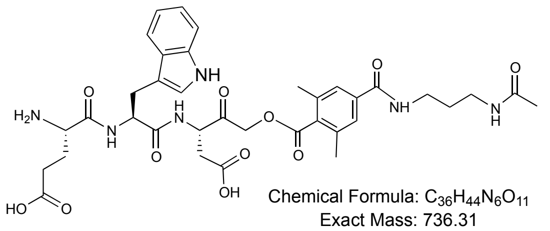


Chemical Formula: $C_{36}H_{44}N_6O_{11}$
Exact Mass: 736.31
Molecular Weight: 736.77

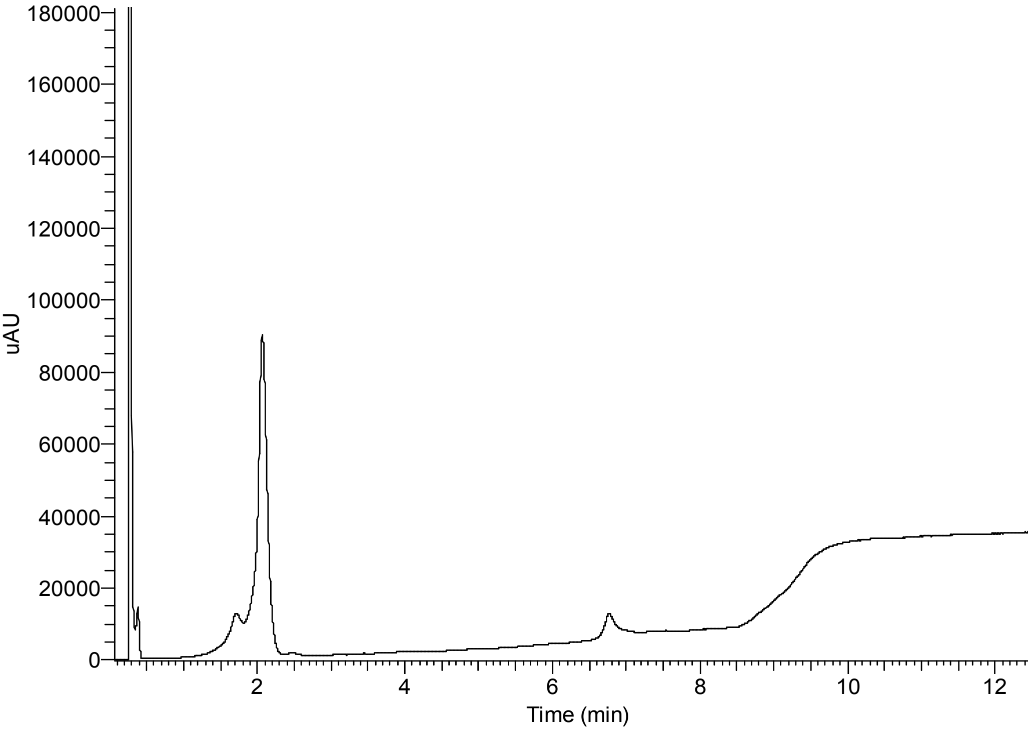


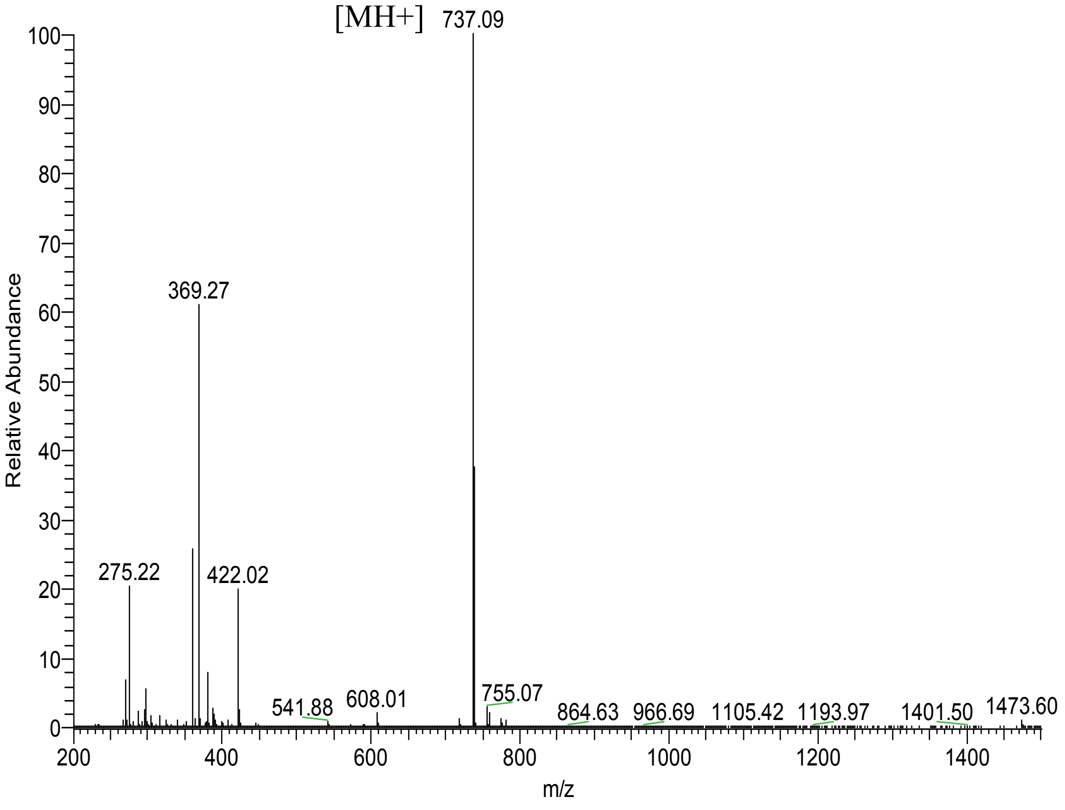

**Compound 10.**

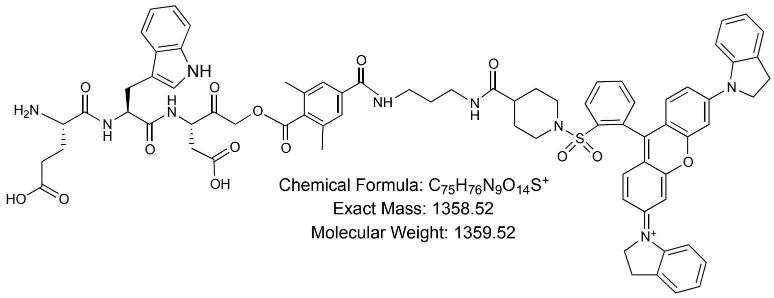
H2N
NH
HO
OH
Chemical Formula: C75H76N9O14S+
Exact Mass: 1358.52
Molecular Weight: 1359.52

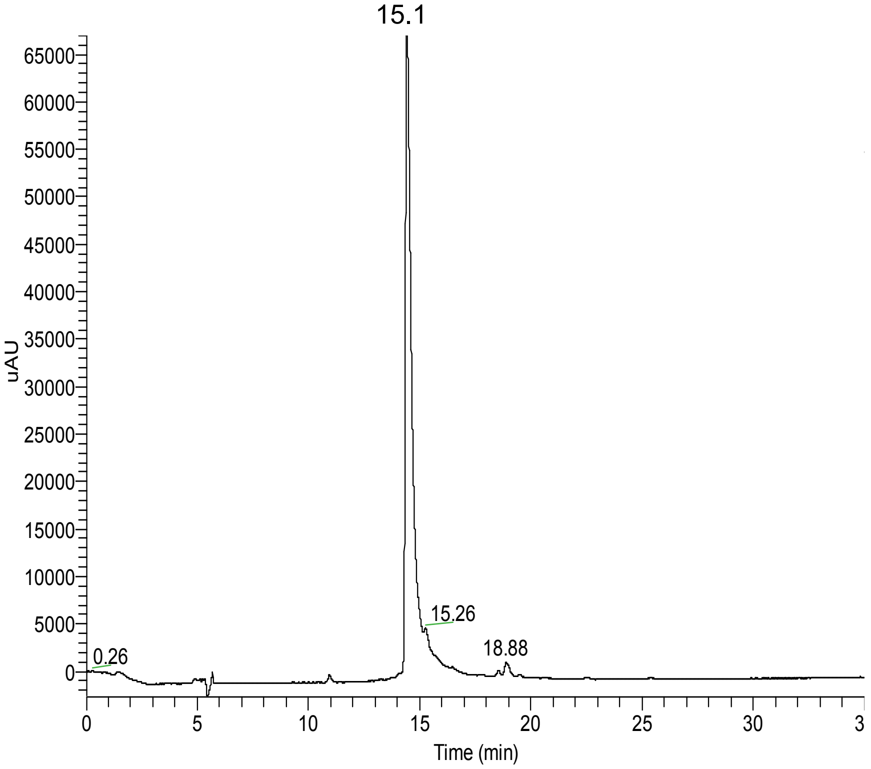
15.1
uAU
15.26
18.88
0.26
Time (min)

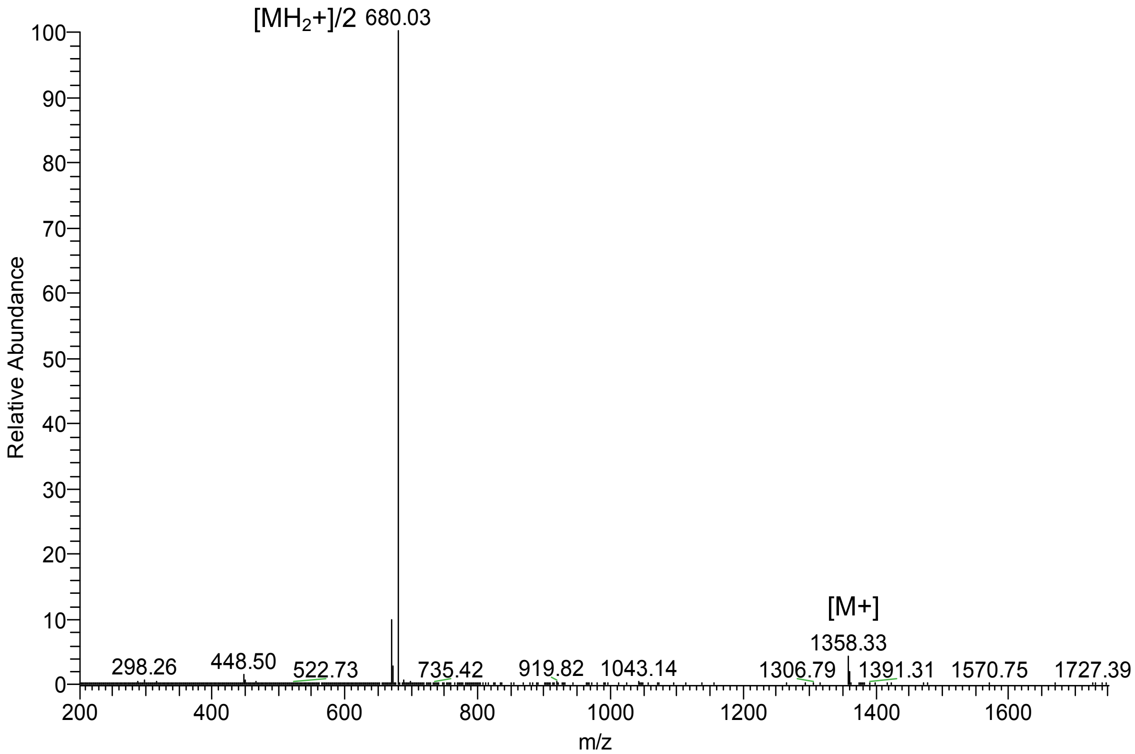
[MH2+]/2 680.03
Relative Abundance
[M+]
1358.33
298.26
448.50
522.73
735.42
919.82
1043.14
1306.79
1391.31
1570.75
1727.39
m/z

## Compound 11.

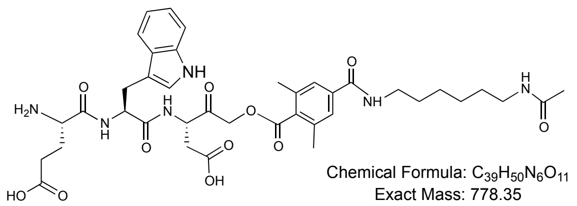
Chemical Formula: $C_{39}H_{50}N_6O_{11}$
Exact Mass: 778.35
Molecular Weight: 778.85

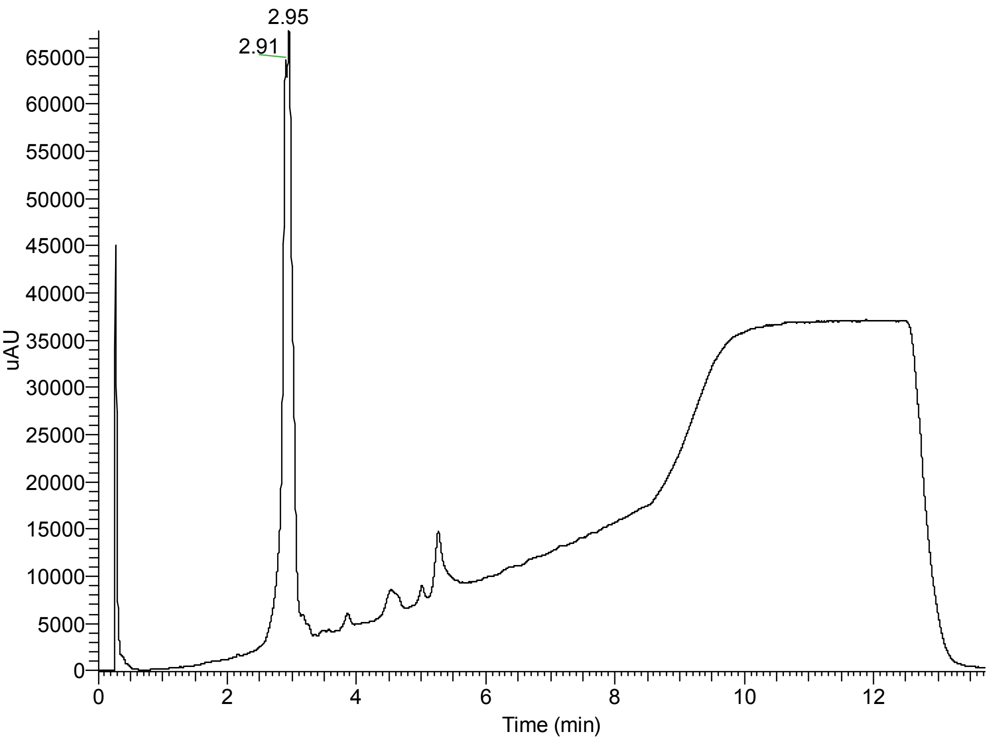
2.95
2.91
uAU
Time (min)

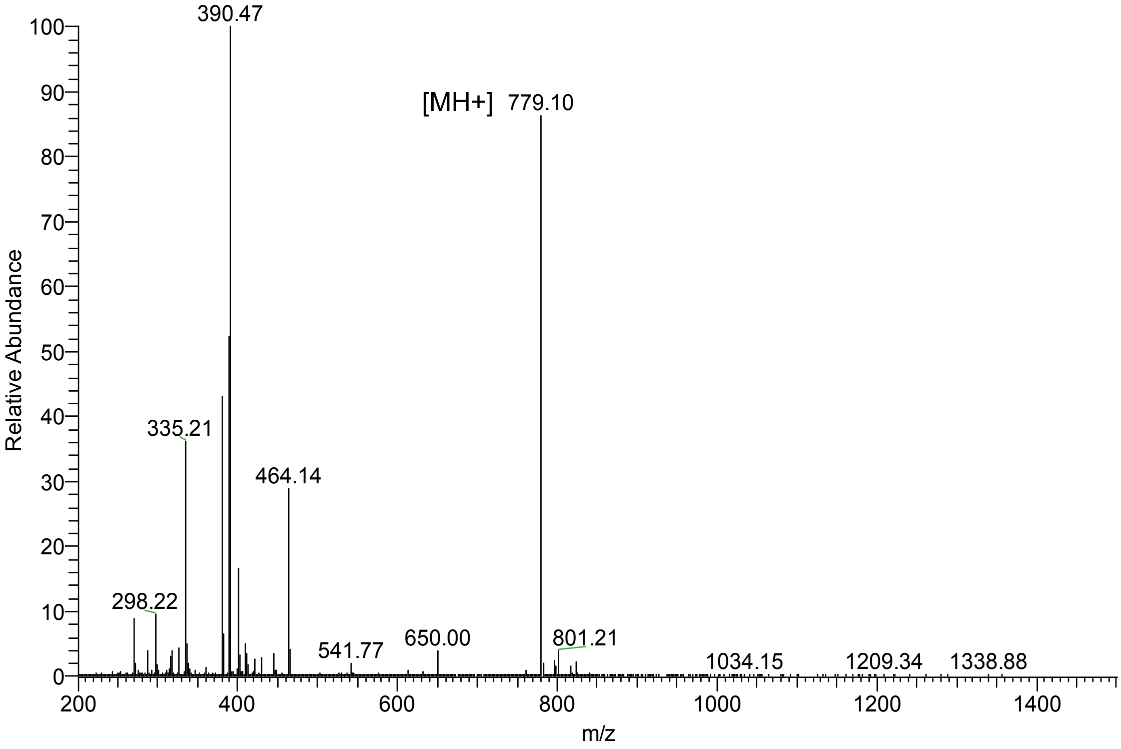
390.47
[MH+] 779.10
335.21
464.14
298.22
541.77
650.00
801.21
1034.15
1209.34
1338.88
Relative Abundance
m/z

**Compound 12.**

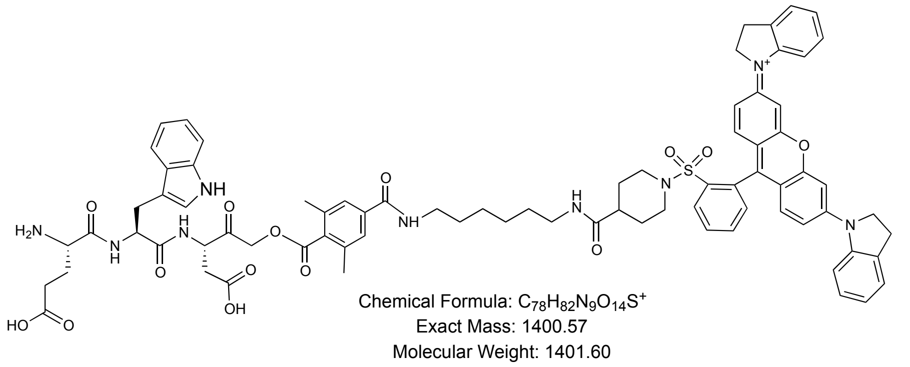
H2N
NH
HO
OH
Chemical Formula: C78H82N9O14S+
Exact Mass: 1400.57
Molecular Weight: 1401.60

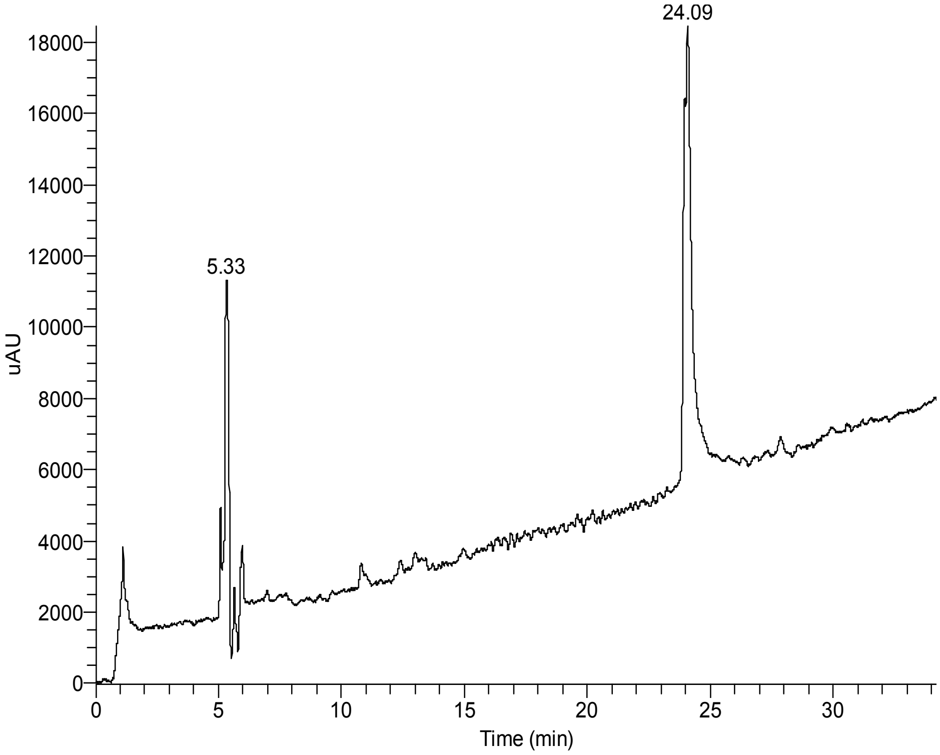
24.09
5.33
uAU
Time (min)

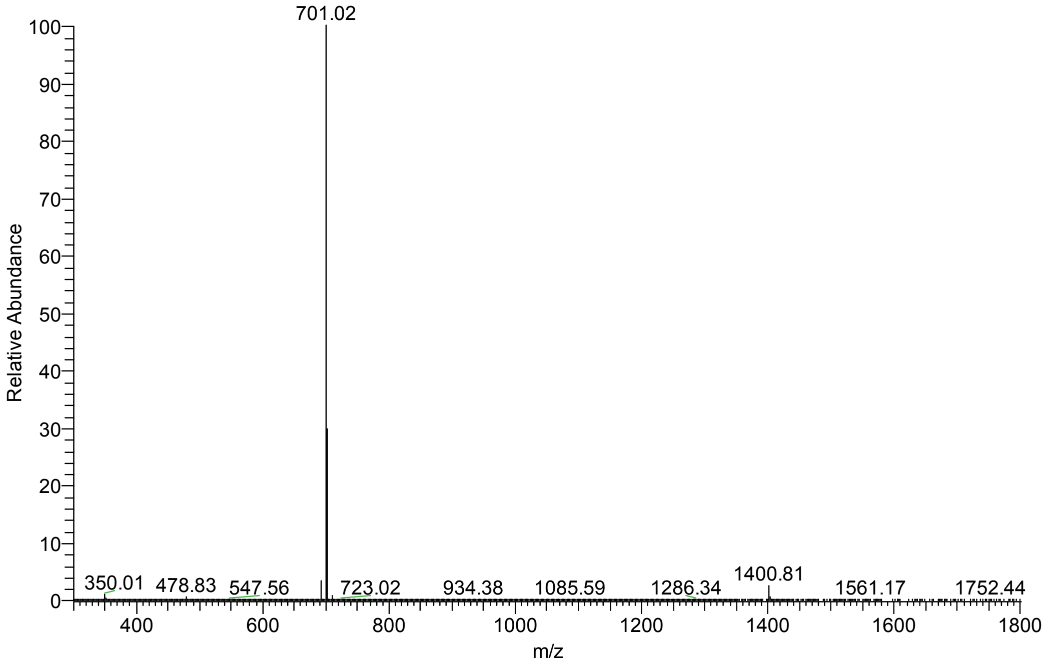
701.02
350.01
478.83
547.56
723.02
934.38
1085.59
1286.34
1400.81
1561.17
1752.44
Relative Abundance
m/z

**Compound 13.**

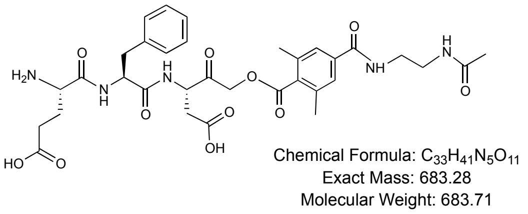

H2N
HO
OH
NH
Chemical Formula: C33H41N5O11
Exact Mass: 683.28
Molecular Weight: 683.71


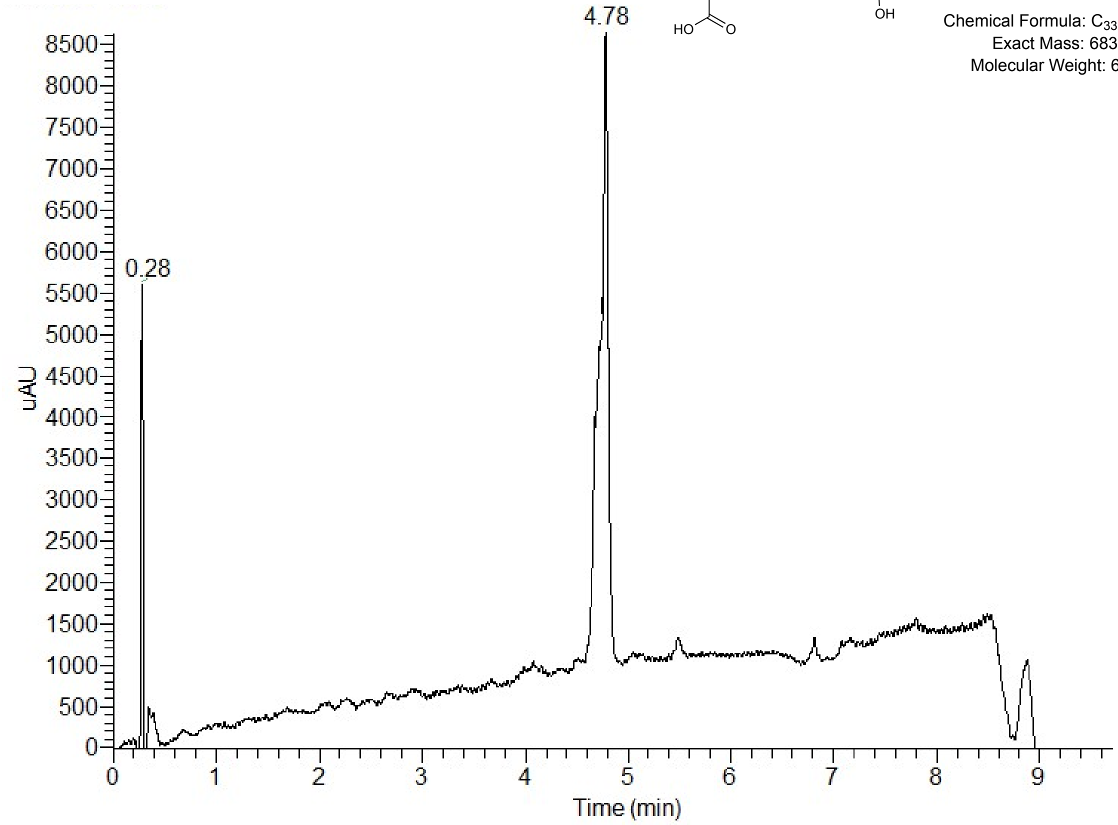

4.78
0.28
uAU
Time (min)


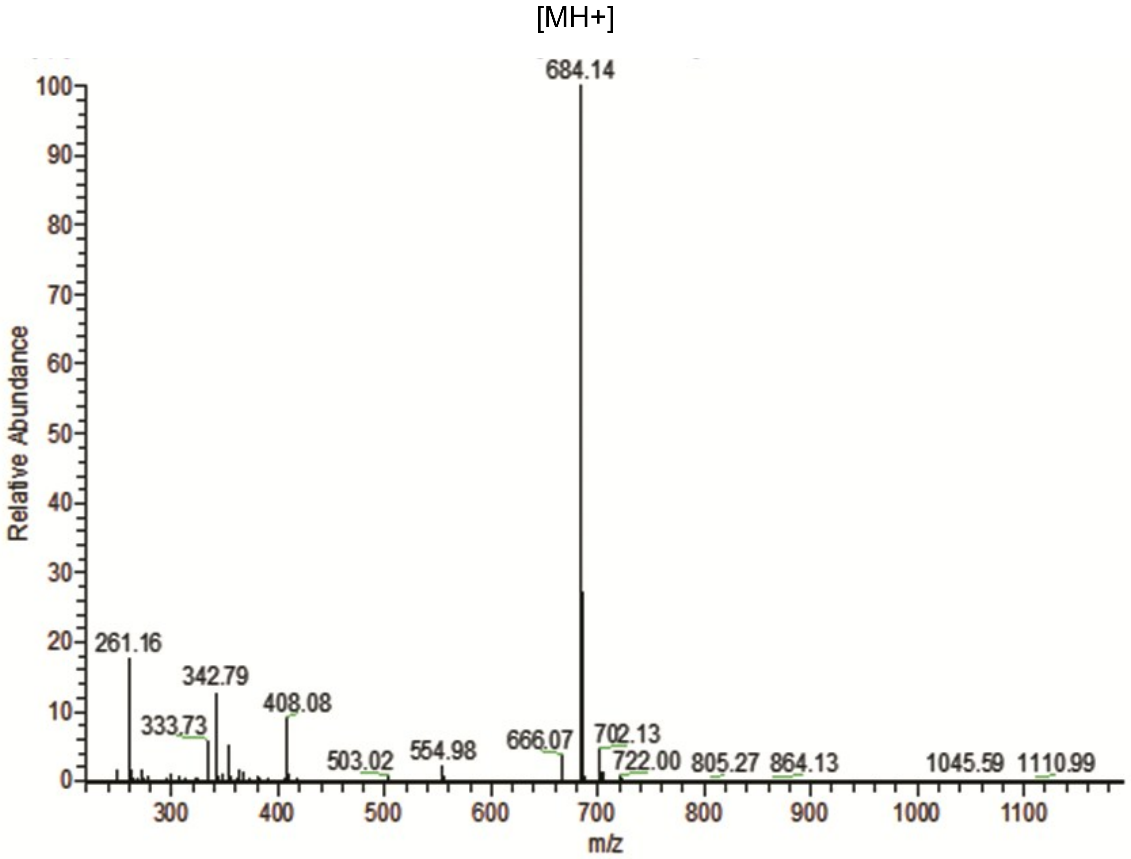

[MH+]
684.14
261.16
342.79
333.73
408.08
503.02
554.98
666.07
702.13
722.00
805.27
864.13
1045.59
1110.99
Relative Abundance
m/z

**Compound 14.**

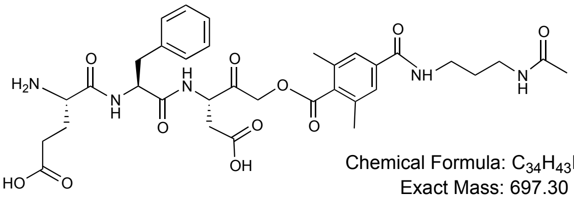


Chemical Formula: $C_{34}H_{43}N_5O_{11}$
Exact Mass: 697.30
Molecular Weight: 697.73

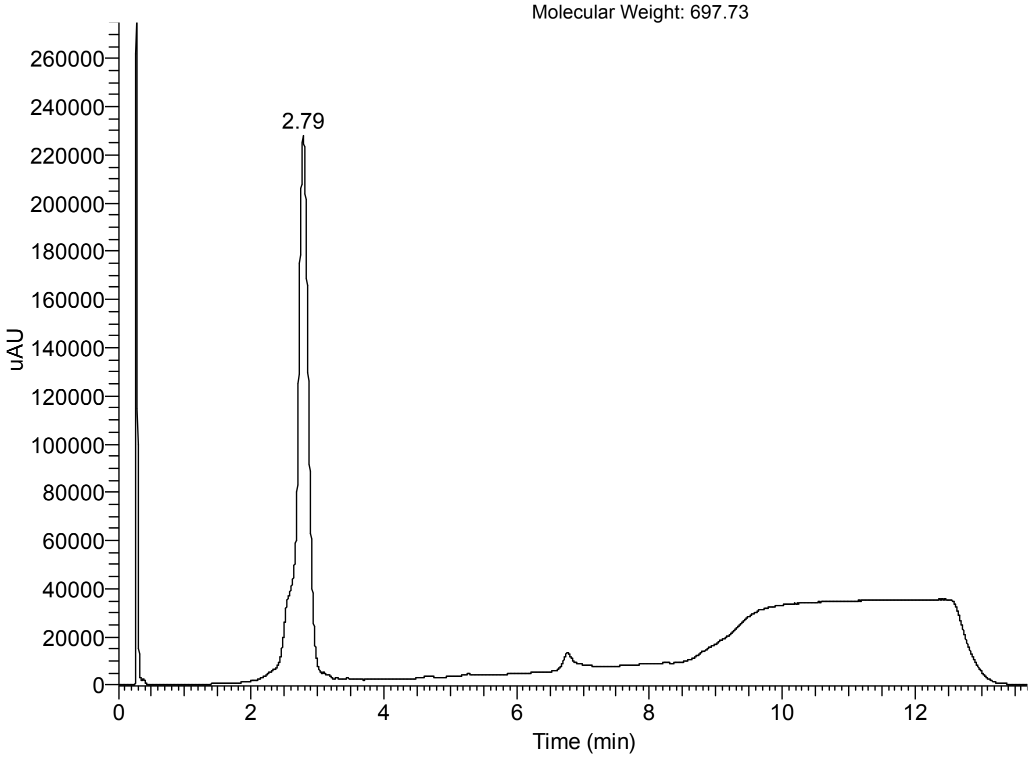


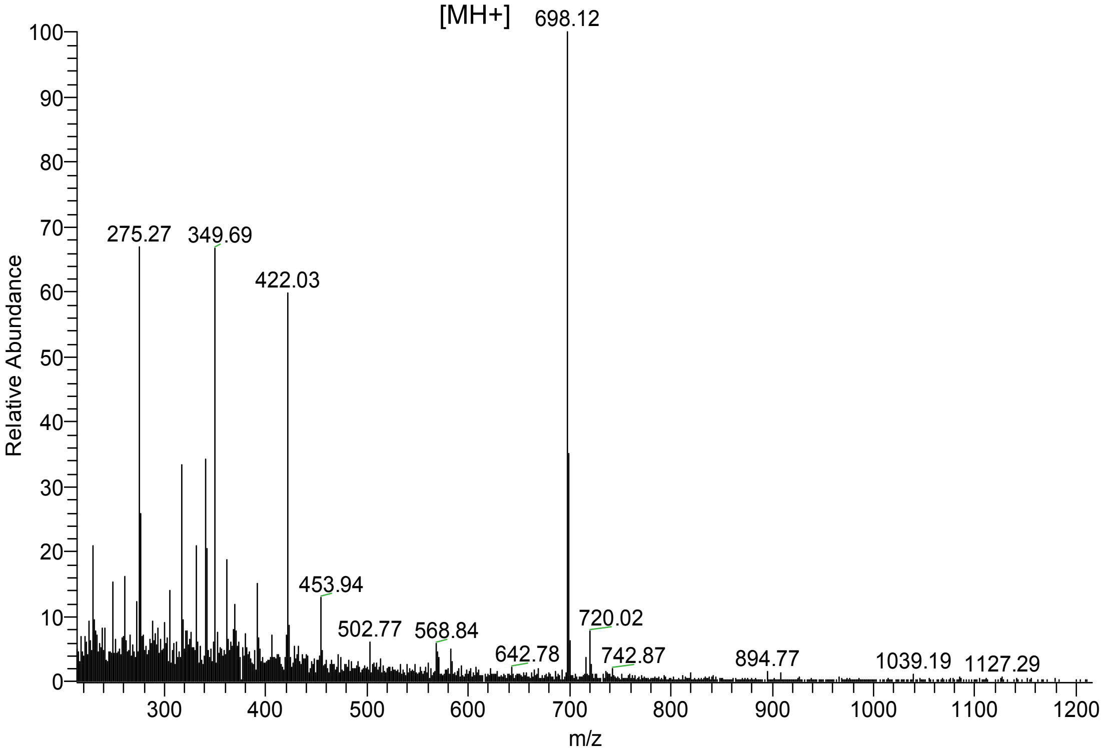

**Compound 15.**

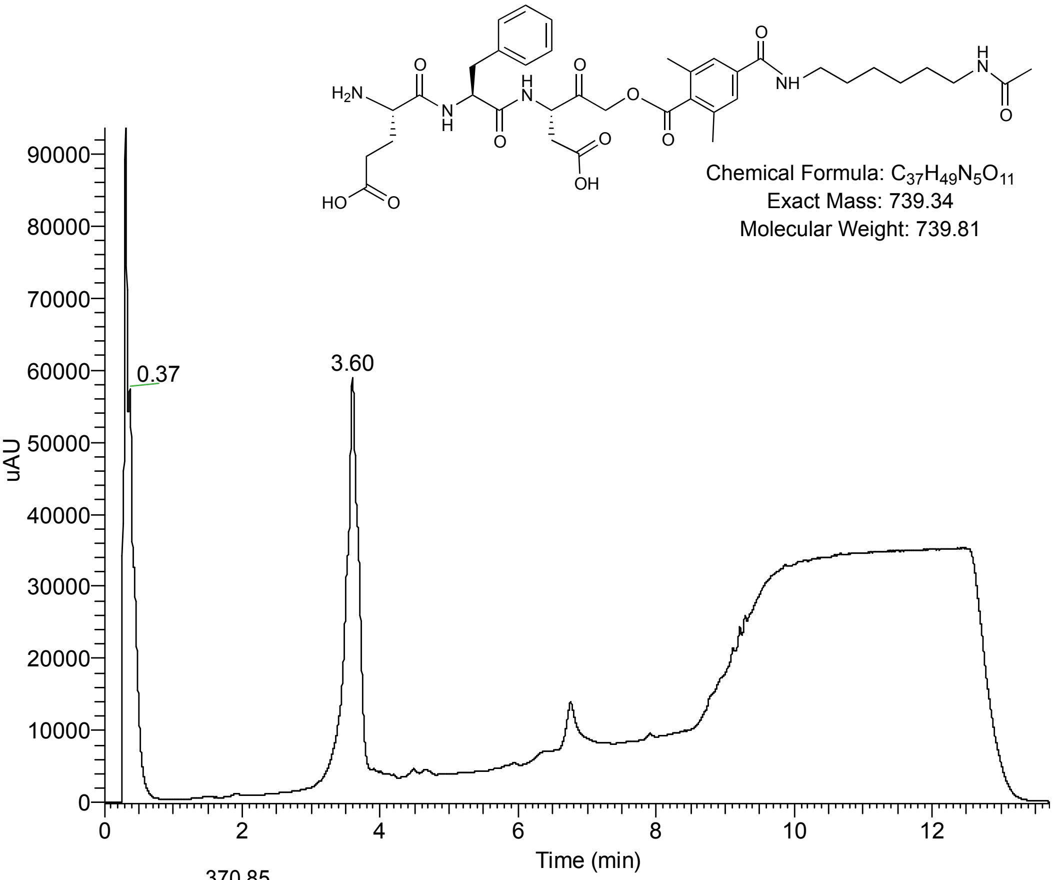
H2N
HO
OH
NH
Chemical Formula: C37H49N5O11
Exact Mass: 739.34
Molecular Weight: 739.81
uAU
Time (min)
0.37
3.60

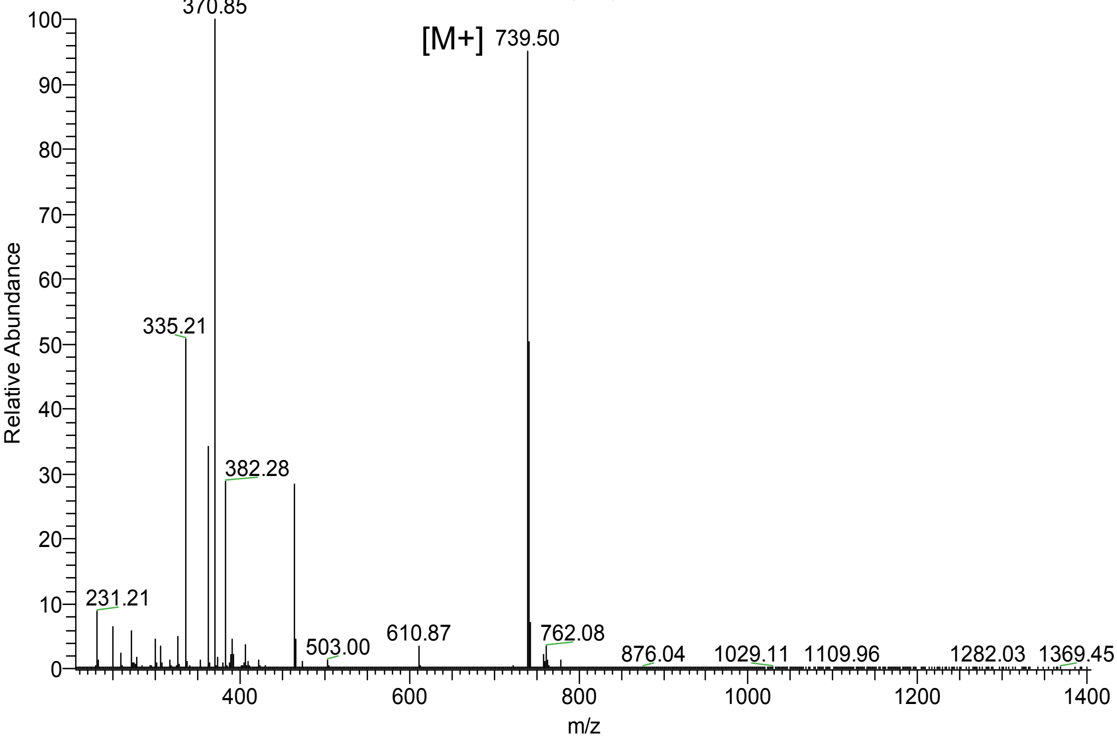
Relative Abundance
m/z
370.85
[M+] 739.50
335.21
382.28
231.21
503.00
610.87
762.08
876.04
1029.11
1109.96
1282.03
1369.45

**Compound 16.**

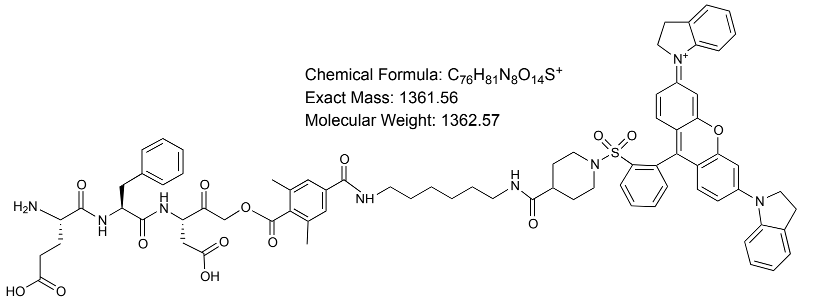
Chemical Formula: C76H81N8O14S+
Exact Mass: 1361.56
Molecular Weight: 1362.57

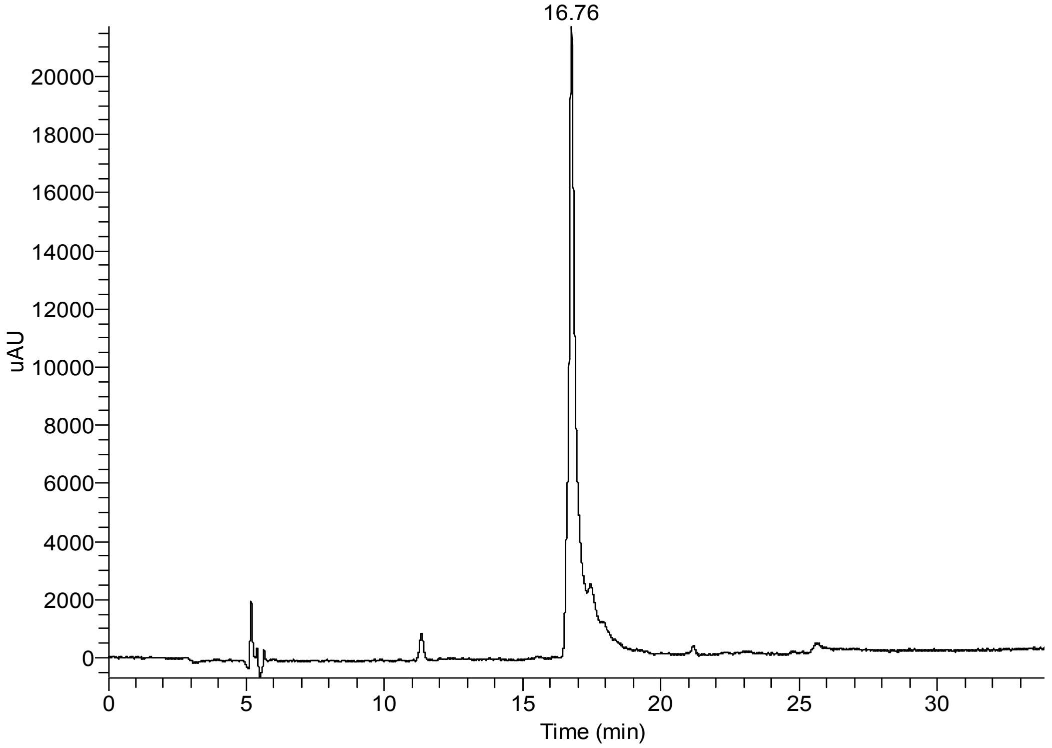
16.76
uAU
Time (min)

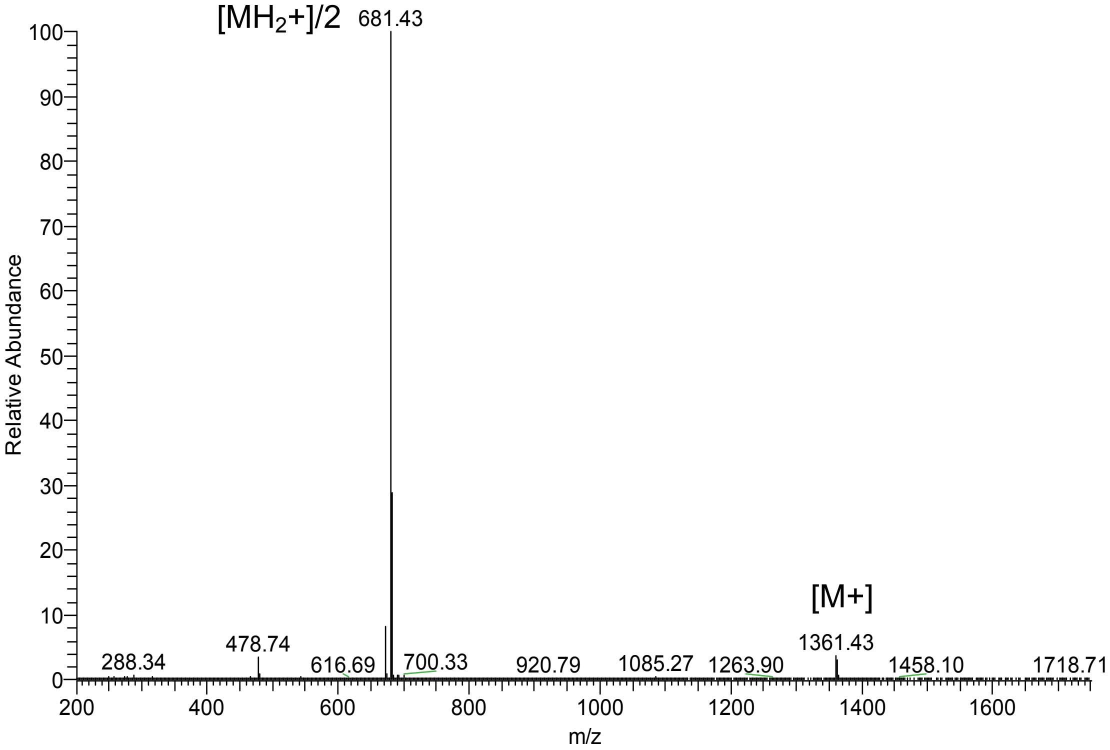
[MH2+]/2
681.43
[M+]
1361.43
288.34
478.74
616.69
700.33
920.79
1085.27
1263.90
1458.10
1718.71
Relative Abundance
m/z

**Compound 17.**

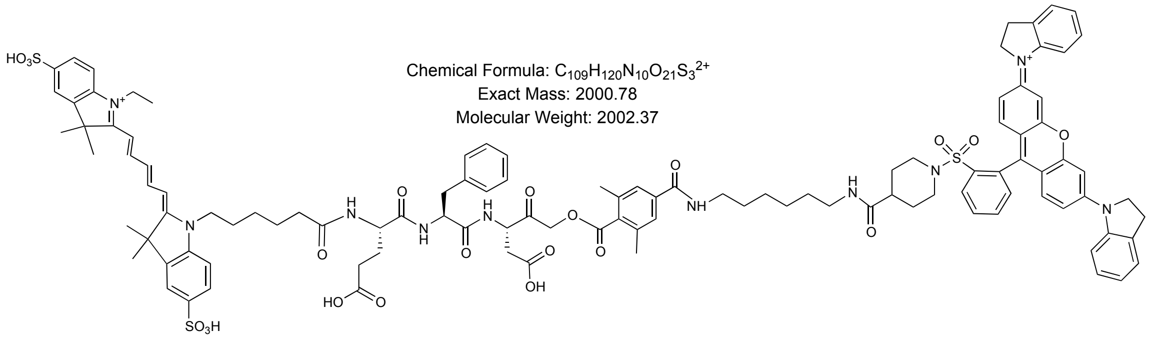
Chemical Formula: $C_{109}H_{120}N_{10}O_{21}S_3^{2+}$
Exact Mass: 2000.78
Molecular Weight: 2002.37

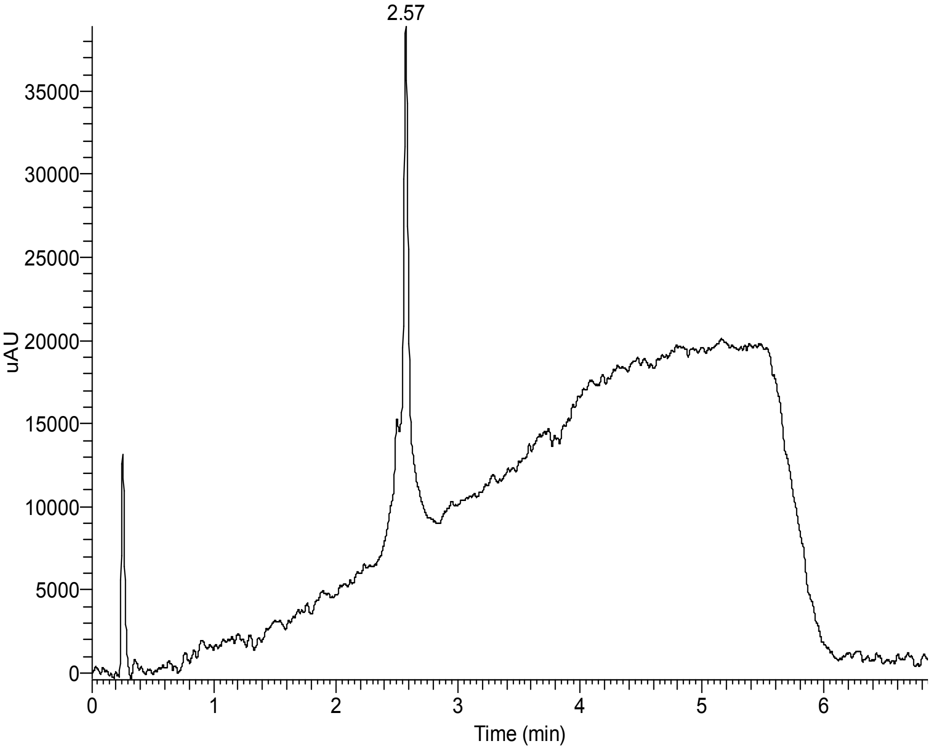
2.57
uAU
Time (min)

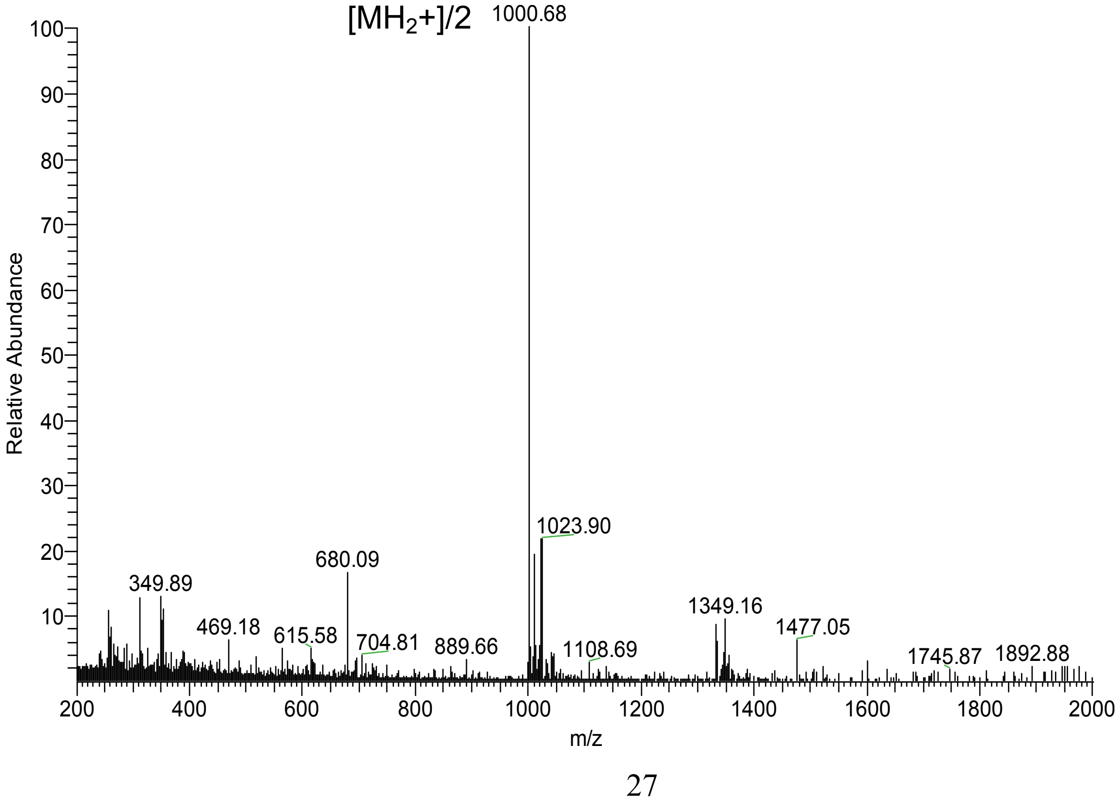
[MH2+]/2
1000.68
1023.90
680.09
349.89
469.18
615.58
704.81
889.66
1108.69
1349.16
1477.05
1745.87
1892.88
Relative Abundance
m/z

**Compound 18.**

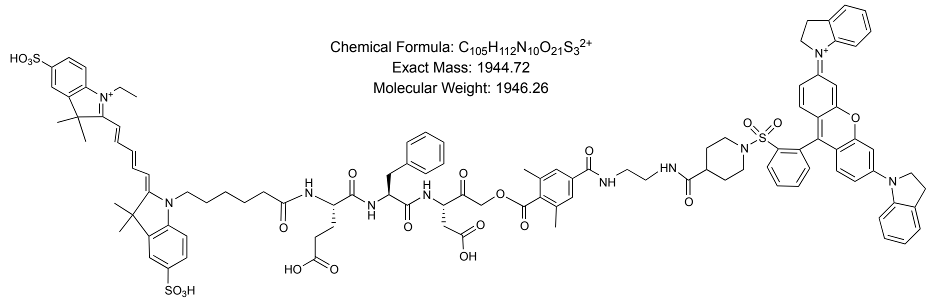
Chemical Formula: $C_{105}H_{112}N_{10}O_{21}S_3^{2+}$
Exact Mass: 1944.72
Molecular Weight: 1946.26

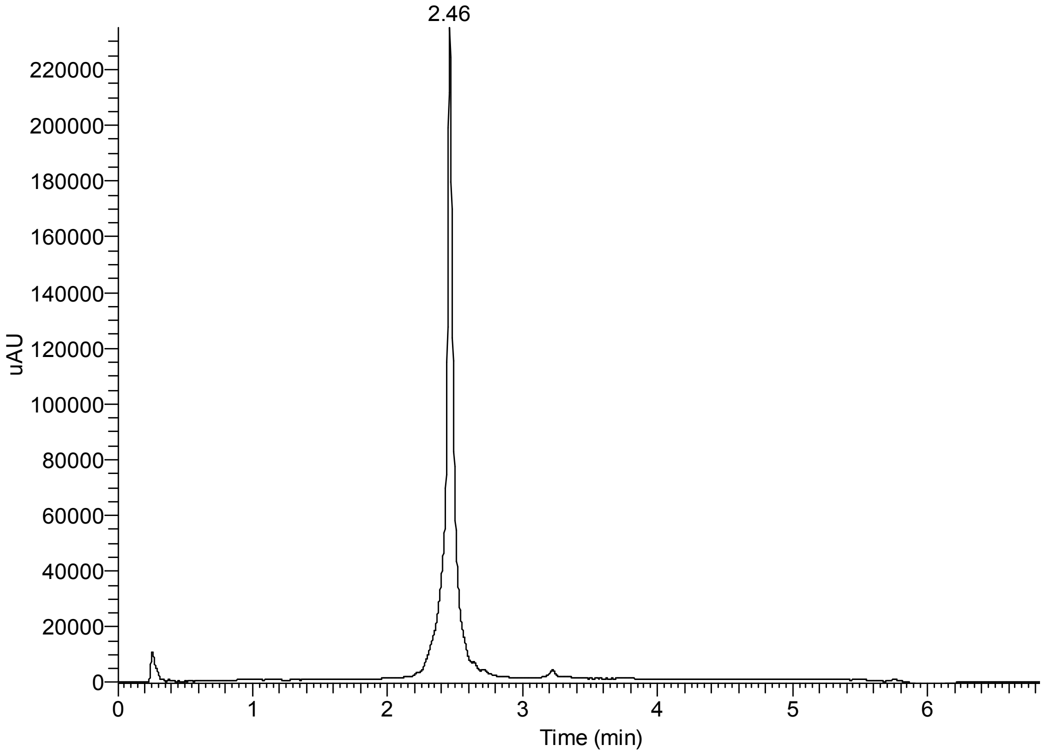
2.46
uAU
Time (min)

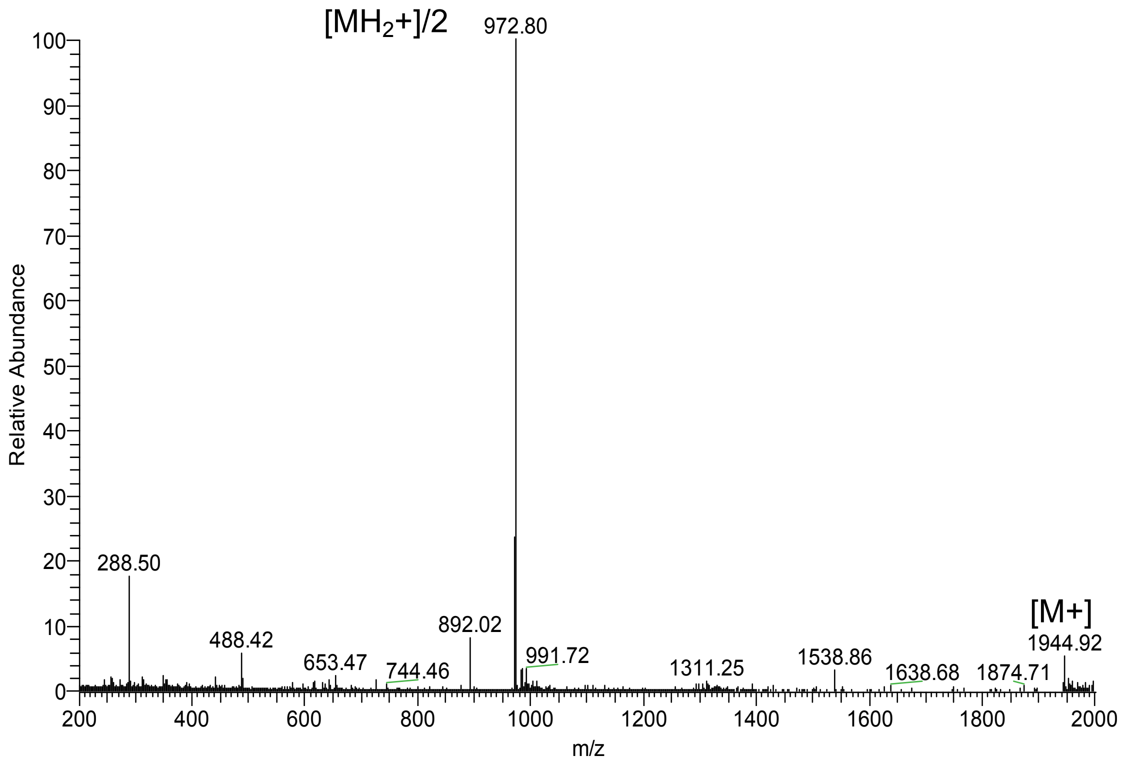
[MH2+]/2
972.80
288.50
488.42
653.47
744.46
892.02
991.72
1311.25
1538.86
1638.68
1874.71
[M+]
1944.92
Relative Abundance
m/z

## Compound 19.

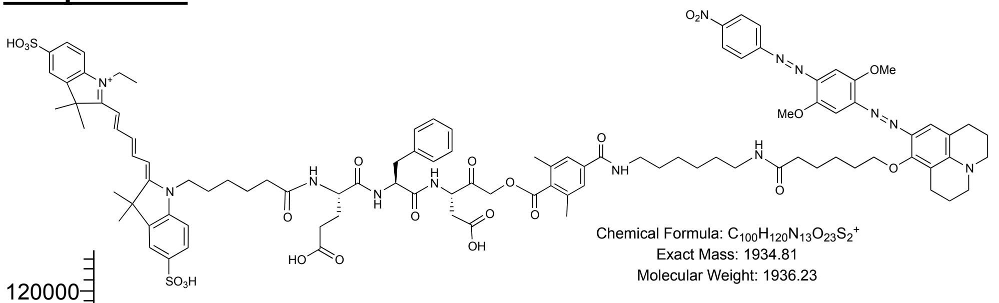
Chemical Formula: $C_{100}H_{120}N_{13}O_{23}S_2^+$
Exact Mass: 1934.81
Molecular Weight: 1936.23

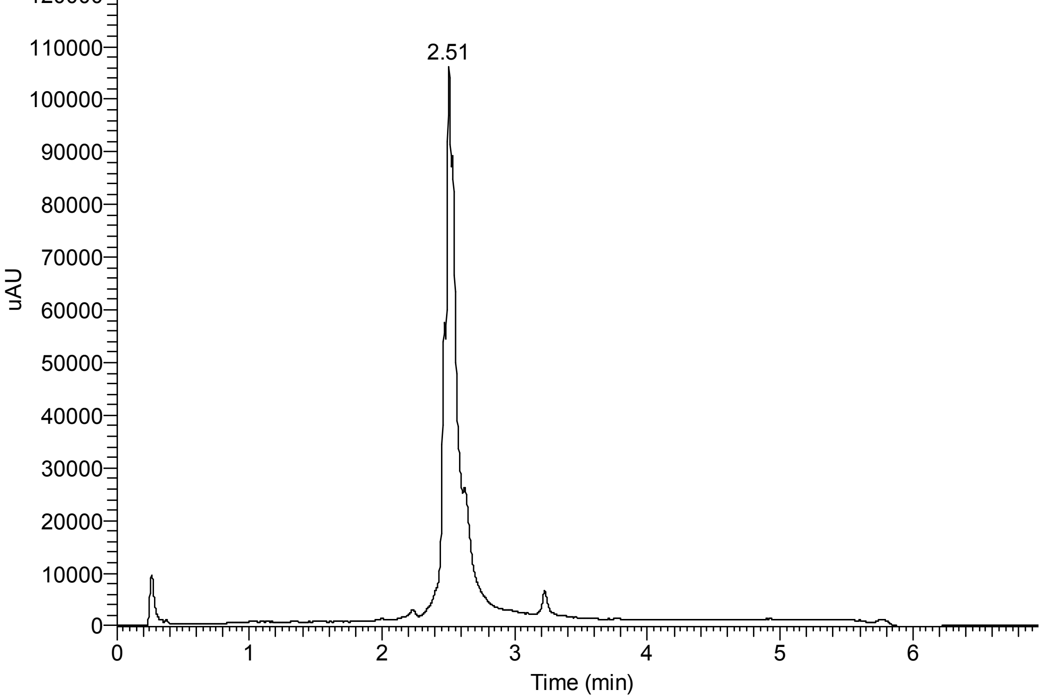
2.51
uAU
Time (min)

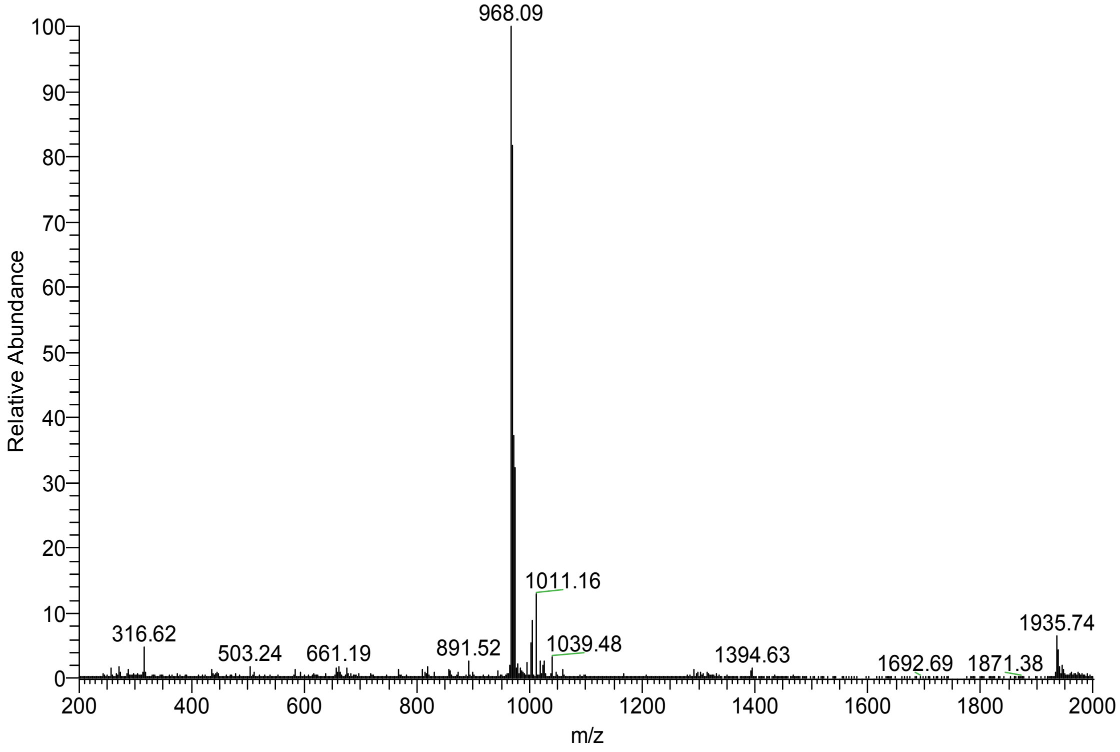
968.09
1011.16
1039.48
316.62
503.24
661.19
891.52
1394.63
1692.69
1871.38
1935.74
Relative Abundance
m/z

## Compound 21.

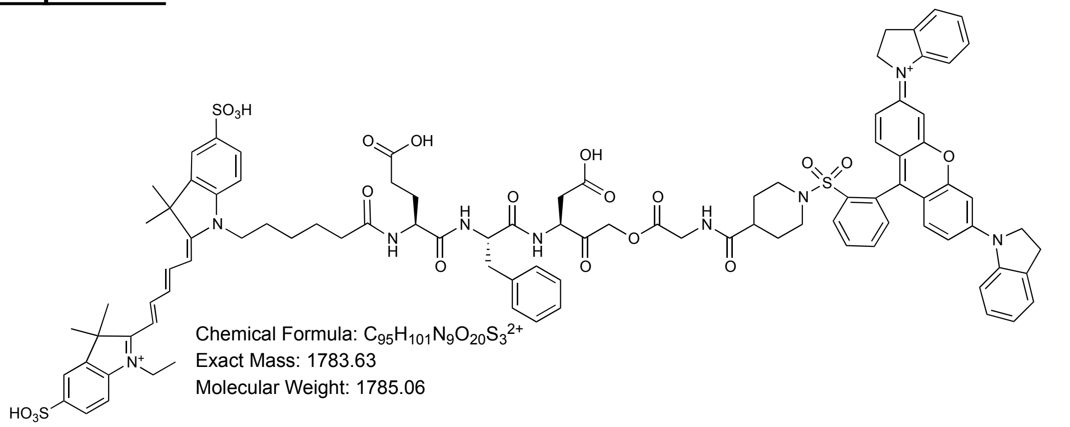
SO3H
HO3S
OH
N+
N
O
Chemical Formula: $C_{95}H_{101}N_9O_{20}S_3^{2+}$
Exact Mass: 1783.63
Molecular Weight: 1785.06

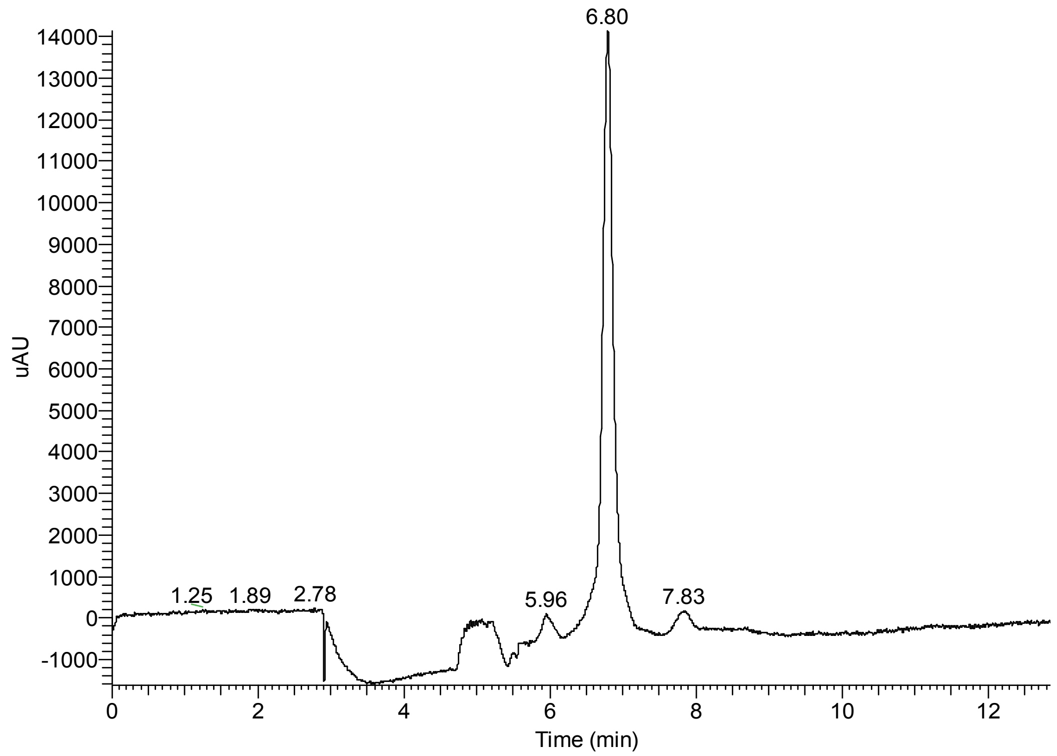
uAU
Time (min)
6.80
1.25
1.89
2.78
5.96
7.83

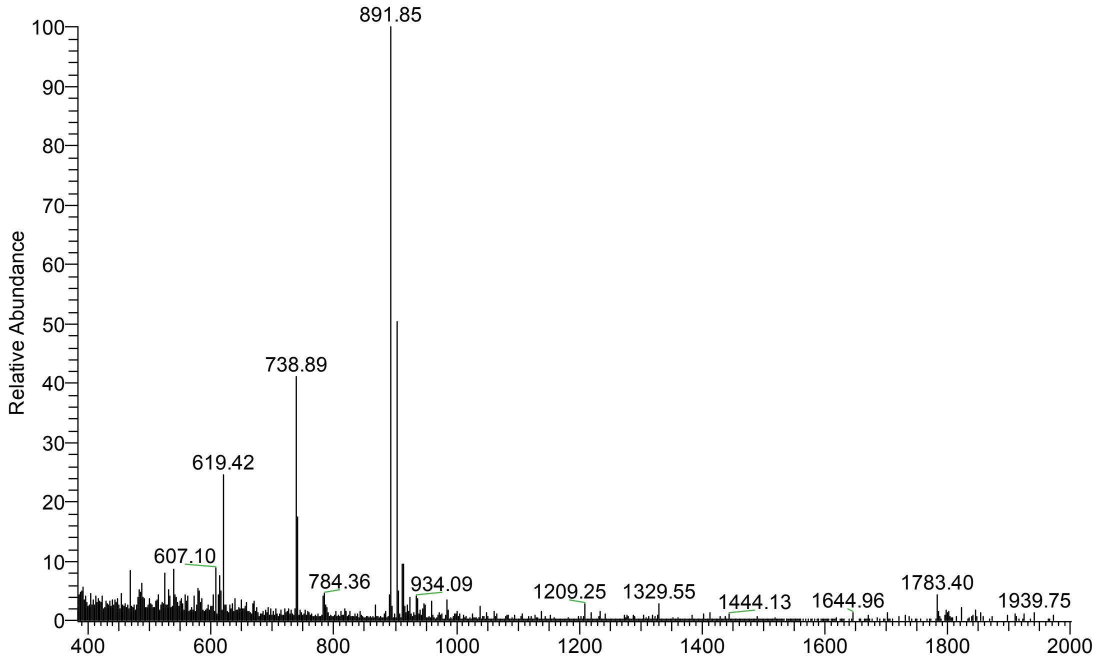
Relative Abundance
m/z
891.85
738.89
619.42
607.10
784.36
934.09
1209.25
1329.55
1444.13
1644.96
1783.40
1939.75
